# Features of 20 133 UK patients in hospital with covid-19 using the ISARIC WHO Clinical Characterisation Protocol: prospective observational cohort study

**DOI:** 10.1136/bmj.m1985

**Published:** 2020-05-22

**Authors:** Annemarie B Docherty, Ewen M Harrison, Christopher A Green, Hayley E Hardwick, Riinu Pius, Lisa Norman, Karl A Holden, Jonathan M Read, Frank Dondelinger, Gail Carson, Laura Merson, James Lee, Daniel Plotkin, Louise Sigfrid, Sophie Halpin, Clare Jackson, Carrol Gamble, Peter W Horby, Jonathan S Nguyen-Van-Tam, Antonia Ho, Clark D Russell, Jake Dunning, Peter JM Openshaw, J Kenneth Baillie, Malcolm G Semple

**Affiliations:** 1 Centre for Medical Informatics, Usher Institute, University of Edinburgh, Edinburgh, UK; 2 Intensive Care Unit, Royal Infirmary Edinburgh, Edinburgh, UK; 3 Institute of Microbiology and Infection, University of Birmingham, Birmingham, UK; 4 National Institute of Health Research (NIHR) Health Protection Research Unit in Emerging and Zoonotic Infections, Liverpool, UK; 5 Institute of Infection and Global Health, Faculty of Health and Life Sciences, University of Liverpool, Liverpool, UK; 6 Institute of Translational Medicine, Faculty of Health and Life Sciences, University of Liverpool, Liverpool, UK; 7 Centre for Health Informatics, Computing and Statistics, Lancaster Medical School, Lancaster University, Bailrigg, UK; 8 ISARIC Global Support Centre, Centre for Tropical Medicine and Global Health, Nuffield Department of Medicine, University of Oxford, Oxford, UK; 9 Infectious Diseases Data Observatory, Centre for Tropical Medicine and Global Health, University of Oxford, Oxford, UK; 10 Liverpool Clinical Trials Centre, University of Liverpool, Liverpool, UK; 11 Centre for Tropical Medicine and International Health, Nuffield Department of Medicine, University of Oxford, Oxford, UK; 12 Division of Epidemiology and Public Health, University of Nottingham School of Medicine, Nottingham, UK; 13 Medical Research Council University of Glasgow Centre for Virus Research, Glasgow, UK; 14 Queen’s Medical Research Institute, University of Edinburgh, Edinburgh, UK; 15 National Infection Service, Public Health England, London, UK; 16 Faculty of Medicine, Imperial College London, London, UK; 17 National Heart and Lung Institute, Faculty of Medicine, Imperial College London, London, UK; 18 Roslin Institute, University of Edinburgh, Edinburgh, UK; 19 NIHR Health Protection Research Unit in Emerging and Zoonotic Infections and Institute of Translational Medicine, Faculty of Health and Life Sciences, University of Liverpool, Liverpool, UK; 20 Respiratory Medicine, Alder Hey Children’s Hospital, Institute in The Park, University of Liverpool, Alder Hey Children’s Hospital, Liverpool L12 2AP, UK

## Abstract

**Objective:**

To characterise the clinical features of patients admitted to hospital with coronavirus disease 2019 (covid-19) in the United Kingdom during the growth phase of the first wave of this outbreak who were enrolled in the International Severe Acute Respiratory and emerging Infections Consortium (ISARIC) World Health Organization (WHO) Clinical Characterisation Protocol UK (CCP-UK) study, and to explore risk factors associated with mortality in hospital.

**Design:**

Prospective observational cohort study with rapid data gathering and near real time analysis.

**Setting:**

208 acute care hospitals in England, Wales, and Scotland between 6 February and 19 April 2020. A case report form developed by ISARIC and WHO was used to collect clinical data. A minimal follow-up time of two weeks (to 3 May 2020) allowed most patients to complete their hospital admission.

**Participants:**

20 133 hospital inpatients with covid-19.

**Main outcome measures:**

Admission to critical care (high dependency unit or intensive care unit) and mortality in hospital.

**Results:**

The median age of patients admitted to hospital with covid-19, or with a diagnosis of covid-19 made in hospital, was 73 years (interquartile range 58-82, range 0-104). More men were admitted than women (men 60%, n=12 068; women 40%, n=8065). The median duration of symptoms before admission was 4 days (interquartile range 1-8). The commonest comorbidities were chronic cardiac disease (31%, 5469/17 702), uncomplicated diabetes (21%, 3650/17 599), non-asthmatic chronic pulmonary disease (18%, 3128/17 634), and chronic kidney disease (16%, 2830/17 506); 23% (4161/18 525) had no reported major comorbidity. Overall, 41% (8199/20 133) of patients were discharged alive, 26% (5165/20 133) died, and 34% (6769/20 133) continued to receive care at the reporting date. 17% (3001/18 183) required admission to high dependency or intensive care units; of these, 28% (826/3001) were discharged alive, 32% (958/3001) died, and 41% (1217/3001) continued to receive care at the reporting date. Of those receiving mechanical ventilation, 17% (276/1658) were discharged alive, 37% (618/1658) died, and 46% (764/1658) remained in hospital. Increasing age, male sex, and comorbidities including chronic cardiac disease, non-asthmatic chronic pulmonary disease, chronic kidney disease, liver disease and obesity were associated with higher mortality in hospital.

**Conclusions:**

ISARIC WHO CCP-UK is a large prospective cohort study of patients in hospital with covid-19. The study continues to enrol at the time of this report. In study participants, mortality was high, independent risk factors were increasing age, male sex, and chronic comorbidity, including obesity. This study has shown the importance of pandemic preparedness and the need to maintain readiness to launch research studies in response to outbreaks.

**Study registration:**

ISRCTN66726260.

## Introduction

The outbreak of disease caused by the novel severe acute respiratory syndrome coronavirus 2 (SARS-CoV-2) was declared a pandemic by the World Health Organization on 11 March 2020.^1^ The WHO situation report dated 30 April 2020 stated 3 090 445 people had confirmed coronavirus disease 2019 (covid-19) and 217 769 people had died across the world.^2^

In the wake of the influenza A H1N1 pandemic (2009) and the emergence of Middle East respiratory syndrome coronavirus (2012), it was recognised that the effectiveness of a response to a future pandemic threat would critically depend on the speed and focus of that response. The United Kingdom set up and maintained a “sleeping” prepandemic suite of protocols, documents, and agreements in preparation for future outbreaks. The International Severe Acute Respiratory and emerging Infections Consortium (ISARIC) WHO Clinical Characterisation Protocol UK (CCP-UK) study was a core component of this portfolio.^3^ Further details about ISARIC WHO CCP-UK can be found at https://isaric4c.net and in the online supplement.

In response to the emergence of SARS-CoV-2 and its pandemic potential, the ISARIC WHO CCP-UK study was activated on 17 January 2020, in time to enrol the first wave of patients with covid-19 admitted to hospitals in England and Wales. The first confirmed patient with covid-19 in the UK was reported on 31 January 2020.

Hospital admission rates for patients with covid-19 have been difficult to estimate because rates depend on the prevalence of community testing and admission criteria, which vary between countries. However, an estimated one in 10 to one in five adults have illnesses of sufficient severity to warrant hospital admission.^4^ Patients have mostly been admitted with severe acute respiratory infection or severe acute respiratory syndrome according to the previous WHO case definitions.^5^ ^6^ The provision of intensive care also varies between countries. Studies first from China, and more recently from Europe and the United States, have found rates of admission to intensive care range from 5% to 32%.^7^ ^8^ Old age, chronic major comorbidity, and male sex have consistently been associated with increased mortality.^9^ ^10^ ^11^ ^12^

In this first report of the ISARIC WHO CCP-UK study, we characterise the clinical features of patients admitted to hospital with covid-19 in England, Scotland, and Wales during the growth phase of the first wave of this outbreak, up to 19 April 2020. Future reports will include Northern Ireland. We describe all patient outcomes as known on 3 May 2020 and explore risk factors associated with mortality in hospital.

## Methods

### Study design and setting

The ISARIC WHO CCP-UK (National Institute for Health Research Clinical Research Network Central Portfolio Management System ID: 14152) study is an ongoing prospective cohort study in 208 acute care hospitals in England, Scotland, and Wales. The protocol (supplementary material 2), revision history, case report form (version 9.2; supplementary material 3), information leaflets, consent forms and details of the Independent Data and Material Access Committee are available online.^21^

### Participants

Inclusion criteria were people of all ages who were admitted to one of 208 acute care hospitals in England, Scotland, and Wales with proven or high likelihood of infection with a pathogen of public health interest, defined as SARS-CoV-2 for this event by Public Health England. Reverse transcriptase polymerase chain reaction was the only mode of testing available during the period of study. The decision to test was at the discretion of the clinician attending the patient, and not defined by protocol. The enrolment criterion “high likelihood of infection” reflects that a preparedness protocol cannot assume that a diagnostic test will be available for an emergent pathogen. Site training emphasises that only patients who tested positive for covid-19 were eligible for enrolment.

National guidance was provided by Public Health England and other UK public health agencies that advised who to test based on clinical case definitions for possible covid-19 (online supplement). We also included patients who had been admitted for a separate condition but had tested positive for covid-19 during their hospital stay. We collected additional biological samples for research purposes when consent was given (please see online supplement for details of consent procedures and biological samples). These samples are currently undergoing analysis and we will present the results when they become available. Patients were only enrolled during their index admission. We used three tiers in the ISARIC WHO CCP-UK protocol. Patients in tier 0 had clinical information from their routine health records uploaded into the case report form. Consent was not required for collection of depersonalised routine healthcare data for research in England and Wales. A waiver for consent was given by the Public Benefit and Privacy Panel in Scotland. Tier 1 and 2 of the protocol involve additional biological sampling for research purposes for which consent by, or assent for, participants was obtained.

### Data collection

We collected baseline demographic data on a paper case report form (version 9.2; supplementary material 2) that was developed by ISARIC and WHO for use in outbreak investigations. Data were uploaded from admission, and usually before hospital episodes were complete, to a REDCap database (Research Electronic Data Capture, Vanderbilt University, US, hosted by University of Oxford, UK). We aimed to record measures of illness severity and routine blood test results at a minimum of four time points: day of hospital admission (day 1), day 3, day 6, day 9, and day of any admission to critical care. We recorded relevant treatments that patients received in hospital, level of care (ward based, high dependency unit, or intensive care unit), complications, and details of discharge or death while in hospital. Further information about these variables can be found in the online supplement.

### Outcomes

The main outcomes were critical care admission (high dependency unit or intensive care unit) and mortality in hospital or palliative discharge. We chose a priori to restrict analysis of outcomes to patients who were admitted more than two weeks before data extraction (3 May 2020) to enable most patients to finish their hospital admission.

### Bias

Research nurses relied on local covid-19 test reports to enrol patients. Capacity to enrol was limited by staff resources at times of high covid-19 activity. Otherwise we are unable to comment on the potential selection bias of our cohort. We are in the process of linking to routine administrative healthcare data and will be able to make comparisons at that point.

### Missing data

The nature of the study means that a large amount of data were missing, particularly during the later parts of the growth curve of the UK outbreak. Because this paper is mainly descriptive, we have not performed any imputation for missing data, and describe the data as they stand. To reduce the impact of missing data on outcome analyses, we restricted these analyses to patients who had been admitted for at least two weeks before data extraction.

### Statistical analyses

Continuous data are summarised as median (interquartile range) and categorical data as frequency (percentage). For univariate comparisons, the Mann-Whitney U test or Kruskal-Wallis test were used. We compared categorical data by using the χ^2^ test.

We used several approaches to model survival. Discharge from hospital was considered an absorbing state, meaning that once discharged, patients were considered no longer at risk of death. Patients who were discharged were not censored and held within the risk set, therefore accounting for the competing risk of discharge on death. We checked this approach by using a formal Fine and Gray competing risks approach. Hierarchical Cox proportional hazards approaches included geographical region (clinical commissioning group or health board) as a random intercept. We used a parsimonious criterion based model building approach based on several principles: clinically relevant explanatory variables were identified a priori for exploration; population stratification was incorporated; interactions were checked at first order level; final model selection was informed by log likelihood tests and the concordance statistic, with appropriate assumptions checked including the distribution of residuals and requirement for proportional hazards. We set statistical significance at 5%. All tests were two sided. We analysed data by using R (R Core Team version 3.6.3, Vienna, Austria), with packages including tidyverse, finalfit, survival, cmprsk, and coxme.

### Patient and public involvement

This was an urgent public health research study in response to a Public Health Emergency of International Concern. Patients or the public were not involved in the design, conduct, or reporting of this rapid response research.

## Results

On behalf of ISARIC WHO CCP-UK, 2468 research nurses, administrators, and medical students enrolled 20 133 patients who were admitted with covid-19 to 208 hospitals in England, Scotland, and Wales between 6 February and 14:00 on 19 April 2020 (table 1 and fig E1). This figure represents 34% of the 59 215 covid-19 admissions in these countries. The median time from onset of symptoms of covid-19 in the community to presentation at hospital was 4 days (interquartile range 1-8; n=16 221).

**Table 1:** Baseline characteristics of 20 133 patients with coronavirus disease 2019 stratified by sex. Data are numbers (percentages) unless stated otherwise

Characteristics	Male	Female	All
Total No (%)	12 068 (59.9)	8065 (40.1)	20 133
Age at admission (n=20 133)			
Median (interquartile range)	72.0 (58.0-81.0)	74.0 (58.0-84.0)	72.9 (58.0-82.0)
Age (n=20 133)			
<18	180 (1.5)	130 (1.6)	310 (1.5)
18-39	534 (4.4)	533 (6.6)	1067 (5.3)
40-50	888 (7.4)	530 (6.6)	1418 (7.0)
50-59	1728 (14.3)	980 (12.2)	2708 (13.5)
60-69	2115 (17.5)	1181 (14.6)	3296 (16.4)
70-79	2972 (24.6)	1720 (21.3)	4692 (23.3)
≥80	3651 (30.3)	2991 (37.1)	6642 (33.0)
Any comorbidity (n=18 525)			
No	2591 (23.4)	1570 (21.1)	4161 (22.5)
Yes	8492 (76.6)	5872 (78.9)	14 364 (77.5)
Chronic cardiac disease (n=17 702)			
No	7086 (66.8)	5147 (72.6)	12 233 (69.1)
Yes	3527 (33.2)	1942 (27.4)	5469 (30.9)
Chronic pulmonary disease, not asthma (n=17 634)			
No	8616 (81.7)	5890 (83.1)	14 506 (82.3)
Yes	1931 (18.3)	1197 (16.9)	3128 (17.7)
Asthma (n=17 535)			
No	9274 (88.6)	5721 (80.9)	14 995 (85.5)
Yes	1192 (11.4)	1348 (19.1)	2540 (14.5)
Smoker (n=14 184)			
Never smoked	5030 (58.8)	3938 (69.9)	8968 (63.2)
Former smoker	2972 (34.8)	1392 (24.7)	4364 (30.8)
Yes	549 (6.4)	303 (5.4)	852 (6.0)
Chronic kidney disease (n=17 506)			
No	8792 (84.0)	5884 (83.5)	14 676 (83.8)
Yes	1671 (16.0)	1159 (16.5)	2830 (16.2)
Diabetes without complications (n=17 599)			
No	8254 (78.3)	5695 (80.7)	13 949 (79.3)
Yes	2290 (21.7)	1360 (19.3)	3650 (20.7)
Diabetes with complications (n=17 516)			
No	9628 (91.8)	6589 (93.8)	16 217 (92.6)
Yes	860 (8.2)	439 (6.2)	1299 (7.4)
Obesity (n=16 081)			
No	8725 (90.6)	5671 (87.8)	14 396 (89.5)
Yes	900 (9.4)	785 (12.2)	1685 (10.5)
Chronic neurological disorder (n=17 382)			
No	9222 (88.6)	6189 (88.7)	15 411 (88.7)
Yes	1181 (11.4)	790 (11.3)	1971 (11.3)
Dementia (n=17 459)			
No	9211 (88.2)	5888 (83.9)	15 099 (86.5)
Yes	1232 (11.8)	1128 (16.1)	2360 (13.5)
Malignancy (n=17 354)			
No	9251 (89.2)	6360 (91.0)	15 611 (90.0)
Yes	1117 (10.8)	626 (9.0)	1743 (10.0)
Moderate or severe liver disease (n=17 360)			
No	10 181 (98.0)	6869 (98.5)	17 050 (98.2)
Yes	204 (2.0)	106 (1.5)	310 (1.8)
Mild liver disease (n=17 331)			
No	10 195 (98.3)	6855 (98.5)	17 050 (98.4)
Yes	174 (1.7)	107 (1.5)	281 (1.6)
Chronic haematological disease (n=17 328)			
No	9951 (96.0)	6684 (96.0)	16 635 (96.0)
Yes	415 (4.0)	278 (4.0)	693 (4.0)
Rheumatological disorder (n=17 289)			
No	9562 (92.4)	6031 (86.9)	15 593 (90.2)
Yes	787 (7.6)	909 (13.1)	1696 (9.8)
Malnutrition (n=16 695)			
No	9768 (97.8)	6531 (97.4)	16 299 (97.6)
Yes	222 (2.2)	174 (2.6)	396 (2.4)
Previous immunosuppressant drug treatment (n=18 009)			
Yes	876 (8.1)	791 (11.0)	1667 (9.3)
No	9339 (86.6)	6032 (83.5)	15 371 (85.4)
Not applicable	573 (5.3)	398 (5.5)	971 (5.4)
Previous anti-infective treatment (n=18 017)			
No	1940 (18.0)	1311 (18.2)	3251 (18.0)
Yes	8285 (76.8)	5520 (76.4)	13 805 (76.6)
Not applicable	569 (5.3)	392 (5.4)	961 (5.3)
AIDS/HIV (n=17 251)			
No	10 259 (99.5)	6909 (99.6)	17 168 (99.5)
Yes	55 (0.5)	28 (0.4)	83 (0.5)

### Age and sex

**Fig 1:**
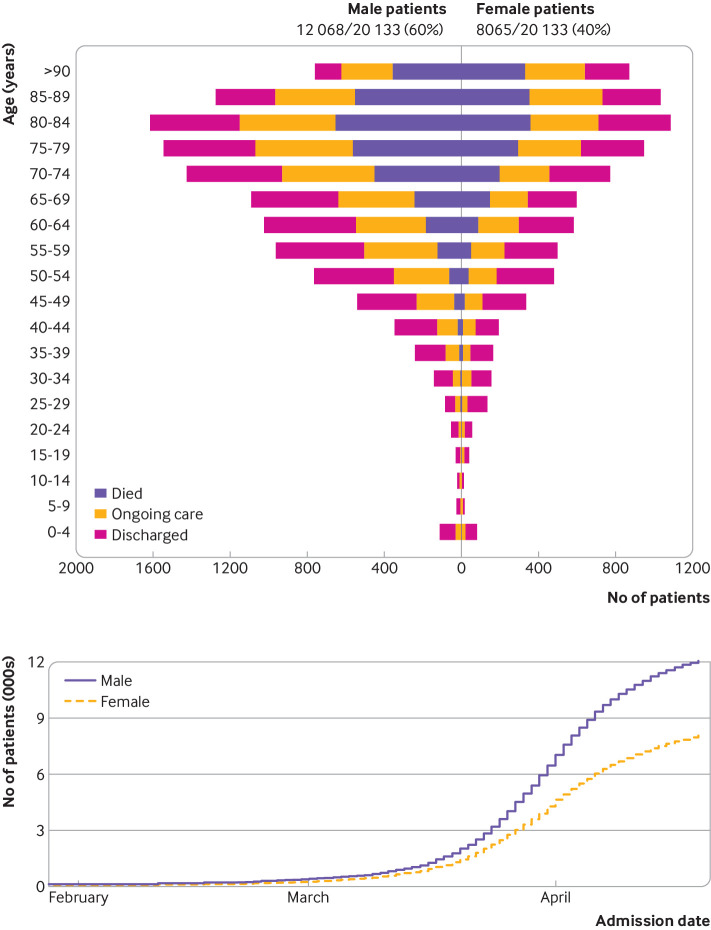
Patients with coronavirus disease 2019 (covid-19) stratified by age and sex (top panel), and date of hospital admission with covid-19 by sex (lower panel). Outcomes are discharge from hospital, ongoing care, and death at time of report (19 April 2020, n=20 133)

The median age of patients was 73 years (interquartile range 58-82, range 0-104; fig 1); 310 patients (1.5%) were less than 18 years old and 194 (1.0%) were less than 5 years old. More men (59.9%, n=12 068) than women (40.1%, n=8065) were admitted to hospital with covid-19. One hundred women (10%) of reproductive age (n=1033) were recorded as being pregnant.

### Symptoms

**Fig 2:**
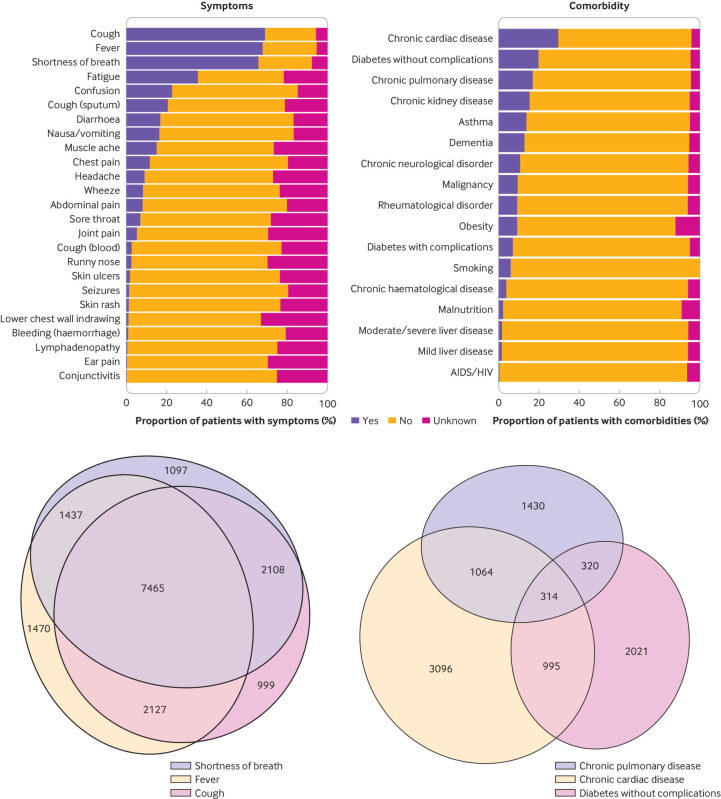
Presenting symptoms and comorbidities in patients in hospital with coronavirus disease 2019 (covid-19). Top left panel: symptoms by frequency of presentation (see table E1 for values); lower left panel: scaled Euler diagram of overlap of commonest symptoms; top right panel: comorbidities by frequency (see table 1 for values); lower right panel: scaled Euler diagram of overlap of commonest comorbidities

The most common symptoms were cough (68.9%, 12 896/18 730), fever (71.6%, 12 499/17 452), and shortness of breath (71.2%, 12 107/16 999; fig 2, top left panel), though these data reflect the case definition. Only 4.5% (855/19 178) of patients reported no symptoms on admission. We found a high degree of overlap between the three most common symptoms (fig 2, lower left panel).

Clusters of symptoms on admission were apparent (fig E2). The most common symptom cluster encompassed the respiratory system: cough, sputum, shortness of breath, and fever. We also observed three other clusters: one encompassing musculoskeletal symptoms (myalgia, joint pain, headache, and fatigue); a cluster of enteric symptoms (abdominal pain, vomiting, and diarrhoea); and less commonly, a mucocutaneous cluster. Twenty nine per cent (5384/18 605) of all patients complained of enteric symptoms on admission, mostly in association with respiratory symptoms; however, 4% of all patients described enteric symptoms alone.

### Comorbidities

Figure 2 (top right panel) and table 1 show major comorbidities recorded on admission. The most common major comorbidities were chronic cardiac disease (30.9%, 5469/17 702), diabetes without complications (20.7%, 3650/17 599), chronic pulmonary disease excluding asthma (17.7%, 3128/17 634), chronic kidney disease (16.2%, 2830/17 506), and asthma (14.5%, 2540/17 535). Of 18 525 patients, 22.5% (4161) had no documented major comorbidity. There was little overlap between the three most common comorbidities (fig 2, lower right panel). 

Six per cent (852/14 184) of patients were current smokers, 30.8% (4364) were previous smokers, and 63.2% (8968) had never smoked. Figure E3 shows the pattern of major comorbidity stratified by age.

### Level of care

**Fig 3:**
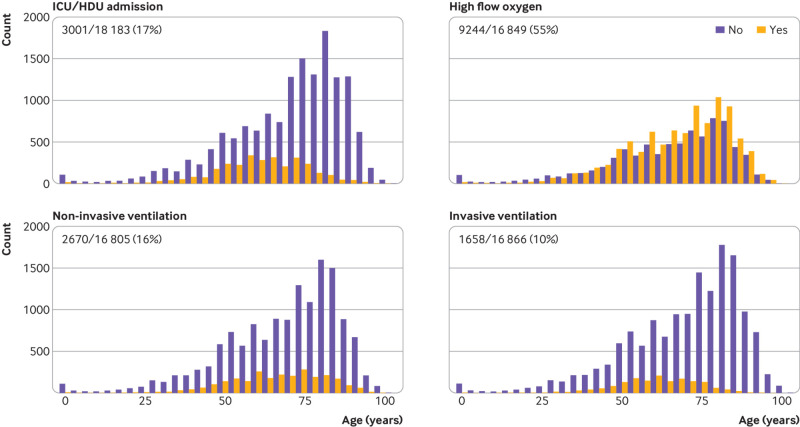
Level of care stratified by age: admitted to intensive care unit (ICU) or high dependency unit (HDU), high flow oxygen, non-invasive ventilation, and invasive ventilation

A high proportion of patients required admission to high dependency or intensive care units (17%, 3001/18 183; fig 3), and 55% (9244/16 849) received high flow oxygen at some point during their admission. Sixteen per cent of patients (2670/16 805) were treated with non-invasive ventilation, while 10% (1658/16 866) received invasive ventilation.

### Patient outcomes

**Fig 4:**
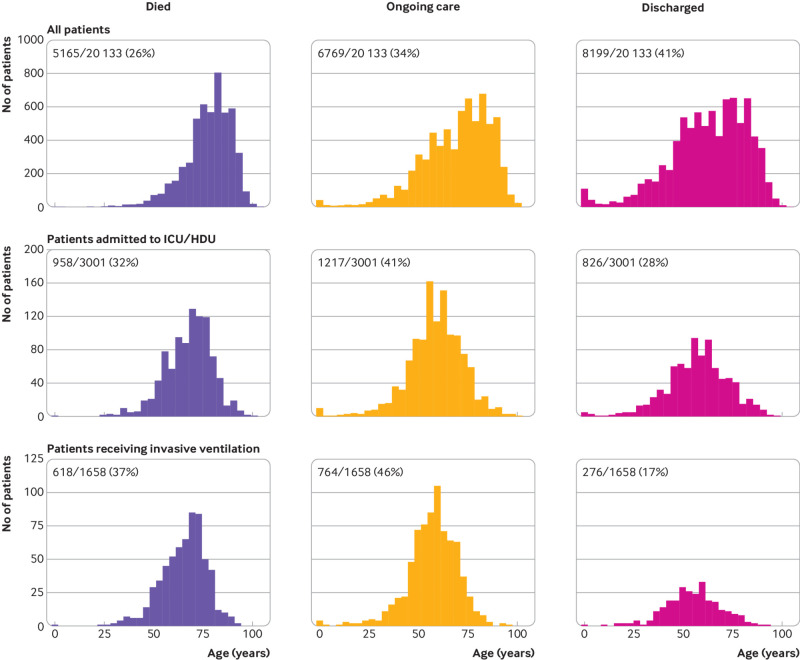
Status of patients at time of reporting stratified by level of care. Top panel: all patients in hospital with coronavirus disease 2019 (covid-19); middle panel: all patients admitted to intensive care unit (ICU) or high dependency unit (HDU); lower panel: patients receiving invasive mechanical ventilation

Overall, 41% (8199/20 133) of patients were discharged alive, 26% (5165/20 133) died, and 34% (6769/20 133) continued to receive care at the date of reporting (fig 4). The median age of patients who died in hospital from covid-19 in the study was 80 years, and only 11% (559/4880) of these patients had no documented major comorbidity.

For patients who received only ward care, 47% (7203/15 297) were discharged alive, 26% (3954/15 297) died, and 27% (4140/15 297) remained in hospital at the date of reporting. As expected, outcomes were worse for those who needed higher levels of care.

Of patients admitted to critical care (high dependency unit or intensive care unit), 28% (826/3001) were discharged alive, 32% (958/3001) died, and 41% (1217/3001) continued to receive care at the date of reporting. Although the patients who received mechanical ventilation were younger than the overall cohort (61 years, interquartile range 52-69), only 17% (276/1658) had been discharged alive by 19 April 2020, 37% (618/1658) had died, and 46% (764/1658) continued to receive care.

Length of stay increased with age for patients discharged alive (fig E4). For patients who died, we found no association between age and time to death, with around 80% dying before day 14 of hospital admission.

### Association of pre-existing patient characteristics and survival

**Fig 5:**
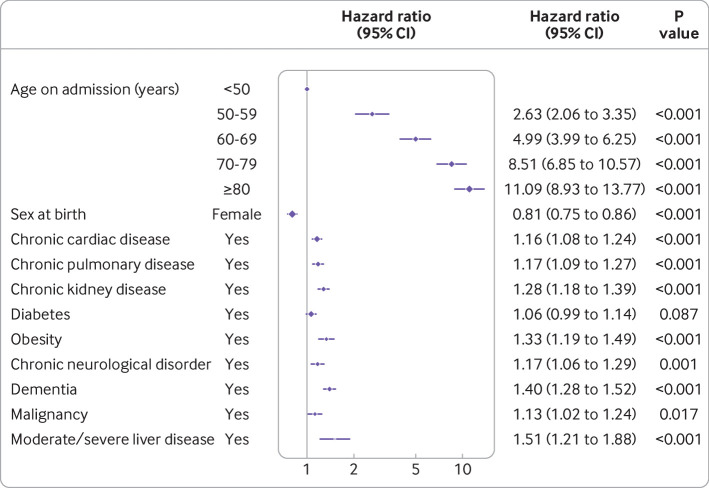
Multivariable Cox proportional hazards model (age, sex, and major comorbidities), where hazard is death. Patients who were discharged were kept in the risk set (n=15 194; No of events=3911)

The online supplement (table E4) describes univariable and multivariable associations with mortality. Figure 5 shows variables that remained significant in the multivariable model. Increasing age was a strong predictor of mortality in hospital after adjusting for major comorbidity (reference age <50 years): 50-59 years, hazard ratio 2.63 (95% confidence interval 2.06 to 3.35, P<0.001); 60-69 years, 4.99 (3.99 to 6.25, P<0.001); 70-79 years, 8.51 (6.85 to 10.57, P<0.001); ≥80 years, 11.09 (8.93 to 13.77, P<0.001). Female sex was associated with lower mortality (0.81, 0.75 to 0.86, P<0.001). Chronic cardiac disease, chronic non-asthmatic pulmonary disease, chronic kidney disease, obesity, chronic neurological disorder (such as stroke), dementia, malignancy, and liver disease were also associated with increased hospital mortality. An interactive infographic is available at https://isaric4c.net/info. This information must not be used as a predictive tool in practice or to inform individual treatment decisions.

## Discussion

### Principal findings

Patients with covid-19 usually presented with fever, cough, and shortness of breath, and met the WHO case definitions for severe acute respiratory infection or severe acute respiratory syndrome. The most common previous major comorbidities were chronic cardiac disease, diabetes, and chronic non-asthmatic pulmonary disease. Seventeen per cent of patients were admitted to critical care (high dependency unit or intensive care unit). Mortality in hospital was at least 26%, with 34% of the cohort still in hospital at the time of analysis; these proportions increased with escalating level of care. Factors associated with mortality in hospital were increasing age, male sex, and major comorbidities (cardiac disease, non-asthmatic pulmonary disease, kidney disease, liver disease, malignancy, obesity, and dementia).

The data presented in this study describe patients admitted to hospital during the growth phase of the SARS-CoV-2 pandemic in the UK. The first 101 patients were enrolled in the early phase of the outbreak as part of a high consequence infectious disease containment strategy that ended on 10 March 2020. These patients and others who were identified through screening in hospital, or who contracted covid-19 after admission (hospital acquired infection), are included in the 855 patients who were admitted without covid-19 symptoms. The impact these patients have had on the overall cohort characteristics has diminished as numbers have increased, and we believe it is important to keep these patients in the study. Other patients in our cohort without covid-19 symptoms are those who were diagnosed with the disease at the discretion of the clinician looking after them while staying in hospital for other reasons.

The pattern of disease we describe broadly reflects the pattern reported globally.^7^ Patients in our study had a higher median age and higher rates of chronic obstructive pulmonary disease and asthma than patients in China^8^ and the US.^11^ ^12^ The prevalence of obesity in our study (11%) was considerably lower than the overall UK prevalence (29%).^13^ This proportion could reflect the relatively elderly male population admitted to hospital and misclassification or under reporting by admitting physicians. Our patients presented with a relatively short time interval between onset of symptoms and admission to hospital, which might also be a function of the older and vulnerable patient population.

The current case definition of cough and fever, if strictly applied, would miss 7% of our inpatients. A smaller proportion, 4% of patients, presented with enteric symptoms only. This figure could be an underestimate because these patients fall outside standard criteria for testing. This enteric presentation risks misclassification of patients, and assignment to non-covid-19 care areas, which could pose a nosocomial transmission risk. Severe SARS-CoV-2 infections are rare in people younger than 18 years, comprising only 1.5% of those admitted to hospital. Only 1.0% of those in our study were younger than 5 years. The J shaped age distribution is starkly different to the U shaped age distribution seen in seasonal influenza and the W shaped distribution observed in the 2009 influenza pandemic.^14^ The reason why SARS-CoV-19 has mostly spared children is not clear, but we speculate this could be because angiotensin converting enzyme 2 receptors are expressed differently in younger lungs.

Other studies have not widely reported that obesity as recognised by clinical staff is associated with mortality in hospital after adjustment for other comorbidities, age, and sex. Obesity was recognised as a risk factor in the 2009 influenza A H1N1 pandemic, but not for the 2012 Middle East respiratory syndrome coronavirus.^15^ ^16^

The proportion of pregnant women in our cohort was small (10%), similar to the estimated proportion of pregnant women in the community.^15^ Pregnancy was not associated with mortality, in apparent contrast to influenza.^17^

### Comparison with other studies

The proportion of patients admitted to critical care in our study was similar to that reported in Italy (17%),^18^ ^19^ and New York (14.2%),^11^ ^12^ but higher than China.^8^ At the time of enrolment, the Intensive Care Society had issued guidance to its members that there would be no rationing of critical care admission until all capacity in the country had been exhausted. As far as we are aware, critical care capacity was not exceeded in the UK during the period of the study. We do not believe that any equipment shortages existed during this period that might have prompted more aggressive futility discussions.

Mortality in our cohort was high in patients admitted to general wards who were not admitted to critical care, which suggests that advanced care planning occurred. We were unable to capture treatment limiting decisions about level of care. The high median age of patients who died in the cohort (80 years) could partly explain the high mortality rate. Mortality rates were extremely high for patients who received invasive mechanical ventilation in the intensive care unit compared with the 2009 influenza A H1N1 pandemic, for which mortality in intensive care was 31%.^15^ Our data were in line with the initial ICNARC (intensive care national audit and research centre) audit reports, which represent intensive care units in England, Wales, Scotland, and Northern Ireland.^20^

Outcome analyses only included patients who were admitted before 19 April to allow most patients to complete their hospital admission. However, an inherent reporting bias exists because the sickest of patients, particularly those admitted to intensive care, have the longest hospital stays; mortality rates in hospital could therefore increase. These mortality rates were considerably higher than the 24% mortality rate in hospital seen in patients in intensive care units in Italy^19^ and the US.^11^ ^12^ The lower rate in the US could in part be explained by differences in healthcare systems and the proportion of intensive care unit beds to hospital beds between the two countries. In Italy, a lower proportion of patients received mechanical ventilation, and most of their patients (72%) remained in hospital at the time of the analysis.^19^

The finding of independent associations of advancing age, male sex, chronic respiratory (non-asthmatic) disease, chronic cardiac disease, and chronic neurological disease with mortality in hospital is in line with early international reports.^9^ ^10^ However, although age adjusted mortality rates are high in elderly patients, most of these patients were admitted to hospital with symptoms of covid-19 and would not have been in hospital otherwise. Enhanced severity in male patients was seen across all ages.

### Strengths and limitations of study

ISARIC WHO CCP-UK stood ready to conduct large scale studies of pandemic outbreaks for eight years, enabling us to enrol 34% of all patients with covid-19 admitted to 208 acute care hospitals across England, Wales and Scotland in the early phase of the pandemic.

Our study has some limitations. We do not currently have data on the inpatients that were not enrolled, or people managed in community settings, such as usual domestic residences and older people’s care homes. We are unable to comment on community risk factors that drive hospital admission except by inference from expected representation at admission. We will be linking to routine administrative healthcare datasets which will enable us to assess the presence of any selection bias.

A large amount of data were missing and we suggest there are two main reasons for this. Firstly, enrolment occurred in the nonlinear growth phase of the outbreak, and outcomes for recent admissions have not been reported yet; these admissions account for 18% of the total number of patients enrolled. Secondly, the research network was dealing with unprecedented numbers of patients at a time when many were seconded to clinical practice or themselves off sick. This study is ongoing, and further data are being added to case report forms.

We suggest it is possible that the sickest patients were enrolled in our study, and this could partly explain our high mortality rates in hospital. Some of the sickest patients in the study had the longest lengths of hospital stay and we do not have outcome data for all of these patients yet.

### Conclusions and policy implications

This large and rapidly conducted study of patients admitted to hospital in England, Wales, and Scotland with covid-19 shows the importance of putting plans in place for the study of epidemic and pandemic threats, and the need to maintain these plans. Our study identifies sectors of the population that are at greatest risk of a poor outcome, and reports the use of healthcare resources. Most patients with covid-19 experience mild disease. However, in our cohort, of those who were admitted to hospital two weeks before data extraction, less than half have been discharged alive and a quarter have died. The remainder continued to receive care at the date of reporting. Seventeen percent of patients admitted to hospital required critical care. Factors associated with mortality in hospital were increasing age, male sex, obesity, and major comorbidities.

ISARIC Coronavirus Clinical Characterisation Consortium^21^ investigators have submitted regular reports to the UK Government’s New and Emerging Respiratory Virus Threats Advisory Group (NERVTAG)^22^ and the Scientific Advisory Group for Emergencies (SAGE).^23^ Patient level data have been shared and independently analysed by the Scientific Pandemic Influenza Group on Modelling (SPI-M)^24^ and other investigators. Aggregated data have been shared with WHO in the ISARIC covid-19 report.

Studies such as this cannot be developed, approved, and opened from the start of a pandemic in time to inform case management and public health policy. Our study has shown the importance of forward planning and investment in preparedness studies. Over the next few months we will issue reports in *The BMJ* on specific topics and analyses that are key to understanding the impact of covid-19 and focus on improving patient outcomes.

**What is already known on this topic**

Observational studies in China have reported risk factors associated with severe covid-19 that requires hospital admission
Studies describing the features and outcomes of patients with severe covid-19 who have been admitted to hospital in Europe are lacking
Older male adults, people with diabetes, hypertension, cardiovascular disease, or chronic respiratory disease are at greater risk of severe covid-19 that requires hospital admission and higher levels of care, and are at higher risk of death

**What this study adds**

This rapid prospective investigation of patients with covid-19 admitted to hospital in England, Wales, and Scotland showed that obesity, chronic kidney disease, and liver disease were also associated with increased hospital mortality
Obesity is a major additional risk factor that was not highlighted in data from China
Severe covid-19 leads to a prolonged hospital stay and a high mortality rate; over a quarter of inpatients in this study had died at the time of reporting, and nearly a third remained in hospital

## Web extra

Extra material supplied by authors

Web appendix: Supplementary material 1—online supplement

Web appendix: Supplementary material 2—protocol

Web appendix: Supplementary material 3—case report form

## Notes

Contributors: Conceptualisation: JKB, JD, GC, LM, JSN-V-T, PJMO, MGS. Formal analysis: ABD, FD, CG, EMH, PWH, LN, PJMO, RP, JMR, MGS. Writing original draft: ABD, PJMO, MGS. Writing reviewing and editing: JKB, ABD, JD, CG, CAG, EMH, PWH, JSN-V-T, PJMO, MGS, LS. Project administration: SH, HEH, CG, AH, KAH, JL, LM, DP, CDR. Investigation: EMH, PWH, CG, CAG, AH, MGS. Supervision: JKB, HEH, EMH, CG, AH, PWH, PJMO, MGS. Data curation: LM, SH, CJ. Validation: KAH, SH, CJ. Funding acquisition: JKB, GC, PWH, PJMO, MGS. MGS is guarantor and corresponding author for this work, and attests that all listed authors meet authorship criteria and that no others meeting the criteria have been omitted.

Ethical approval: Ethical approval was given by the South Central - Oxford C Research Ethics Committee in England (Ref 13/SC/0149), the Scotland A Research Ethics Committee (Ref 20/SS/0028), and the WHO Ethics Review Committee (RPC571 and RPC572, 25 April 2013).

Data sharing: We welcome applications for data and material access through our Independent Data and Material Access Committee (https://isaric4c.net).

The lead author (the manuscript's guarantor) affirms that the manuscript is an honest, accurate, and transparent account of the study being reported; that no important aspects of the study have been omitted; and that any discrepancies from the study as planned (and, if relevant, registered) have been explained.

Dissemination to participants and related patient and public communities: ISARIC4C has a public facing website and twitter account @CCPUKstudy. We are engaging with print and internet press, television, radio, news, and documentary programme makers. We will explore distribution of findings with The Asthma UK and British Lung Foundation Partnership, and take advice from NIHR Involve and GenerationR Alliance Young People’s Advisory Groups.
