# Supplementary material for: Features of 20 133 UK patients in hospital with covid-19 using the ISARIC WHO Clinical Characterisation Protocol: prospective observational cohort study
Source: BMJ. 2020 May 22;369:m1985. doi: 10.1136/bmj.m1985 (PMC7243036; doi:10.1136/bmj.m1985)
Supplement: Supplementary file 1 — Web appendix: Supplementary material 1—online supplement [file doca057402.ww1.pdf]

Features of 20,133 hospitalised UK patients with COVID-19 using the ISARIC WHO Clinical Characterisation Protocol

AB Docherty, EM Harrison, CA Green, H Hardwick, R Pius, L Norman, KA Holden, JM Read, F Dondelinger, G Carson , L Merson L, L Lee , D Plotkin , L Sigfrid, S Halpin, C Jackson, C Gamble, PW Horby, JS Nguyen-Van-Tam, ISARIC4C Investigators, J Dunning, PJM Openshaw, JK Baillie, MG Semple.

Online supplement

Introduction

ISARIC's aim is to facilitate real-time research on diseases caused by novel pathogens of public health concern in order to save lives and inform public health policy early on and during outbreaks[1]. Our open-access protocols use standardised and refined case report forms, information and consent documents, and offer a tiered biological sampling schedule. This enables aggregation between sites and studies and allows comparisons to be made between diverse locations and treatment strategies[2].

ISARIC's protocols were assigned Urgent Public Health Research Status by the National Institute for Health Research (NIHR), acknowledging that National Health Service (NHS) Hospitals in England and Wales and NIHR Clinical Research Network (CRN) resources would be necessary to prioritise their use in the event of activation. The ISARIC protocol gained approval across all acute hospital trusts in England and Wales in 2013.

The World Health Organisation (WHO) Ethics Review Committee approved a global master protocol for the ISARIC CCP (RPC571 & RPC572 WHO Research Ethics Review Committee, 25/04/2013) and endorsed its use in outbreaks of public health interest. The ISARIC CCP study is international and now includes low- and middle-income countries. The protocol is known globally as the ISARIC-WHO Clinical Characterisation Protocol for Severe Emerging Infection and, in the UK, the ISARIC CCP-UK.

Methods

The clinical management of people with COVID-19 was not defined by the study.

Up to 10th March, test-positive symptomatic cases were admitted to hospital as part of a national containment strategy. Additionally, a small number of asymptomatic test-positive individuals were admitted to hospital; for example, asymptomatic travellers arriving in the UK who had been tested prior to departing countries that performed asymptomatic testing, with notification of UK authorities when the travellers arrived in the UK. After 10th March, admission was based on clinical need.

PHE published a case definition of COVID-19 on 13th March 2020 (PHE) at https://www.gov.uk/government/publications/wuhan-novel-coronavirus-initial-investigation-of-possible-cases/investigation-and-initial-clinical-management-of-possible-cases-of-wuhan-novel-coronavirus-wn-cov-infection

The clinical case definitions for hospital inpatients were clinical/radiological evidence of pneumonia or acute respiratory distress syndrome or influenza like illness (fever ≥37.8C and at least one of the following respiratory symptoms, which must be of acute onset: persistent cough (with or without sputum), hoarseness, nasal discharge or congestion, shortness of breath, sore throat, wheezing, sneezing.

Around 23rd March, many hospitals included peripheral oxygen saturations (SpO_2) of less than 93% as a threshold to inform admission decisions.

Testing

The criteria to test for SARS-CoV-2 by PCR were not defined by the study; the decision to test was at the discretion of the clinician attending the patient. To support this, national guidance was provided by PHE and other UK public health agencies that advises who to test based on clinical case definitions for possible COVID-19, and also prioritisation of testing when demand exceeds capacity. In reality, anyone who was admitted to hospital and met the case definition for possible COVID-19 would have been tested, as capacity was never so limiting that hospital patients had to be triaged for testing.

Variables

Age: Recorded as date of birth in consented Tier 1 and 2 patients, and age (years) in unconsented Tier 0 patients. We have categorised age into <50yrs, 50-69, 70-79 and >=80yrs for ease of clinical interpretation.

Sex: Categorised as sex at birth into Male vs Female

Comorbidities: as per Charlson Comorbidities Index. Obesity - clinician defined.

Day 1: Day of hospital admission for patients admitted with symptoms of COVID-19, and day that COVID-19 test was requested by clinicians for patients already admitted with alternative diagnosis

Critical Care: High Dependency or Intensive Care. Generally requiring additional organ support

Mortality: In-hospital mortality plus palliative discharge

Bias

The research nurses were not formally screening all hospital admissions. We are therefore relatively unable to comment on the potential selection bias of our cohort. We are in the process of linking to routine administrative healthcare data and will be able to make comparisons at that point. Certain cohorts such as children and maternity, may be underrepresented.

Study size

There was no formal power calculation for the study size. We report all patients recruited up to 19th April 2020, to allow 14 day outcomes to be described on 3rd of May, the date of data extraction.

Results

Table E1. Presentation of COVID-19 in hospitalised patients. The denominator for each symptom excludes patients who were not specifically declared "No" or "Yes" indicated in column "N".

	N		Male	Female	All
Total N (%)			12068 (59.9)	8065 (40.1)	20133
Onset to admission (days)	18406	Median (IQR)	5.0 (1.0 to 8.0)	4.0 (1.0 to 7.0)	4.0 (1.0 to 8.0)
Fever	17452	No	2817 (26.9)	2136 (30.6)	4953 (28.4)
		Yes	7652 (73.1)	4847 (69.4)	12499 (71.6)
Cough	18730	No	3378 (30.0)	2456 (32.9)	5834 (31.1)
		Yes	7876 (70.0)	5020 (67.1)	12896 (68.9)
Cough (sputum)	14413	No	6317 (73.1)	4322 (74.8)	10639 (73.8)
		Yes	2319 (26.9)	1455 (25.2)	3774 (26.2)
Cough (blood)	14082	No	8106 (96.0)	5486 (97.2)	13592 (96.5)
		Yes	334 (4.0)	156 (2.8)	490 (3.5)
Sore throat	13131	No	7092 (90.4)	4758 (90.0)	11850 (90.2)
		Yes	755 (9.6)	526 (10.0)	1281 (9.8)
Runny nose	12838	No	7403 (96.3)	4972 (96.5)	12375 (96.4)
		Yes	284 (3.7)	179 (3.5)	463 (3.6)
Ear pain	12852	No	7650 (99.5)	5124 (99.3)	12774 (99.4)
		Yes	41 (0.5)	37 (0.7)	78 (0.6)
Wheeze	13952	No	7503 (89.9)	4931 (88.0)	12434 (89.1)
		Yes	846 (10.1)	672 (12.0)	1518 (10.9)
Chest pain	14715	No	7627 (86.2)	4942 (84.2)	12569 (85.4)
		Yes	1221 (13.8)	925 (15.8)	2146 (14.6)
Muscle ache	13403	No	6397 (79.4)	4250 (79.6)	10647 (79.4)
		Yes	1664 (20.6)	1092 (20.4)	2756 (20.6)
Joint pain	12864	No	7161 (92.8)	4742 (92.1)	11903 (92.5)
		Yes	556 (7.2)	405 (7.9)	961 (7.5)

Fatigue	14318	No	4649 (54.1)	3156 (55.2)	7805 (54.5)
		Yes	3948 (45.9)	2565 (44.8)	6513 (45.5)
Shortness of breath	16999	No	2794 (27.4)	2098 (30.9)	4892 (28.8)
		Yes	7414 (72.6)	4693 (69.1)	12107 (71.2)
Lower chest wall indrawing	12235	No	7195 (98.3)	4847 (98.7)	12042 (98.4)
		Yes	127 (1.7)	66 (1.3)	193 (1.6)
Headache	13325	No	7066 (88.4)	4599 (86.3)	11665 (87.5)
		Yes	930 (11.6)	730 (13.7)	1660 (12.5)
Confusion	15609	No	6825 (72.7)	4613 (74.1)	11438 (73.3)
		Yes	2558 (27.3)	1613 (25.9)	4171 (26.7)
Seizures	14702	No	8670 (98.3)	5783 (98.4)	14453 (98.3)
		Yes	153 (1.7)	96 (1.6)	249 (1.7)
Abdominal pain	14616	No	7957 (90.7)	5174 (88.6)	13131 (89.8)
		Yes	819 (9.3)	666 (11.4)	1485 (10.2)
Nausea/vomiting	15241	No	7552 (82.7)	4677 (76.5)	12229 (80.2)
		Yes	1577 (17.3)	1435 (23.5)	3012 (19.8)
Diarrhoea	15214	No	7375 (80.7)	4742 (78.0)	12117 (79.6)
		Yes	1760 (19.3)	1337 (22.0)	3097 (20.4)
Conjunctivitis	13680	No	8172 (99.6)	5461 (99.7)	13633 (99.7)
		Yes	30 (0.4)	17 (0.3)	47 (0.3)
Skin rash	13994	No	8250 (98.3)	5512 (98.5)	13762 (98.3)
		Yes	146 (1.7)	86 (1.5)	232 (1.7)
Skin ulcers	13951	No	8201 (97.9)	5419 (97.3)	13620 (97.6)
		Yes	179 (2.1)	152 (2.7)	331 (2.4)
Lymphadenopathy	13711	No	8173 (99.3)	5447 (99.3)	13620 (99.3)
		Yes	55 (0.7)	36 (0.7)	91 (0.7)
Bleeding (Haemorrhage)	14470	No	8591 (98.8)	5705 (98.9)	14296 (98.8)

		Yes	108 (1.2)	66 (1.1)	174 (1.2)
Any symptom	19178	No	477 (4.1)	378 (4.9)	855 (4.5)
		Yes	11036 (95.9)	7287 (95.1)	18323 (95.5)

Table E2. Current status stratified by age and sex. Table matches values in Figure 1.

Sex at Birth	Age (y)	Discharged	On-going care	Died	All
Male	All	4711 (39.0)	4088 (33.9)	3269 (27.1)	12068
	0-4	83 (73.5)	29 (25.7)	1 (0.9)	113 (100)
	5-9	21 (80.8)	5 (19.2)	0 (0.0)	26 (100)
	10-14	12 (54.5)	10 (45.5)	0 (0.0)	22 (100)
	15-19	24 (80.0)	5 (16.7)	1 (3.3)	30 (100)
	20-24	40 (74.1)	13 (24.1)	1 (1.9)	54 (100)
	25-29	53 (62.4)	27 (31.8)	5 (5.9)	85 (100)
	30-34	99 (69.2)	39 (27.3)	5 (3.5)	143 (100)
	35-39	159 (66.0)	71 (29.5)	11 (4.6)	241 (100)
	40-44	222 (64.0)	106 (30.5)	19 (5.5)	347 (100)
	45-49	309 (57.1)	195 (36.0)	37 (6.8)	541 (100)
	50-54	415 (54.2)	287 (37.5)	63 (8.2)	765 (100)
	55-59	459 (47.7)	380 (39.5)	124 (12.9)	963 (100)
	60-64	477 (46.6)	363 (35.4)	184 (18.0)	1024 (100)
	65-69	453 (41.5)	395 (36.2)	243 (22.3)	1091 (100)
	70-74	496 (34.8)	479 (33.6)	451 (31.6)	1426 (100)
	75-79	477 (30.9)	506 (32.7)	563 (36.4)	1546 (100)
	80-84	466 (28.8)	497 (30.8)	653 (40.4)	1616 (100)
	85-89	309 (24.2)	414 (32.5)	552 (43.3)	1275 (100)
	90+	137 (18.0)	267 (35.1)	356 (46.8)	760 (100)
Female	All	3488 (43.2)	2681 (33.2)	1896 (23.5)	8065

	0-4	60 (74.1)	20 (24.7)	1 (1.2)	81 (100)
	5-9	10 (58.8)	7 (41.2)	0 (0.0)	17 (100)
	10-14	11 (84.6)	2 (15.4)	0 (0.0)	13 (100)
	15-19	25 (62.5)	14 (35.0)	1 (2.5)	40 (100)
	20-24	39 (69.6)	16 (28.6)	1 (1.8)	56 (100)
	25-29	104 (77.0)	28 (20.7)	3 (2.2)	135 (100)
	30-34	105 (67.3)	49 (31.4)	2 (1.3)	156 (100)
	35-39	119 (72.1)	38 (23.0)	8 (4.8)	165 (100)
	40-44	121 (62.4)	65 (33.5)	8 (4.1)	194 (100)
	45-49	227 (67.6)	92 (27.4)	17 (5.1)	336 (100)
	50-54	299 (62.2)	145 (30.1)	37 (7.7)	481 (100)
	55-59	276 (55.3)	173 (34.7)	50 (10.0)	499 (100)
	60-64	286 (49.1)	210 (36.0)	87 (14.9)	583 (100)
	65-69	254 (42.5)	196 (32.8)	148 (24.7)	598 (100)
	70-74	316 (40.9)	258 (33.4)	198 (25.6)	772 (100)
	75-79	328 (34.6)	326 (34.4)	294 (31.0)	948 (100)
	80-84	375 (34.5)	353 (32.5)	358 (33.0)	1086 (100)
	85-89	302 (29.2)	379 (36.7)	353 (34.1)	1034 (100)
	90+	231 (26.5)	310 (35.6)	330 (37.9)	871 (100)

Table E3. Current status by patient characteristics on admission.

	N		Died	On-going care	Discharged alive	All
Total N (%)			5165 (25.7)	6769 (33.6)	8199 (40.7)	20133
Age on admission (years)	20133	Median (IQR)	80.0 (71.0 to 86.0)	72.2 (59.0 to 83.0)	65.0 (51.0 to 78.0)	72.9 (58.0 to 82.0)
Age (years)	20133	<18	2 (0.0)	84 (1.2)	224 (2.7)	310 (1.5)
		18-39	38 (0.7)	289 (4.3)	740 (9.0)	1067 (5.3)
		40-50	81 (1.6)	458 (6.8)	879 (10.7)	1418 (7.0)
		50-59	274 (5.3)	985 (14.6)	1449 (17.7)	2708 (13.5)
		60-69	662 (12.8)	1164 (17.2)	1470 (17.9)	3296 (16.4)
		70-79	1506 (29.2)	1569 (23.2)	1617 (19.7)	4692 (23.3)
		80+	2602 (50.4)	2220 (32.8)	1820 (22.2)	6642 (33.0)
Any comorbidity	18525	No	559 (11.5)	1201 (21.2)	2401 (30.1)	4161 (22.5)
		Yes	4321 (88.5)	4476 (78.8)	5567 (69.9)	14364 (77.5)
Chronic cardiac disease	17702	No	2706 (57.9)	3643 (68.2)	5884 (76.6)	12233 (69.1)
		Yes	1968 (42.1)	1699 (31.8)	1802 (23.4)	5469 (30.9)
Chronic pulmonary disease	17634	No	3530 (75.6)	4388 (82.6)	6588 (86.0)	14506 (82.3)
		Yes	1137 (24.4)	922 (17.4)	1069 (14.0)	3128 (17.7)
Asthma	17535	No	4041 (87.4)	4521 (85.8)	6433 (84.2)	14995 (85.5)
		Yes	584 (12.6)	749 (14.2)	1207 (15.8)	2540 (14.5)
Smoking	14184	Never Smoked	2105 (57.4)	2684 (64.9)	4179 (65.5)	8968 (63.2)
		Former Smoker	1350 (36.8)	1182 (28.6)	1832 (28.7)	4364 (30.8)
		Yes	214 (5.8)	268 (6.5)	370 (5.8)	852 (6.0)
Chronic kidney disease	17506	No	3537 (76.5)	4389 (83.3)	6750 (88.7)	14676 (83.8)
		Yes	1085 (23.5)	882 (16.7)	863 (11.3)	2830 (16.2)
Diabetes without complications	17599	No	3568 (76.8)	4156 (78.2)	6225 (81.5)	13949 (79.3)
		Yes	1080 (23.2)	1158 (21.8)	1412 (18.5)	3650 (20.7)

Diabetes with complications	17516	No	4231 (91.6)	4838 (91.9)	7148 (93.7)	16217 (92.6)
		Yes	389 (8.4)	429 (8.1)	481 (6.3)	1299 (7.4)
Obesity	16081	No	3768 (90.0)	4328 (89.1)	6300 (89.5)	14396 (89.5)
		Yes	417 (10.0)	531 (10.9)	737 (10.5)	1685 (10.5)
Chronic neurological disorder	17382	No	3917 (85.4)	4586 (87.8)	6908 (91.3)	15411 (88.7)
		Yes	670 (14.6)	639 (12.2)	662 (8.7)	1971 (11.3)
Dementia	17459	No	3626 (78.8)	4458 (84.8)	7015 (92.3)	15099 (86.5)
		Yes	974 (21.2)	797 (15.2)	589 (7.7)	2360 (13.5)
Malignancy	17354	No	3938 (86.5)	4714 (90.1)	6959 (92.0)	15611 (90.0)
		Yes	617 (13.5)	519 (9.9)	607 (8.0)	1743 (10.0)
Moderate/severe liver disease	17360	No	4469 (97.7)	5119 (98.2)	7462 (98.5)	17050 (98.2)
		Yes	105 (2.3)	92 (1.8)	113 (1.5)	310 (1.8)
Mild Liver disease	17331	No	4490 (98.5)	5131 (98.5)	7429 (98.3)	17050 (98.4)
		Yes	70 (1.5)	79 (1.5)	132 (1.7)	281 (1.6)
Chronic hematologic disease	17328	No	4290 (94.1)	5033 (96.7)	7312 (96.7)	16635 (96.0)
		Yes	271 (5.9)	174 (3.3)	248 (3.3)	693 (4.0)
Rheumatologic disorder	17289	No	4019 (88.5)	4701 (90.3)	6873 (91.2)	15593 (90.2)
		Yes	524 (11.5)	505 (9.7)	667 (8.8)	1696 (9.8)
Malnutrition	16695	No	4177 (96.5)	4906 (97.7)	7216 (98.3)	16299 (97.6)
		Yes	152 (3.5)	117 (2.3)	127 (1.7)	396 (2.4)
Prior immunosuppressant medication	18009	Yes	518 (10.9)	432 (8.0)	717 (9.1)	1667 (9.3)
		No	3974 (83.3)	4610 (85.9)	6787 (86.2)	15371 (85.4)
		N/A	277 (5.8)	325 (6.1)	369 (4.7)	971 (5.4)
Prior anti-infective medication	18017	Yes	948 (19.9)	934 (17.4)	1369 (17.4)	3251 (18.0)
		No	3540 (74.3)	4131 (76.9)	6134 (77.8)	13805 (76.6)
		N/A	278 (5.8)	306 (5.7)	377 (4.8)	961 (5.3)
AIDS/HIV	17251	No	4506 (99.5)	5170 (99.6)	7492 (99.5)	17168 (99.5)

		Yes	23 (0.5)	23 (0.4)	37 (0.5)	83 (0.5)

Table E4. Survival analysis. Cox Proportional Hazards analysis incorporating CCG/Health Board (geography) as a random effect (hierarchical multivariable). Discharged patients are not censored and held within the risk set (absorbing state/competing risks analysis). N = 15,194, N events = 3911. HR, hazard ratio. Results are hazard ratio (95% confidence interval, p-value) unless otherwise stated.

		N (%)	HR (univariable)	HR (multivariable)	HR (hierarchical multivariable)
Age on admission (years)	<50	2778 (14.0)	-	-	-
	50-59	2693 (13.6)	2.55 (2.06-3.17, p<0.001)	2.65 (2.08-3.38, p<0.001)	2.63 (2.06-3.35, p<0.001)
	60-69	3253 (16.4)	5.45 (4.48-6.63, p<0.001)	5.01 (4.00-6.27, p<0.001)	4.99 (3.99-6.25, p<0.001)
	70-79	4624 (23.3)	9.76 (8.09-11.77, p<0.001)	8.60 (6.92-10.67, p<0.001)	8.51 (6.85-10.57, p<0.001)
	80+	6524 (32.8)	13.47 (11.20-16.20, p<0.001)	11.44 (9.22-14.20, p<0.001)	11.09 (8.93-13.77, p<0.001)
Sex at Birth	Male	11932 (60.0)	-	-	-
	Female	7940 (40.0)	0.85 (0.81-0.90, p<0.001)	0.80 (0.75-0.86, p<0.001)	0.81 (0.75-0.86, p<0.001)
Chronic cardiac disease	No	12115 (69.2)	-	-	-
	Yes	5400 (30.8)	1.93 (1.82-2.04, p<0.001)	1.16 (1.09-1.24, p<0.001)	1.16 (1.08-1.24, p<0.001)
Chronic pulmonary disease	No	14369 (82.4)	-	-	-
	Yes	3079 (17.6)	1.68 (1.57-1.80, p<0.001)	1.19 (1.10-1.29, p<0.001)	1.17 (1.09-1.27, p<0.001)
Asthma	No	14844 (85.5)	-	-	-
	Yes	2510 (14.5)	0.80 (0.73-0.87, p<0.001)	-	-
Smoking	Never Smoked	8900 (63.3)	-	-	-
	Yes	838 (6.0)	1.09 (0.95-1.26, p=0.225)	-	-
	Former Smoker	4327 (30.8)	1.35 (1.26-1.45, p<0.001)	-	-

Chronic kidney disease	No	14527 (83.9)	-	-	-
	Yes	2792 (16.1)	1.92 (1.79-2.05, p<0.001)	1.26 (1.16-1.36, p<0.001)	1.28 (1.18-1.39, p<0.001)
Diabetes	No	12690 (72.4)	-	-	-
	Yes	4849 (27.6)	1.27 (1.20-1.35, p<0.001)	1.06 (0.99-1.14, p=0.089)	1.06 (0.99-1.14, p=0.087)
Obesity	No	14249 (89.5)	-	-	-
	Yes	1671 (10.5)	0.91 (0.82-1.01, p=0.077)	1.32 (1.19-1.48, p<0.001)	1.33 (1.19-1.49, p<0.001)
Chronic neurological disorder	No	15257 (88.7)	-	-	-
	Yes	1947 (11.3)	1.54 (1.42-1.68, p<0.001)	1.19 (1.08-1.30, p<0.001)	1.17 (1.06-1.29, p=0.001)
Dementia	No	14940 (86.5)	-	-	-
	Yes	2336 (13.5)	2.32 (2.16-2.49, p<0.001)	1.39 (1.28-1.51, p<0.001)	1.40 (1.28-1.52, p<0.001)
Malignancy	No	15461 (90.0)	-	-	-
	Yes	1712 (10.0)	1.56 (1.44-1.70, p<0.001)	1.14 (1.03-1.25, p=0.008)	1.13 (1.02-1.24, p=0.017)
Moderate/severe liver disease	No	16873 (98.2)	-	-	-
	Yes	306 (1.8)	1.43 (1.18-1.74, p<0.001)	1.54 (1.23-1.92, p<0.001)	1.51 (1.21-1.88, p<0.001)
Mild Liver disease	No	16869 (98.4)	-	-	-
	Yes	280 (1.6)	0.91 (0.72-1.16, p=0.458)	-	-
Chronic hematologic disease	No	16465 (96.0)	-	-	-
	Yes	686 (4.0)	1.64 (1.45-1.85, p<0.001)	-	-
Rheumatologic disorder	No	15435 (90.2)	-	-	-

	Yes	1671 (9.8)	1.24 (1.14-1.36, p<0.001)	-	-
Malnutrition	No	16128 (97.6)	-	-	-
	Yes	392 (2.4)	1.71 (1.46-2.02, p<0.001)	-	-
Prior immunosuppressant medication	No	15224 (90.3)	-	-	-
	Yes	1644 (9.7)	1.18 (1.08-1.30, p<0.001)	-	-
Prior anti-infective medication	No	13662 (80.9)	-	-	-
	Yes	3222 (19.1)	1.12 (1.04-1.21, p=0.002)	-	-
AIDS/HIV	No	16989 (99.5)	-	-	-
	Yes	83 (0.5)	0.98 (0.65-1.48, p=0.921)	-	-

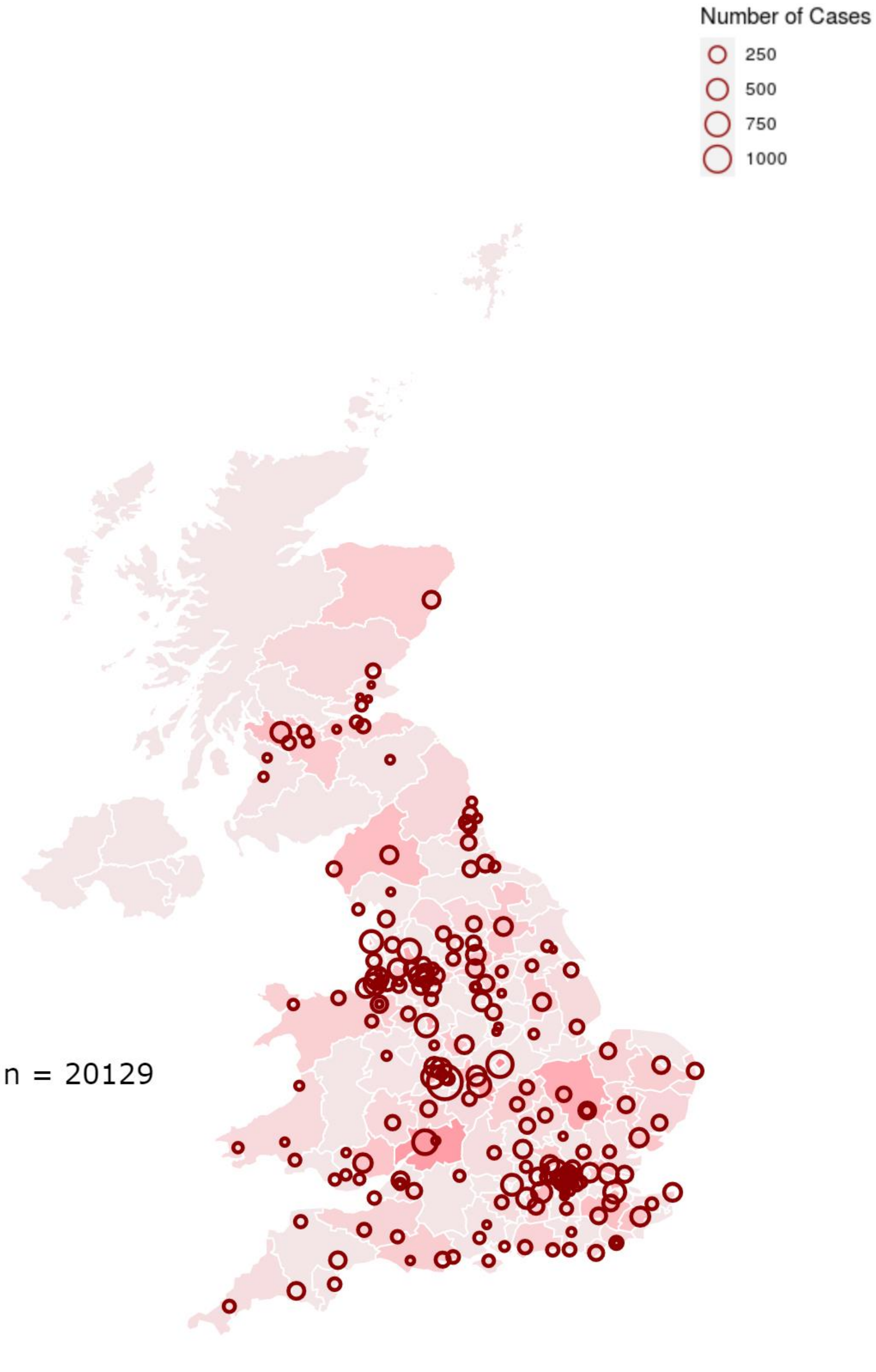

Figure E1: Geographical distribution of patients recruited to ISARIC CCP-UK

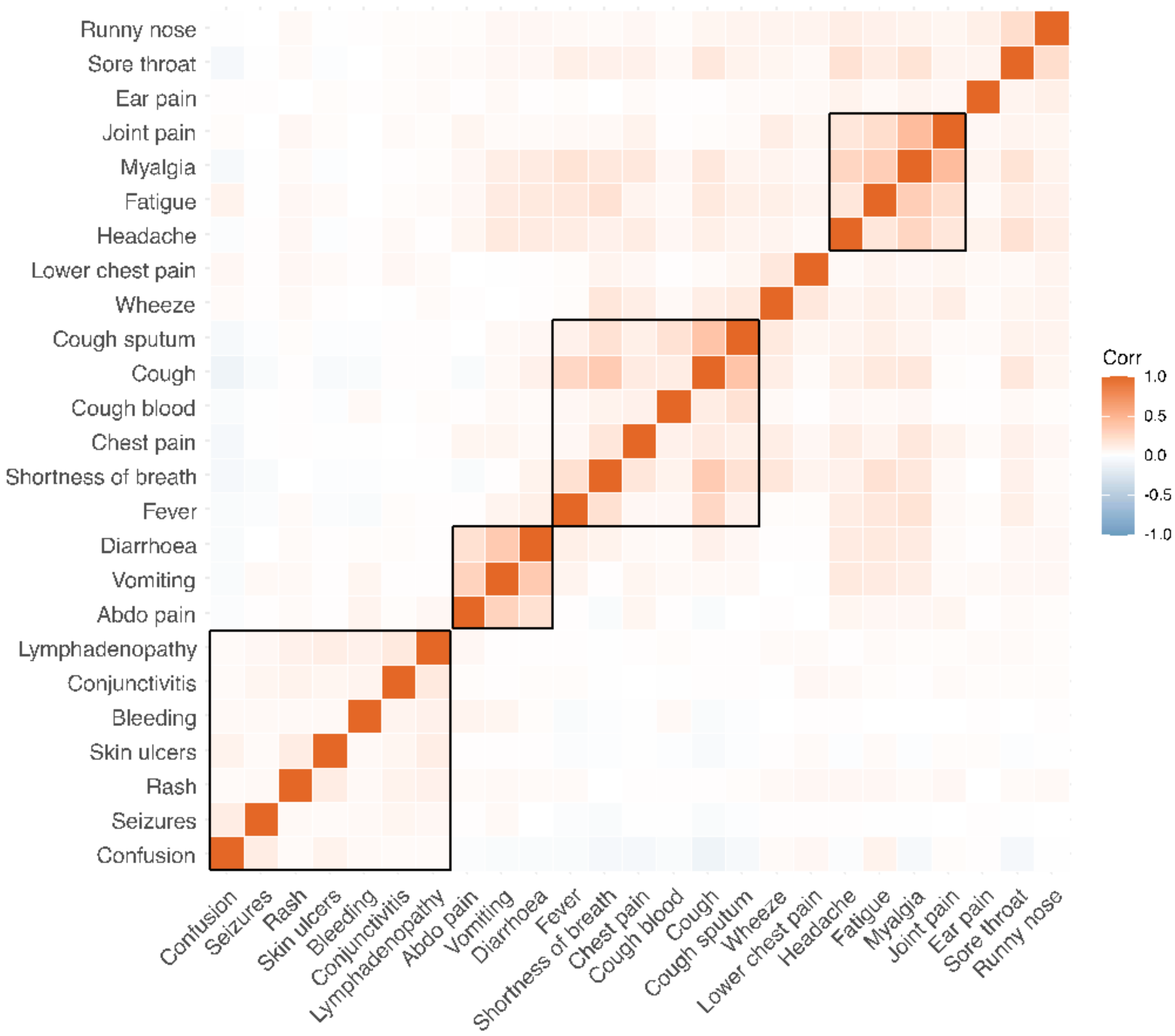

Figure E2. Correlation matrix of symptoms on admission to hospital.

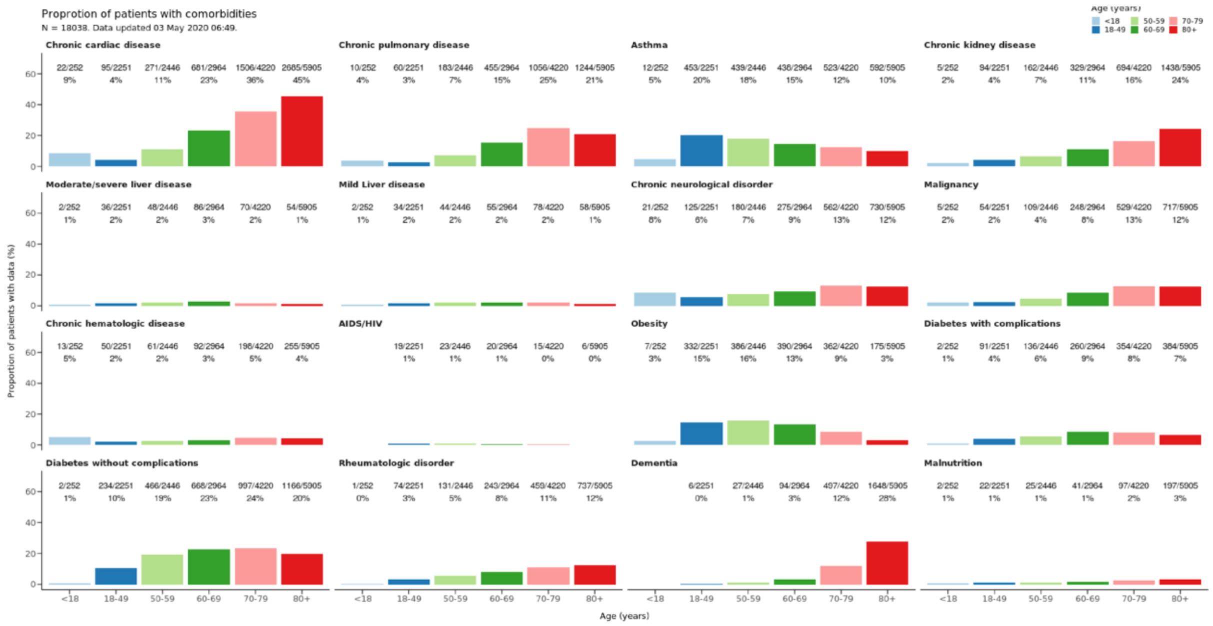

Figure E3. Pattern of comorbidity stratified by age.

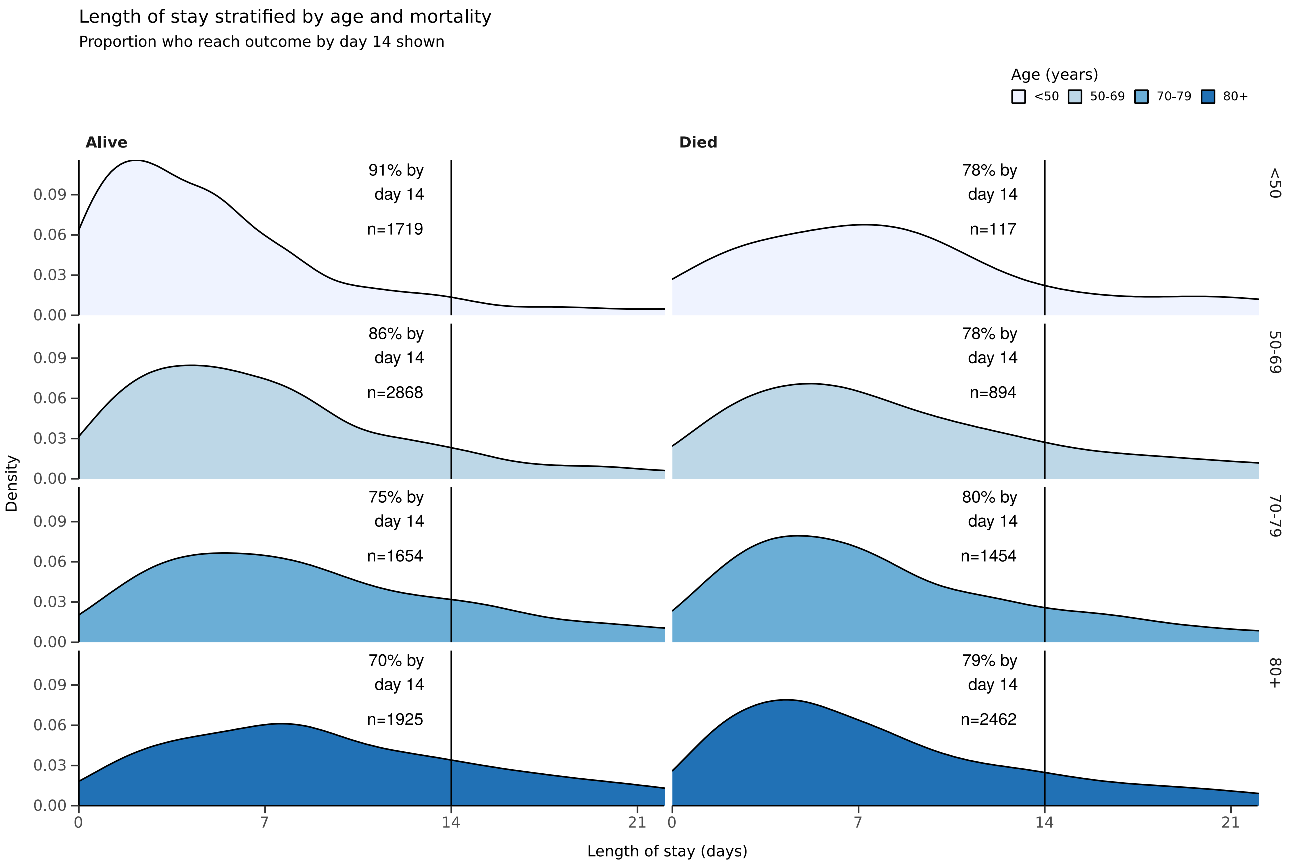

Figure E4. Length of stay stratified by age and mortality.

Table E5. Text box to enable comparison with studies from different locations and health systems

-*What were the criteria for testing for COVID-19, what tests were in use and what was their accuracy, and what if any were the criteria used to diagnose COVID-19?*<br>COVID-19 was diagnosed on viral PCR from nose/throat swab or deep respiratory sampling (endotracheal aspirate or broncho-alveolar lavage),<br>-*Was any rationing in place or were there shortages of staff, beds (especially ICU beds), ventilators, dialysis equipment, etc?*<br>There was no rationing of ICU beds or equipment during this time<br>- *What were criteria for hospital and ICU admission at the time (a general description is fine but if explicit criteria were used please report them)? In particular, please note if certain groups of patients were managed as outpatients rather than being admitted.*<br>There were no explicit criteria for hospital admission after the initial contact tracing and isolation policy ceased on 10th March 2020. Patients admitted to hospital were at the discretion of the assessing clinician.<br>- *What were criteria for intubation and extubation?*<br>In the absence of reliable evidence, opinion has varied considerably between individual ICU clinicians, and units across the country, so that there were no explicit criteria for intubation or extubation.

Table E6: Case definition for COVID-19, “criteria” and “priorities” for testing, by change over time.

**Case definition**	**“Criteria for testing” from 10th January 2020, and “Priorities for testing” from 27 March.**	**Date published**
Interim definition: possible cases<br>If the patient satisfies epidemiological and clinical criteria, they are classified as a possible case.<br>Epidemiological criteria<br>• travel to Wuhan City in the 14 days before the onset of illness, **or**<br>• contact (see definition below) with confirmed cases of WN-CoV<br>Clinical criteria<br>• severe acute respiratory infection requiring admission to hospital with clinical or radiological evidence of pneumonia or acute respiratory distress syndrome, **or**<br>• fever or history of fever (>=38C) and acute respiratory infection (sudden onset of respiratory infection with at least one of: shortness of breath, cough or sore throat)	For the purposes of testing, contact with a case is defined as:<br>• living in the same household<br>OR<br>• direct contact with the case or their body fluids or their laboratory specimens without recommended PPE, or in the same room of a healthcare setting when an aerosol generating procedure is undertaken on the case without recommended PPE<br>• within 2 metres of the case in any other setting not listed above, for any length of time.	10/1/20
Epidemiological criteria<br>In the 14 days before the onset of illness:<br>• travel to Wuhan, Hubei Province, China<br>OR<br>• contact (see definition below) with confirmed cases of WN-CoV<br>Clinical criteria<br>• severe acute respiratory infection requiring admission to hospital with clinical or radiological evidence of pneumonia or acute respiratory distress syndrome<br>OR<br>• acute respiratory infection of any degree of severity (including at least one of: shortness of breath, cough or sore throat)<br>Clinicians should be alert to the possibility of atypical presentations in patients who are immunocompromised.	For the purposes of testing, contact with a case is defined as:<br>• living in the same household<br>OR<br>• direct contact with the case or their body fluids or their laboratory specimens, or in the same room of a healthcare setting when an aerosol generating procedure is undertaken on the case<br>• within 2 metres of the case in any other setting not listed above, for any length of time	16/1/20
Epidemiological criteria<br>In the 14 days before the onset of illness:<br>• travel to mainland China (not including Hong Kong and Macao)<br>OR<br>• contact (see definition below) with confirmed cases of 2019-nCoV<br>Clinical criteria<br>• severe acute respiratory infection requiring admission to hospital with clinical or radiological evidence of pneumonia or acute respiratory distress syndrome<br>OR<br>• acute respiratory infection of any degree of severity (including at least one of: fever, shortness of breath or cough)<br>Clinicians should be alert to the possibility of atypical presentations in patients who are immunocompromised.	For the purposes of testing, contact with a case is defined as:<br>• living in the same household<br>OR<br>• direct contact with the case or their body fluids or their laboratory specimens, or in the same room of a healthcare setting when an aerosol generating procedure is undertaken on the case<br>• within 2 metres of the case in any other setting not listed above, for any length of time	3/2/20

Epidemiological criteria<br>In the 14 days before the onset of illness:<br>• travel to China, Hong Kong, Japan, Macau, Malaysia, Republic of Korea, Singapore, Taiwan, or Thailand<br>OR<br>• contact (see definition below) with confirmed cases of 2019-nCoV<br>Clinical criteria<br>• severe acute respiratory infection requiring admission to hospital with clinical or radiological evidence of pneumonia or acute respiratory distress syndrome<br>OR<br>• acute respiratory infection of any degree of severity, including at least one of shortness of breath or cough (with or without fever)<br>OR<br>• fever with no other symptoms<br>Clinicians should be alert to the possibility of atypical presentations in patients who are immunocompromised.	For the purposes of testing, contact with a case is defined as:<br>• living in the same household<br>OR<br>• direct contact with the case or their body fluids or their laboratory specimens, or in the same room of a healthcare setting when an aerosol generating procedure is undertaken on the case<br>OR<br>• direct or face to face contact with a case, for any length of time<br>OR<br>• being within 2 metres of the case for any other exposure not listed above, for longer than 15 minutes<br>OR<br>• being otherwise advised by a public health agency that contact with a confirmed case has occurred	6/2/20
Epidemiological criteria<br>In the 14 days before the onset of illness:<br>• travel to China, Hong Kong, Japan, Macau, Malaysia, Republic of Korea, Singapore, Taiwan, or Thailand<br>OR<br>• contact (see definition below) with confirmed cases of 2019-nCoV<br>Clinical criteria<br>• severe acute respiratory infection requiring admission to hospital with clinical or radiological evidence of pneumonia or acute respiratory distress syndrome<br>OR<br>• acute respiratory infection of any degree of severity, including at least one of shortness of breath (difficult breathing in children) or cough (with or without fever)<br>OR<br>• fever with no other symptoms<br>Clinicians should be alert to the possibility of atypical presentations in patients who are immunocompromised.	For the purposes of testing, contact with a case is defined as:<br>• living in the same household<br>OR<br>• direct contact with the case or their body fluids or their laboratory specimens, or in the same room of a healthcare setting when an aerosol generating procedure is undertaken on the case<br>OR<br>• direct or face to face contact with a case, for any length of time<br>OR<br>• being within 2 metres of the case for any other exposure not listed above, for longer than 15 minutes<br>OR<br>• being otherwise advised by a public health agency that contact with a confirmed case has occurred	9/2/20

Epidemiological criteria<br>Please note these criteria changed on 7 February 2020, and should be applied prospectively only to those being assessed after this date.<br>In the 14 days before the onset of illness:<br>• travel to China, Hong Kong, Japan, Macau, Malaysia, Republic of Korea, Singapore, Taiwan, or Thailand. This includes transit, for any length of time, in these countries<br>OR<br>• contact (see definition below) with confirmed cases of 2019-nCoV<br>Clinical criteria<br>• severe acute respiratory infection requiring admission to hospital with clinical or radiological evidence of pneumonia or acute respiratory distress syndrome<br>OR<br>• acute respiratory infection of any degree of severity, including at least one of shortness of breath (difficult breathing in children) or cough (with or without fever)<br>OR<br>• fever with no other symptoms<br>Clinicians should be alert to the possibility of atypical presentations in patients who are immunocompromised.	For the purposes of testing, contact with a case is defined as:<br>• living in the same household<br>OR<br>• direct contact with the case or their body fluids or their laboratory specimens, or in the same room of a healthcare setting when an aerosol generating procedure is undertaken on the case without appropriate PPE<br>OR<br>• direct or face to face contact with a case, for any length of time<br>OR<br>• being within 2 metres of the case for any other exposure not listed above, for longer than 15 minutes<br>OR<br>• being otherwise advised by a public health agency that contact with a confirmed case has occurred	11/2/20
Epidemiological criteria<br>Please note these criteria changed on 25 February 2020, and should be applied retrospectively from 19 February.<br>In the 14 days before the onset of illness:<br>• travel to specified countries and areas. This includes transit, for any length of time, in these countries<br>OR<br>• contact (see definition below) with confirmed cases of COVID-19<br>Clinical criteria<br>• severe acute respiratory infection requiring admission to hospital with clinical or radiological evidence of pneumonia or acute respiratory distress syndrome<br>OR<br>• acute respiratory infection of any degree of severity, including at least one of shortness of breath (difficult breathing in children) or cough (with or without fever)<br>OR<br>• fever with no other symptoms	Not changed	25/2/20

Case definitions: possible case, as of 10 March 2020<br>2.1 Patients who meet the following criteria, regardless of epidemiological links<br>• requiring admission to hospital<br>and<br>• have either clinical or radiological evidence of pneumonia<br>or<br>• acute respiratory distress syndrome<br>or<br>• influenza like illness<br>2.2 Patients who meet both epidemiological and clinical case criteria, either well enough to remain in the community or who get admitted to hospital<br>Epidemiological criteria<br>In the 14 days before the onset of illness:<br>• travel to specified countries and areas, including transit for any length of time in these countries or areas<br>or<br>• contact (see definition below) with confirmed cases of COVID-19<br>Clinical criteria<br>• acute respiratory infection of any degree of severity, including at least one of shortness of breath (difficult breathing in children) or cough (with or without fever)<br>or<br>• fever with no other symptoms<br>Clinicians should be alert to the possibility of atypical presentations in patients who are immunocompromised	Not changed	10/3/20
Case definitions: possible case, as of 13 March 2020<br><br>2.1 Patients who meet the following criteria (inpatient definition)<br>• requiring admission to hospital (a hospital practitioner has decided that admission to hospital is required with an expectation that the patient will need to stay at least one night)<br>and<br>• have either clinical or radiological evidence of pneumonia<br>or<br>• acute respiratory distress syndrome<br>or<br>• influenza like illness (fever ≥37.8°C and at least one of the following respiratory symptoms, which must be of acute onset: persistent cough (with or without sputum), hoarseness, nasal discharge or congestion, shortness of breath, sore throat, wheezing, sneezing)<br><br>2.2 Patients who meet the following criteria and are well enough to remain in the community<br>• new continuous cough and/or<br>• high temperature<br>Individuals with cough or fever should now stay at home	Replaced with page 'priority for testing during periods of significant demand'.<br><br>Group 1 (test first): patient requiring critical care for the management of pneumonia, ARDS or influenza like illness (ILI)†, or an alternative indication of severe illness has been provided e.g. severe pneumonia or ARDS<br>Group 2: all other patients requiring admission to hospital* for management of pneumonia, ARDS or ILI<br>Group 3: clusters of disease in residential or care settings e.g. long term care facility, prisons, boarding schools<br><br>† ILI: fever ≥37.8°C and at least one of the following respiratory symptoms, which must be of acute onset: persistent cough (with or without sputum), hoarseness, nasal discharge or congestion, shortness of breath, sore throat, wheezing, sneezing)<br>* admission to hospital: a hospital practitioner has decided that admission to hospital is required with an expectation that the patient will need to stay at least one night	13/3/20

Clinicians should be alert to the possibility of atypical presentations in patients who are immunocompromised.<br>Alternative clinical diagnoses and epidemiological risk factors should be considered		
Case definitions: possible case, as of 13 March 2020<br><br>2.1 Patients who meet the following criteria (inpatient definition)<br>• requiring admission to hospital (a hospital practitioner has decided that admission to hospital is required with an expectation that the patient will need to stay at least one night)<br>and<br>• have either clinical or radiological evidence of pneumonia<br>or<br>• acute respiratory distress syndrome<br>or<br>• influenza like illness (fever ≥37.8°C and at least one of the following respiratory symptoms, which must be of acute onset: persistent cough (with or without sputum), hoarseness, nasal discharge or congestion, shortness of breath, sore throat, wheezing, sneezing)<br>Note: Clinicians should consider testing inpatients with new respiratory symptoms or fever without another cause or worsening of a pre-existing respiratory condition.<br><br>2.2 Patients who meet the following criteria and are well enough to remain in the community<br>• new continuous cough and/or<br>• high temperature<br>Individuals with cough or fever should now stay at home.<br>Clinicians should be alert to the possibility of atypical presentations in patients who are immunocompromised.<br>Alternative clinical diagnoses and epidemiological risk factors should be considered.	--	18/3/20
As above, only change is " Those staying at home are not prioritised for testing."	1. Prioritisation<br>Group 1 (test first): patient requiring critical care for the management of pneumonia, acute respiratory distress syndrome (ARDS) or influenza like illness (ILI)†, or an alternative indication of severe illness has been provided, for example severe pneumonia or ARDS<br>Group 2: all other patients requiring admission to hospital* for management of pneumonia, ARDS or ILI<br>Group 3: clusters of disease in residential or care settings, for example long term care facilities and prisons (usually samples from 5 cases are sufficient in these settings)<br>† ILI: fever ≥37.8°C and at least one of the following respiratory symptoms, which must be of acute onset: persistent cough (with or without sputum), hoarseness, nasal discharge or	27/3/20

	congestion, shortness of breath, sore throat, wheezing, sneezing)<br>*admission to hospital: a hospital practitioner has decided that admission to hospital is required with an expectation that the patient will need to stay at least one night.	
" Those staying at home are not prioritised for testing." text removed	unchanged	7/4/20
Unchanged	Removed priorities for testing	21/4/20

Research

1 Dunning JW, Merson L, Rohde GGU, *et al.* Open source clinical science for emerging infections. Lancet Infect. Dis. 2014;**14**:8–9. doi:10.1016/S1473-3099(13)70327-X

2 Pardinaz-Solis R, Longuere K-S, Moore S, *et al.* ISARIC – enhancing the clinical research response to epidemics. *Int J Infect Dis* 2016;**53**:137. doi:10.1016/j.ijid.2016.11.338
