# Supplementary material for: Features of 20 133 UK patients in hospital with covid-19 using the ISARIC WHO Clinical Characterisation Protocol: prospective observational cohort study
Source: BMJ. 2020 May 22;369:m1985. doi: 10.1136/bmj.m1985 (PMC7243036; doi:10.1136/bmj.m1985)
Supplement: Supplementary file 2 — Web appendix: Supplementary material 2—protocol [file doca057402.ww2.pdf]

ISARIC/WHO Clinical Characterisation Protocol for Severe Emerging Infections in the UK (CCP-UK)

Ethics Ref: 13/SC/0149 (**Oxford C Research Ethics Committee)**

Date and Version No: 17th February 2020 v8.2

UK CRN /CPMS ID 14152 IRAS ID126600

Chief Investigator: Malcolm (Calum) G Semple, PhD BM BCh, Professor of Child Health and Outbreak Medicine, University of Liverpool m.g.semple@liverpool.ac.uk 0151 252 5250

Investigators: Dr J Kenneth Baillie, Senior Clinical Fellow, Roslin Institute, University of Edinburgh; Dr Gail Carson, ISARIC Clinical Coordinator, University of Oxford ; Prof. Peter Openshaw, Professor of Experimental Medicine, Imperial College London; Dr Jake Dunning, Head of Emerging Infections and Zoonoses at the National Infection Service, Public Health England; Laura Merson, University of Oxford; Dr Clark Russell, Clinical Lecturer, University of Edinburgh; Prof. Maria Zambon, Director, Reference Microbiology Services, Public Health England; Dr Meera Chand, Consultant Medical Microbiologist, Reference Microbiology Services, Public Health England; Prof. Richard Tedder, Imperial College London; Prof. Saye Khoo, Institute of Translational Medicine, University of Liverpool; Prof. Peter Horby, Professor of Emerging Infectious Diseases and Global Health, University of Oxford; Dr Lance Turtle, Senior Clinical Lecturer of Infectious Diseases, University of Liverpool; Prof. Tom Solomon, Director of the National Institute for Health Research Health Protection Research Unit in Emerging and Zoonotic Infections, University of Liverpool; Dr Samreen Ijaz, PHE and NHS Blood and Transplant Blood Borne Virus Unit; Dr Tom Fletcher, Senior Clinical Lecturer in Infectious Diseases, Liverpool School of Tropical Medicine; Prof Massimo Palmarini, Director, MRC Centre for Virus Research, Glasgow; Dr Antonia Ho, MRC Centre for Virus Research, Glasgow; Dr Nicholas Price, Consultant in Infectious Diseases, Guy’s and St Thomas’ NHS Foundation Trust.

Sponsor: University of Oxford, Oxford.

Funders: Set up: Li Ka Shing Global Health Foundation, Core: MRC, Wellcome Trust, SPRINT-SARI NIHR Health Protection Research Unit (HPRU) in Respiratory Infection, HPRU in Emerging and Zoonotic Infections

1 Table of Contents

2 Background and Objectives *4*

2.1 Purpose of the Study **4**

2.1.1 Test activation of data collection (internal pilot). 4

2.2 Background Information **5**

2.2.1 Influenza A/H5N1 6

2.2.2 Middle East Respiratory Syndrome coronavirus (MERS-CoV) 6

2.2.3 Influenza A/H7N9 6

2.2.4 Emerging Pathogens causing Severe Acute Respiratory Illness. 6

2.2.5 Emerging or re-emerging pathogens causing viral haemorrhagic fever. 6

2.2.6 Tick borne encephalitis virus (TBEV) and other emerging CNS viruses. 7

2.2.7 Other emerging or re-emerging pathogens of Public Health Interest 7

2.3 Target Audience of this Document **7**

2.4 Source of this Protocol **7**

2.5 Primary Objectives **7**

2.6 Secondary Objectives **8**

2.6.1 Specific objectives of Annual Activation (internal pilot) 8

2.7 Structure of this document: stratified recruitment according to local resource. **9**

2.8 Entry Criteria **10**

2.8.1 Inclusion criteria for SARI patients 10

2.8.2 Inclusion criteria for VHF patients 11

2.8.3 Inclusion criteria for patients with CNS infection 11

2.8.4 Inclusion criteria for patients with infection by pathogens of public health interest 11

2.8.5 Exclusion criteria for all patients 11

3 Study Design *12*

3.1 Sample Size **12**

4 Methods *12*

4.1 Identification of Potential Patients **12**

4.2 Approach to Potential Participants **12**

4.3 Standard of Care **14**

4.4 Data Collection and Sampling for Patients **14**

4.5 Sample and Data Collection Schedules **14**

4.5.1 TIER ZERO schedule 14

4.5.2 TIER 1 schedule 14

4.5.3 TIER 2 schedule 14

4.5.4 For CNS infections only – residual cerebrospinal fluid from clinical sampling 20

4.6 Enrolment Procedures for Patients **21**

4.7 Case Report Form and Patient Numbers **21**

4.8 Follow-Up Procedures for Patients **22**

4.8.1 Large-volume convalescent sampling. 22

4.9 Withdrawal of Patients **23**

5 Specimens and Laboratory Analysis 24
5.1 Specimen Sampling, Storage Procedures and Transport 24
5.2 Additional Data Collection – Pharmacokinetic/Pharmacodynamics Studies 25
5.3 Sample Processing 27
5.4 Use of Stored Samples 28
5.4.1 Research Plan for samples 28
5.5 Future Use of Samples 29
6 Medical Management and Safety Reporting 30
6.1 Medical Management 30
7 Data Management 30
7.1 Data Collection 30
7.2 Data Management 30
7.3 Data Access and Data Sharing 31
7.3.1 Independent data and materials access committee (IDAMAC) 31
7.3.2 Principles of data and materials access 31
7.4 Data Quality 32
7.4.1 Monitoring 32
8 Ethical Considerations 32
8.1 Regulations, Guidelines and Ethical Review 33
8.2 Informed Consent 33
8.3 Alternatives to Participation and Withdrawal 34
8.4 Risks to Participants 34
8.5 Benefits to Participants 35
8.6 Participation in Other Research Studies / Co-enrolment 35
8.7 Confidentiality 36
8.8 Custody of Data and Samples 37
8.9 Additional Ethical Considerations 37
8.10 Insurance 38
8.11 Scientific and Peer Review 38
Material Transfer Agreement 39
8.12 Revision History 42
8.12.1 Changes since v7.3 42
8.12.2 Changes from (Previous version v7.0 25AUG2014) 44
8.13 APPENDIX A 45
8.14 Test Activation Guidance – Internal Pilot Study for maintenance of the UK Clinical Characterisation Protocol 45

To be used in combination with this protocol.. 45

2 Background and Objectives

2.1 Purpose of the Study

This is a standardized generic protocol for the rapid, coordinated clinical investigation of severe or potentially severe acute infections by pathogens of public health interest.

By necessity this protocol is flexible and comprehensive. A supplementary **guidance document** will be used to define the actual sampling frequency and specific samples in use for each site, for a given pathogen.

Patients with a spectrum of emerging and unknown pathogens will be enrolled. This protocol has been designed to maximize the likelihood that data and biological samples are prospectively and systematically collected and shared rapidly in a format that can be easily aggregated, tabulated and analysed across many different settings globally. The protocol is designed to have some level of flexibility in order to ensure the broadest acceptance and has been initiated in response to the recent cases of Middle Eastern Respiratory Syndrome coronavirus (MERS-CoV) in 2012-2013, Influenza H7N9 in 2013, viral haemorrhagic fever (Ebolavirus) in 2014, Monkeypox & MERS-coronavirus in 2018, Tick-borne encephalitis virus (TBEV) in 2019 and Severe Acute Respiratory Syndrome Coronavirus 2 (SARS-CoV-2) in 2020. Information will be circulated by the Investigators and disseminated by the NIHR Clinical Research Network to clarify the eligibility criteria in the event of the emergence of any pathogen of public health interest that is eligible for recruitment to this study.

The study is recognised by the NIHR as being an Urgent Public Health Research study and sits within a portfolio of such studies that will be given priority support in the event of emergence of a pathogen of public health interest (UK CRN ID 14152).

2.1.1 Test activation of data collection (internal pilot).

Recognising the value of maintaining a sleeping study such as this in a state of readiness and to test the readiness of the study, this protocol includes as an activation exercise, an internal pilot for a community-acquired severe acute respiratory infection (SARI). It is intended that this internal pilot will be conducted on an annual basis during the winter season for one week only, subject to funding.

Recent outbreaks of pandemic and zoonotic influenza viruses (H5N1 & H7N9), SARS-CoV, MERS-CoV and Ebola have revealed that there is a significant time lag between the start of a disease outbreak and the availability of the data needed to inform clinical management and public health interventions. There is a lack of information about the epidemiology and management of SARI patients globally, and a recognised need to establish research infrastructure to gather information rapidly during an outbreak of public health interest such as the emergence of a new cause of SARI with epidemic potential.

Eligibility criteria will include people of any ages and sex including pregnant women, with very severe community acquired pneumonia requiring admission to nominated level 3 intensive care units for mechanical ventilation and or ECMO support. This internal pilot is not a separate study in itself, but an important aspect of the study to test the activation that involves collation of data from routine data sources with consent (whether deferred or not) or with proxy assent. It is intended that the 'activation' will be conducted on an annual basis during the winter season for one week only.

The data collated will be also shared on an anonymous basis with an international project on SARI patients - SPRINT-SARI study, led by Dr Srinivas Murthy and Prof. Steven Webb, and coordinated by the ANZIC-RC (Australia) and ISARIC Coordination Centre (Oxford). This study aims to characterise SARI patients globally to better inform management strategies, to improve and inform clinical management of emerging infectious causes of SARI.

The specific guidance for the internal pilot study is included in Appendix A.

2.2 Background Information

Infectious disease is the single biggest cause of death worldwide. New infectious agents, such as the SARS, MERS and other novel coronavirus, novel influenza viruses, viruses causing viral haemorrhagic fever (e.g. Ebola), and viruses that affect the central nervous system (CNS) such as TBEV require investigation to understand pathogen biology and pathogenesis in the host. Even for known infections, resistance to antimicrobial therapies is widespread, and treatments to control potentially deleterious host responses are lacking.

In order to develop a mechanistic understanding of disease processes, such that risk factors for severe illness can be identified and treatments can be developed, it is necessary to understand pathogen characteristics associated with virulence, the replication dynamics and in-host evolution of the pathogen, the dynamics of the host response, the pharmacology of antimicrobial or host-directed therapies, the transmission dynamics, and factors underlying individual susceptibility.

The work proposed here may require sampling that will not immediately benefit the participants. It may also require analysis of the host genome, which may reveal other information about disease susceptibility or other aspects of health status.

This study, while not a study of a medicine, may involve additional procedures (some minimally invasive), the retention of genetic material, collection of personal data and additional follow up. The ISARIC consortium is keen that this protocol serve as a generic template for adoption of this study in other countries and similar studies in the future. ISARIC also intend that this protocol and supporting documents can be used to support or run alongside future intervention studies. For these reasons we aim to fulfil the standards of consent required by Medicines for Human Use (Clinical Trials) Regulations 2004 and NHS NPSA NRES Guidance for Researchers & Reviewers (May 2009).

This protocol as now amended is designed to enrol patients with proven infection by Influenza A/H5N1, A/H7N9, MERS-CoV, SARS-CoV-2, viral haemorrhagic fever, TBEV, any

infection on the PHE/DHSC high consequence infection list (see PHE website), and any other pathogen of public health interest as yet unspecified.

2.2.1 Influenza A/H5N1.

Since 1997, strain A/H5N1 of highly pathogenic avian influenza (HPAI) has emerged as a global zoonosis, and has caused severe sporadic respiratory illness in humans that is associated with an extremely high mortality rate. As of 14 December 2015, the total number of human A(H5N1) cases reported to WHO worldwide is 844; of these 449 have died resulting in a case fatality rate of just under 60%. http://www.who.int/influenza/human_animal_interface/HAI_Risk_Assessment/en/ (accessed 11 January 2016).

2.2.2 Middle East Respiratory Syndrome coronavirus (MERS-CoV).

In September 2012 a novel coronavirus, MERS-CoV, was identified in a patient who died of severe acute respiratory syndrome in June 2012. Since then, large outbreaks have occurred in the Middle East and imported cases have been seen in many countries. As of 7th January 2016, the World Health Organization has been informed of 1,626 confirmed cases, including 586 related deaths. http://www.who.int/csr/don/7-january-2016-mers-oman/en/ (last accessed 11 January 2016).

2.2.3 Influenza A/H7N9.

Two waves of human infection with novel avian influenza A(H7N9) have occurred since March 2013. As of 23 February 2015, 571 laboratory confirmed cases of human infection with influenza A(H7N9) have been reported to WHO, including 212 deaths. The majority of cases presented with respiratory tract infection with progression to severe pneumonia and breathing difficulties. The vast majority of cases have been in mainland China.

2.2.4 Emerging Pathogens causing Severe Acute Respiratory Illness.

Novel pathogens, new strains of existing pathogens, and re-emergence of known dangerous pathogens are a frequent threat to global health. A coordinated clinical research response is critical to identify and describe pathogen and host characteristics to inform a clinical and public health response.

2.2.5 Emerging or re-emerging pathogens causing viral haemorrhagic fever.

Outbreaks of viral haemorrhagic fever (VHF) occur sporadically in Africa, Asia, Europe and South America. The scale and impact of VHF outbreaks vary, but a common feature is for infection to cause significant morbidity and mortality and considerable societal disruption including the provision of healthcare. Global travel means that cases of infection are exported to other countries, with the potential to cause outbreaks outside endemic areas. Important VHF pathogens include Ebola virus, Lassa virus, Crimean-Congo haemorrhagic fever virus, and Marburg virus. In 2014, an unprecedented outbreak of Ebola virus has occurred in humans in West Africa, with large numbers of cases identified across multiple sites in Guinea, Liberia, Sierra Leone, and Nigeria.

2.2.6 Tick borne encephalitis virus (TBEV) and other emerging CNS viruses.

TBEV is a flavivirus spread by ticks to humans in endemic areas across large regions of Europe and Asia. Subtypes of the virus include: European (TBEV-Eu), Far Eastern (TBEV-FE), Siberian (TBEV-Sib), Baikalian (TBEV-Blk), Himalayan (TBEV-Him), and the recently-identified TBEV-UK. TBEV may cause (meningo)encephalitis, with or without myelitis, which can result in death or severe neurological sequelae and often leads to substantial impairment in quality of life. TBEV is a growing public health concern, as the number of human tick-borne encephalitis (TBE) cases continues to increase globally and endemic areas spread northwards and to higher altitudes.

In 2019, ticks carrying TBEV were identified in both Thetford Forest, Norfolk and on the Hampshire/Dorset border; the virus was thought to be imported by bird migration and to be established in the UK. In July 2019, a probable case of serologically-confirmed TBE was reported in an infant in the New Forest, Hampshire. Improved understanding of the epidemiology and clinical features of this disease is essential to inform clinical management and policy, particularly as vaccination against TBEV is available. Other emerging CNS viruses, such as West Nile Virus, Usutu virus, enterovirus D68, Nipah virus and Borna disease virus 1, also cause considerable morbidity and mortality and are a public health priority.

2.2.7 Other emerging or re-emerging pathogens of Public Health Interest

These pathogens will be listed by the investigators taking into consideration position statements issued by of World Health Organisation, Public Health England and other relevant authorities.

2.3 Target Audience of this Document

This document is of primary interest to clinicians (including emergency and critical care providers) and others engaged in identification, triage and treatment of patients with severe acute or potentially severe infections due to the pathogens of interest. Any individuals or members of research units/networks are invited to use this document to facilitate their own studies and contribute data to the centralized database.

2.4 Source of this Protocol

This document is a product of collaboration between the World Health Organization (WHO) and the International Severe Acute Respiratory and Emerging Infections Consortium (ISARIC), and builds on a global consensus on observational research in emerging infections of public health interest.

2.5 Primary Objectives

In potential participants meeting the entry criteria, our primary objectives for each individual pathogen are to:

- Describe the clinical features of the illness or syndrome

- Describe, where appropriate, the response to treatment, including supportive care and novel therapeutics.
- Observe, where appropriate and feasible, pathogen replication, excretion and evolution, within the host, and identify determinants of severity and transmission using high-throughput sequencing of pathogen genomes obtained from respiratory tract, blood, urine, stool, CSF and other samples.
- Characterise, where appropriate and feasible, the host responses to infection and therapy over time, including innate and acquired immune responses, circulating levels of immune signalling molecules and gene expression profiling in peripheral blood.
- Identify host genetic variants associated with disease progression or severity
- Understand transmissibility and the probabilities of different clinical outcomes following exposure and infection

2.6 Secondary Objectives

Secondary objectives are to collect evidence in order to:

- Facilitate effective triage and clinical management of patients with infections relevant to this protocol
- Determine infectivity and appropriate infection control measures of the various pathogens
- Develop clinical guidance documents and offer clinical recommendations to policy makers on the basis of evidence obtained
- Understand the broader epidemiology of an emerging infection through studying potential contacts and asymptomatic individuals

2.6.1 Specific objectives of Annual Activation (internal pilot)

These objectives are only for the annual activation and are complementary to the above objectives:

- To assess the barriers and enablers to being prepared for and conducting research during an outbreak of a pathogen of public health interest and or pandemic at participating sites
- Evaluate the operational characteristics of this study
- Evaluate impact on incidence of alternative case-definitions of SARI
- Incidence of SARI
- Disease severity and risk factors for severe disease due to SARI

- Case Fatality Proportion of SARI
- Duration of ICU/hospital stay due to SARI
- Microbiology of SARI, including variability in testing
- Treatments received during hospitalization for SARI

For further details see appendix A

2.7 Structure of this document: stratified recruitment according to local resource.

The study will be conducted at multiple sites (to be determined by the spread of disease and availability of resources). It is appreciated that settings will vary in terms of clinical infrastructure, resources and capacity. Distinction is made to allow for a resource appropriate implementation of the protocol, and it is understood that data and/or specimen collection may be limited in certain settings. Observational analyses will be stratified according to available samples and data.

In all cases, a case report form (paper CRF or web-based electronic "eCRF") will be completed. In the initial stages of a global public health emergency, outside this research study, the WHO Natural History case report forms will be completed for audit and public health purposes. This extensive case report form contains all of the information needed for this study.

Tiers included in this protocol are:

- **Tier 0 (Clinical data collection only) –** Clinical data will be collected but no additional biological samples will be obtained for research purposes. The minimum clinical data set will summarise the illness episode and outcome, with the option to collect additional detailed clinical data at frequent intervals, according to local resources/needs. Residual diagnostic material will be retained for research purposes.
- **Tier 1 (Single biological sample)** - Clinical samples will be collected on day of recruitment (R - Day 1; ideally at initial presentation to a health care facility). Clinical information will be collected at enrolment and discharge.
- **Tier 2 (Serial biological sampling)** - Clinical samples and data will be collected on day of recruitment (*R* - Day 1; ideally at initial presentation to a health care facility), serial samples will be obtained (*S*), and samples will be obtained during convalescence (*C*) (see below).
- **Tier 3 (Population pharmacokinetics** of antimicrobial/immunomodulatory drugs)

As an outbreak progresses, and more cases occur, it is anticipated that both the research priorities and the local resource availability will change. It is therefore likely that, within a given institution, cases recruited later in an outbreak will be sampled at a lower intensity (see sample priorities, table 2) and may be recruited to a lower tier of the study.

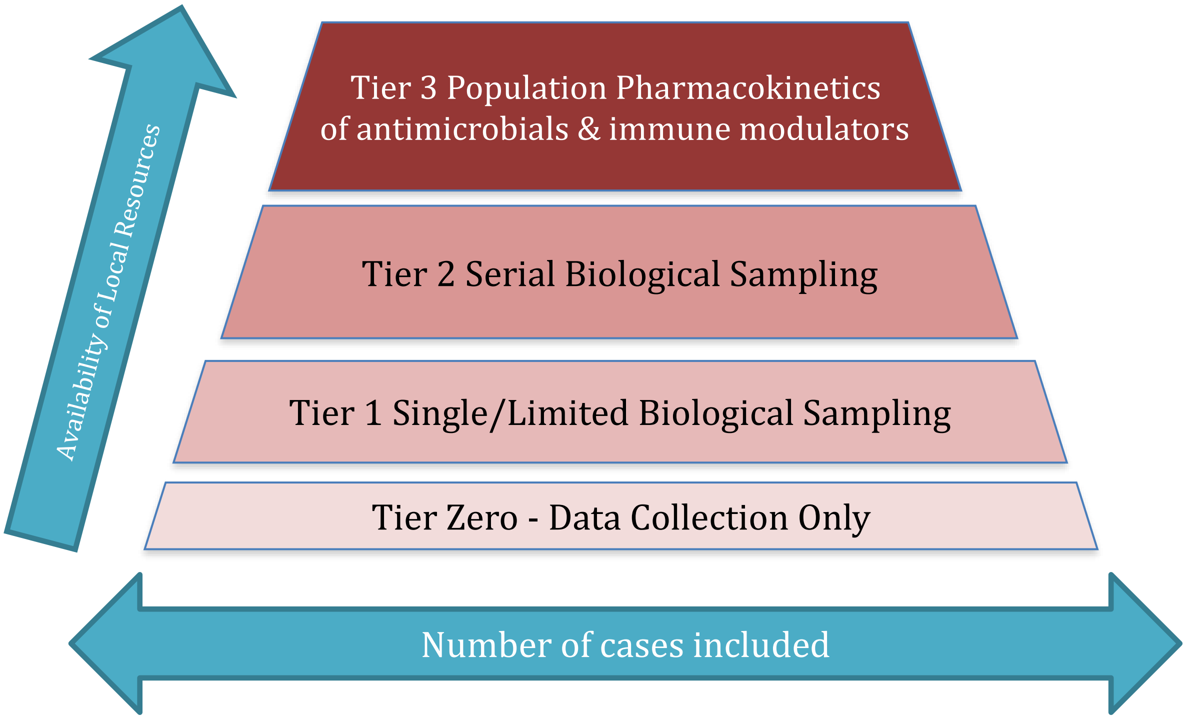

Figure 1. Tiered approach to recruitment in settings with different resources. This information is included to demonstrate the integration of this study with other studies following the same approach in other parts of the world.

The internal pilot study will only collect clinical data and laboratory results for TIER ZERO – data collection only, for one week on annual basis – there will be no additional biological sampling for research purposes. This in turn will inform participating sites about the challenges for collecting data, and ensure that participant sites are better prepared for the activation of the full Clinical Characterisation Protocol in case of an outbreak.

2.8 Entry Criteria

This study will enrol eligible patients (children and adults) with confirmed infection with a pathogen relevant to the study objectives. Recruitment of patients with Day 1 (enrolment) data is the priority.

Daily follow-up and convalescent visits of patients (Table 1 - TIER 2) should proceed according to local resources.

2.8.1 Inclusion criteria for SARI patients

- Acute respiratory illness patients of all ages with a history of fever or measured fever of >38°C and at least one respiratory symptom
- AND high suspicion or confirmed infection with a respiratory pathogen relevant to the objectives of this protocol
- AND admitted to a healthcare facility

Only SARI patients as defined in Appendix A will be included in the annual activation internal pilot study.

2.8.2 Inclusion criteria for VHF patients

- Sudden onset high fever and known contact with a person with suspected or confirmed VHF
- OR sudden onset of fever with at least three of the following symptoms: headache; anorexia; lethargy; aching muscles or joints; breathing difficulties; vomiting; diarrhoea; stomach pain; dysphagia; hiccups
- AND high suspicion or confirmed infection with a VHF pathogen relevant to the objectives of this protocol
- AND admitted to a healthcare facility

2.8.3 Inclusion criteria for patients with CNS infection

- Fever >=38C or history of fever within 30 days in patients of all ages with one of:
    - altered consciousness (including reduced conscious level, confusion, or a change in personality or behaviour)
    - new onset of seizures (excluding simple febrile seizures)
    - new onset focal neurological deficit
    - Electroencephalographic (EEG), neuroimaging or cerebrospinal fluid examination findings indicative of central nervous system infection

• AND high likelihood of infection with a neuroinvasive pathogen of public health interest

• AND admitted to a healthcare facility

OR

• Confirmed infection with a neuroinvasive pathogen of public health interest

• AND admitted to a healthcare facility

2.8.4 Inclusion criteria for patients with infection by pathogens of public health interest

This study will enrol eligible patients with suspected or confirmed infection with a pathogen of public health interest. These pathogens will be listed by the investigators taking into consideration position statements issued by of World Health Organisation, Public Health England and other authorities.

2.8.5 Exclusion criteria for all patients

- Confirmed diagnosis of a pathogen unrelated to the objectives of this study and no indication or likelihood of co-infection with a relevant pathogen.
- Refusal by participant, parent or appropriate representative.

3 Study Design

This protocol is for a prospective observational cohort study.

3.1 Sample Size

This is a descriptive study of a syndrome, which may be caused by a number of different known or poorly understood pathogens. Therefore, the sample size is not prospectively determined. Recruitment of participants will depend on the emergence and spread of the various pathogens and the resources available to the recruitment centres. The sample size will vary for each location but should be as large as feasible and preferably without limit in order to capture as much clinical data as possible early in the outbreak.

This protocol will be opened at sites with capacity and capability to recruit to any tier of study intensity. The study will hibernate in the absence of any relevant cases. The study is exempt from NIHR high-level recruitment performance targets. The study has no set end date.

4 Methods

4.1 Identification of Potential Patients

Approval of the responsible ethics committee and institutional authority will be obtained before patients are recruited at any site. In the UK REC approval has been given for England and Wales (**Oxford C Research Ethics Committee** 13/SC/0149). Local R&D approvals were required from all Acute NHS Trust in England through an expedited process driven forward at the direction of the Chief Medical Officer for England in October 2013 and managed by the Manchester hub of the NIHR Comprehensive Research Network. This protocol is currently under review (February 2020) in Scotland by the Scotland A REC and ethical approval is being sought for Northern Ireland via the HSC RECs.

In hospital, potential participants will be identified through hospital workers upon presentation at recruiting sites and through public health agencies. When resources limit the number of patients enrolled to less than the number of patients presenting, sites should establish procedures to minimize bias in the selection of participants.

4.2 Approach to Potential Participants

Tier Zero activity, which involves data collection only, requires collection of limited clinical data from the routine health record in a form that does not identify the patient. This does not require consent. This is because the patient is not identifiable and the data is collected by a health care professional who has access to this information by virtue of their clinical role.

Tier One and Two

Patients will only be considered for enrolment if appropriate infection control and prevention measures are in place and can be maintained.

When it has been decided that biological sampling can be performed safely and appropriate consent has been obtained, samples taken early may be most useful for identification or evaluation of risk factors for disease progression at a clinically-relevant decision point. Therefore it is desirable to begin sampling as early as possible during a patient's illness.

Where adult patients lack capacity to consent to this Clinical Characterisation Protocol, an appropriate consultee will be approached by staff trained in consent procedures that protect the rights of the patient, and adhere to the ethical principles within the Declaration of Helsinki. Staff will explain the details of the study to the consultee and allow them time to discuss and ask questions. The staff will review the informed consent form with the person giving consent and endeavour to ensure understanding of the contents, including study procedures, risks, benefits, the right to withdraw and alternatives to participation. Participants who agree to participate (or their parent/guardian or consultee who declares their wishes to do so) will be asked to sign and date an informed consent form or consultee declaration form. If the patient is a child, the person with parental responsibility and the child, if competent, should both provide consent/ assent. Summary information sheets and consent forms have been produced to reduce the initial burden on patients, parents/guardians and consultees and these summary information sheets will be used as the basis for the consent discussion. The full study information sheets for adult patients, parents/guardians, and consultees will be provided for their information, subsequent to the initial consent discussion.

In view of the importance of early samples, participants or their parent/guardian/consultee will be permitted to consent/give advice and begin to participate in the study immediately if they wish to do so. Those who prefer more time to consider participation will be approached again after an agreed time, normally one day, to discuss further.

An outbreak involving a pathogen of public health interest or pandemic is an emergency situation. Patients who are incapable of giving consent in emergency situations are an exception to the general rule of informed consent in clinical research. This is clearly acknowledged in the Declaration of Helsinki (2008). The process of consent will comply in England and Wales with the Mental Capacity Act 2005 (MCA 2005); in Scotland, with Section 51 of the Adults with Incapacity (Scotland) Act 2000; and in Northern Ireland with the 2016 Mental capacity (Northern Ireland) Act.

For studies that collect or collate only anonymised data that is normally collected, as part of routine care consent may not be required. The internal pilot study will only collate data that is being recorded or generated as part of routine clinical care (e.g. microbiology results). We will seek consent, be it deferred, proxy or assent, in order to test the processes within the overarching Clinical Characterisation Protocol, which include obtaining consent.

All patients will be treated according to clinical requirements regardless of their participation in the study.

4.3 Standard of Care

Provision of care will vary by site and by treating physician. It is not possible to define a single standard of care and therefore to define what samples will be taken as a part of medical management and when. Participants in this study may have samples taken in addition to those required for medical management. The results of tests performed on research samples are unlikely to benefit the health of the participants.

4.4 Data Collection and Sampling for Patients

Samples required for medical management will at all times have priority over samples taken for research tests. Aliquots or samples for research purposes should never compromise the quality or quantity of samples required for medical management. Wherever practical, taking research samples should be timed to coincide with clinical sampling. The research team will be responsible for sharing the sampling protocol with health care workers supporting patient management in order to minimise disruption to routine care and avoid unnecessary procedures.

Some samples should be processed and stored at -80°C. We recognise that -80°C storage is not available at all sites. In this case please store at coldest available temperature and at least -20°C.

For patients with VHF such as Ebola virus, the biological sampling will at times be limited to extra volumes of blood taken at times to coincide when blood is being taken for clinical purposes and then only at the discretion of the clinical team.

4.5 Sample and Data Collection Schedules

4.5.1 TIER ZERO schedule

Collect data per CRF. There must be no biological sampling for research purposes.

4.5.2 TIER 1 schedule

A single sample set is obtained at, or as soon as practical after, recruitment. Collect data in CRF.

4.5.3 TIER 2 schedule

In TIER 2, three acute serial sample sets and one convalescent set are obtained. Collect data in CRF.

Table 1. TIER2 sampling schedule

	Recruitment	Week 1						Week 2							Convalescent sample
Day	1	2	3	4	5	6	7	8	9	10	11	12	13	14	>28 days after hospital discharge
**Sample set**	**R**		**S**						**S**						**C**
Priority	1		2						3						4

In the event that local resource limitations require sampling frequency to decrease, samples will be prioritised as shown (1=highest priority). Sample sets refer to the tables below: R – Recruitment samples; S- Serial samples; C – convalescent samples.

Table 2. Core sample set – to be obtained at all sampling points

<table>
<tr><th>CORE SAMPLE SET</th><th>Processing/ storage</th><th>Purpose</th></tr>
<tr><td>Pathogen samples:
• Respiratory samples:
○ nasal SAM strip,
○ throat swab in virus transport medium,
○ endotracheal aspirate if intubated,
○ where resources permit, in infants/children who cannot take SAM strip, nasopharyngeal aspirate OR flocked nose swab in virus transport medium;
• Urine (up to 10ml);
• Stool (up to 10ml) or rectal swab;
• samples from infected sites/sores.
• Also store any residual from samples taken for clinical care.</td><td>Do not process at site.
Keep double-bagged.
Store at -80°C*</td><td>Pathogen studies to reveal changes in pathogen during infection and during spread between individuals, detect development of resistance.</td></tr>
<tr><td>Oral fluid (Crevicular fluid)</td><td>Store at -80°C*</td><td>Non-invasive determination of humoral immune response</td></tr>
<tr><td rowspan="2">Blood sample in serum (clotted) tube</td><td rowspan="2">Serum
(3 aliquots -80°C*)</td><td>Mediators/biomarkers</td></tr>
<tr><td>Serology</td></tr>
<tr><td rowspan="2">Blood sample in EDTA tube
(Note – large volume at recruitment)</td><td rowspan="2">Plasma
(3 aliquots -80°C*)</td><td>Mediators/ metabolites/ biomarkers</td></tr>
<tr><td>Detect RNA/DNA from pathogens.</td></tr>
</table>

	Cell fraction (1 aliquot -80°C*)	Extract host DNA for genomic studies
		RNA/DNA from pathogen, cellular immunology.
**Blood** sample in blood RNA tube Tempus™ (or PAXgene®)	Freeze at -20°C; transfer to -80°C after 24h where possible	Microarray/RNA sequencing pathogen & host transcriptome

*freeze at -80°C where possible, or at least at -20°C. If necessary (eg. weekends/public holidays) store in refrigerator until processing. For details, see Sample processing section, below.

Table 3. Blood sample volumes

	**Samples at recruitment (R)**	**Serial samples (S)**	**Convalescent samples (C)**	**Total volume of blood**
>40kg	**9ml** (3x3ml) EDTA blood<br>**3ml** blood in serum(clotted) tube<br>**3ml** blood in blood RNA tube<br>Research pathogen samples	**3ml** EDTA blood<br>**3ml** blood in serum(clotted) tube<br>**3ml** blood in blood RNA tube<br>Research pathogen samples	**3ml** EDTA blood<br>**3ml** blood in serum(clotted) tube<br>**3ml** blood in blood RNA tube<br>Research pathogen samples	Maximum any day: 15ml (0.38ml/kg)<br>Maximum any 4 weeks: 96ml (max 2.4ml/kg)
20 to 40kg	**6ml** (3x2ml) EDTA blood<br>**3ml** blood in serum(clotted) tube<br>**3ml** blood in blood RNA tube<br>Research pathogen samples	**1ml** EDTA blood<br>**2ml** blood in blood RNA tube<br>Research pathogen samples	**1ml** EDTA blood<br>**3ml** blood in serum(clotted) tube<br>**2ml** blood in blood RNA tube<br>Research pathogen samples	Maximum any day: 12ml (0.6ml/kg)<br>Maximum any 4 weeks: 42ml (max 2.1ml/kg)
10 to 20kg	**2ml** (2x1ml) EDTA blood<br>**2ml** blood in serum(clotted) tube<br>**2ml** blood in blood RNA tube<br>Research pathogen samples	**1ml** EDTA blood<br>**1ml** blood in blood RNA tube<br>Research pathogen samples	**1ml** EDTA blood<br>**1ml** blood in serum(clotted) tube<br>**1ml** blood in blood RNA tube<br>Research pathogen samples	Maximum any day: 6ml (0.6ml/kg)<br>Maximum any 4 weeks: 23.6ml (max 2.36ml/kg)
4 to 10kg	**1ml** EDTA blood<br>**0.5ml** blood in serum(clotted) tube<br>**0.5 ml** blood in blood RNA tube<br>Research pathogen samples	**1ml** EDTA blood<br>Research pathogen samples	**1ml** EDTA blood<br>**1ml** blood in serum(clotted) tube<br>Research pathogen samples	Maximum any day: 2ml (0.5ml/kg)<br>Maximum any 4 weeks: 9.4ml (max 2.35ml/kg)
< 4kg	**0.5ml** EDTA blood<br>**0.1ml** blood in serum(clotted) tube<br>**0.1ml** blood in blood RNA tube<br>Research pathogen samples	**0.2m**l EDTA blood<br>Research pathogen samples	**0.2ml** EDTA blood<br>**0.2ml** blood in serum(clotted) tube<br>Research pathogen samples	Maximum any day: 0.8ml (~0.27ml/kg)<br>Maximum any 4 weeks: 2.4ml (max 2.4ml/kg)

Table 4. Data collection and documentation.

	**Samples/documents**
**R – RECRUITMENT SAMPLE SET**	Consent form<br>OBTAIN CORE SAMPLE SET<br>Initiate CCP CASE REPORT FORM
**S- SERIAL SAMPLE SET**	OBTAIN CORE SAMPLE SET<br>Update CCP CASE REPORT FORM
**On hospital discharge**	Update CCP CASE REPORT FORM<br>Plan convalescent visit
**C – CONVALESCENT SAMPLE SET**	OBTAIN CORE SAMPLE SET<br>Update CCP CASE REPORT FORM

Table 5. Optional substudies

<table>
<tr><th>OPTIONAL SUBSTUDIES</th><th colspan="2">SAMPLE SET AND SAMPLE</th><th>Processing/storage</th><th>PURPOSE</th></tr>
<tr><td colspan="5">(Each substudy will only operate in a small minority of sites. Any site participating in a substudy will alert staff to this fact in the TIER RECORD FORM at the front of the site file)</td></tr>
<tr><td rowspan="7">PHARMACOKINETICS</td><td colspan="2">ADD TO ALL SAMPLE SETS (R, S, and C)
Blood sample in EDTA or fluoride oxalate tubes.</td><td rowspan="7">Plasma
(2 aliquots -80°C*)</td><td rowspan="7">Test for drug levels. Store aliquot for other studies.</td></tr>
<tr><td colspan="2">Volumes</td></tr>
<tr><td>>40kg:</td><td>3ml</td></tr>
<tr><td>20 to 40kg:</td><td>0.5ml</td></tr>
<tr><td>10 to 20kg:</td><td>0.2ml</td></tr>
<tr><td>4 to 10kg:</td><td>0.2ml</td></tr>
<tr><td>< 4kg:</td><td>0.2ml</td></tr>
<tr><td rowspan="7">CELLULAR IMMUNOLOGY
(if patient not included in pharmacokinetic study)</td><td colspan="2">ADD TO ALL SAMPLE SETS (R, S, and C)
Blood sample in EDTA</td><td rowspan="7">Extract and store peripheral blood mononuclear cells</td><td rowspan="7">Study host immune response, generate monoclonal antibodies.</td></tr>
<tr><td colspan="2">Volumes</td></tr>
<tr><td>>40kg:</td><td>3ml</td></tr>
<tr><td>20 to 40kg:</td><td>0.5ml</td></tr>
<tr><td>10 to 20kg:</td><td>0.2ml</td></tr>
<tr><td>4 to 10kg:</td><td>0.2ml</td></tr>
<tr><td>< 4kg:</td><td>0.2ml</td></tr>
<tr><td>ENVIRONMENTAL TRANSMISSION</td><td colspan="2">Specimen collection devices placed in vicinity of patient</td><td></td><td></td></tr>
<tr><td>LARGE-VOLUME CONVALESCENT SAMPLING
(in a small number of selected patients in specific institutions)</td><td colspan="2">Up to 240mls of blood in fully recovered patients</td><td>Separation and storage of plasma.
Extraction of peripheral blood mononuclear cells (PBMCs)</td><td>Serology tests, development of products including international standards, cellular immunology, generation of monoclonal antibodies for research, diagnostic and therapeutic use</td></tr>
</table>

4.5.4 For CNS infections only – residual cerebrospinal fluid from clinical sampling

Table 6. Cerebrospinal fluid sampling

<table>
<tr><th>Sample</th><th>Processing</th><th>Purpose</th></tr>
<tr><td rowspan="3">Additional cerebrospinal fluid sample during clinical lumbar puncture

If after recruitment a lumbar puncture is clinically indicated, an additional sample of up to 5mls (table 4) will be collected in a universal sterile tube, provided it is deemed appropriate by the supervising clinician.

Any residual CSF from samples taken as part of routine clinical care will be collected and stored if available.</td><td rowspan="3">3 aliquots stored at -80°C, according to relevant PHE guidance.</td><td>Extract RNA/DNA from causative pathogens and other circulating pathogens for molecular testing, genomic studies and virus isolation</td></tr>
<tr><td>Perform serological testing for pathogen-specific antibodies</td></tr>
<tr><td>Test for mediators, metabolites and potential biomarkers</td></tr>
</table>

Table 7. Estimates of CSF production rate, total CSF volume and the safe recommended CSF volume taken at lumbar puncture for different age groups. Taken from the British Infection Society guidelines for the diagnosis and treatment of tuberculosis of the central nervous system.

Age	Mean CSF production rate (ml/h)	Total CSF Volume (mls)	Safe CSF volume to take at LP (mls)
Adult	22	150-170	Maximum: 15-17
Adolescent	18	120-170	Maximum: 12-17
Young child	12	100-150	Maximum: 10-15
Infant	10	60-90	Maximum: 6-9
Term Neonate	1	20-40	Maximum: 2-4

4.6 Enrolment Procedures for Patients

Patients who meet the inclusion/exclusion criteria and who have given informed consent to participate directly, or have been consented by a parent/guardian or whose wishes have been declared by a consultee, or be it deferred, proxy or assent, will be enrolled to the study. With due consideration to the circumstances of admission to a high level isolation unit a summary information sheet will be used as the basis of the consent discussion and a full study information sheet will be given subsequent to the consent discussion.

All patients will have clinical information collected either directly through examination including a review of medical, contact and travel history, or from available medical notes. Information will be recorded in the case report form.

At enrolment, sites with available resources will obtain a core sample set (see above). The day of initial sample collection will be counted as Day 1. All study days will be counted from this point forward. Clinical information will also be collected on discharge.

During the one week of test activation for the internal pilot study, we will collect only anonymous data from patients that meet the selection criteria defined in Appendix A.

4.7 Case Report Form and Patient Numbers

Case Report Forms (CRFs), based on the WHO Natural History Protocol Case Report Forms, will be used to collect data at enrolment to this study. For this protocol, there are three sets of CRFs – SARI, VHF, and CNS. This can be completed after site registration at https://redcap.medsci.ox.ac.uk/ .

Participant numbers consist of a 5-characterdigit ODS site code and a 4-digit patient number. Your ODS (aka CMPS) code is known by your local R&D Office . Local investigators should be assigned patient numbers sequentially for each site beginning with 0001. In the case of a single site, recruiting patients on different wards, or where it is otherwise difficult to assign sequential numbers, it is acceptable to assign numbers in blocks. E.g. Outpatient ward will assign numbers from 0001 onwards. In-patient ward will assign numbers from 5001 onwards. Please enter the patient identification code at the top of each and every sheet. For settings or circumstances in which resources are constrained, an abbreviated core case report form is provided –Rapid CRF.

For the internal pilot study, TIER ZERO data collection only will be used at each site using the CRF. The eCRF is available by registering on the data management system at https://redcap.medsci.ox.ac.uk/ by contacting ncov@isaric.org . For the full study and internal pilot, each patient will be identified via a unique patient number consist 5-character site code (your site ODS), and each patient will be assigned a 4-digit sequential patient code.

4.8 Follow-Up Procedures for Patients

Follow-up procedures will be undertaken only when resources allow according to TIER 2 sampling outlined in Table 1. Follow-up procedures will only be undertaken if appropriate biological safety measures can be maintained. Sites unable to perform daily follow-up as described below may reduce the frequency of follow-up procedures or exclude follow-up if necessary.

Regular clinical assessment and sampling will follow local guidelines. All patients will have further clinical information recorded in the case report form to record events and treatment experienced during hospitalization and outcome. Some of the samples described below will coincide with clinical management. The number of these will depend on the applicable care guidelines, the treating physician and the health of the patient.

Resolution of acute illness is defined as: Clearance of pathogen from appropriate samples, return of systemic inflammatory response to considered 'normal' values and one of: 1) recovery from organ failure(s)/need for organ support, 2) resolution of the presenting complaint(s), 3) return to life-style prior to illness.

Procedure for additional sampling for pharmacokinetic/ pharmacodynamics studies. [Where a pharmacokinetic study is run concurrently with this protocol] Up to 3 additional samples may be obtained at intervals spread throughout the dosing schedule (ideally including one sample immediately before a dose) of the drug being studied. The spread of the samples can be determined on a case-by-case basis to fit in with clinical care; provided the precise times of administration and the precise time of blood sampling are recorded, samples taken at any time will be of use for analysis using population pharmacokinetic methods.

Samples will be taken in conjunction with those required for clinical care in order to minimize research-specific intervention. Samples taken outside of the scheduled days can be used for study testing and should be recorded with the accurate sampling date.

A sputum sample will be collected when a productive cough is present, and the patient is able to produce one.

Infection site samples are samples of tissue or fluid or swabs taken from infected sites such as an inflamed oropharynx or inflamed conjunctiva.

Residual volumes of all other samples taken for clinical care will be stored.

4.8.1 Large-volume convalescent sampling.

In a small number of patients (likely to be less than 10 patients for each emerging infection) there is a need for additional sampling after recovery from acute illness to enable generation of serological tests, setting of reference standards for serology, extraction and culture of peripheral blood mononuclear cells (PBMCs) for cellular immunology studies, and generation of monoclonal antibodies for research, diagnostic and

therapeutic use. These studies are often extremely valuable in the global response to a new pathogen.

Immune cells, including monocytes, monocyte-derived macrophages, neutrophils and lymphocytes will be isolated from peripheral blood and studied immediately or following culture. Gene expression, protein synthesis and degradation, cytokine release and other functional studies will be measured in immune cells from cases and age- and sex- matched controls. Cells will be stored for future use and may be used in the generation of commercial products.

Patients who participated, with appropriate consent, in this study may be invited to provide additional samples under separate consent for this part of the study. All blood samples will be obtained by an experienced phlebotomist. Participants will be fully recovered, otherwise healthy individuals with no contraindications to blood donation, including:

- Infection with any blood borne diseases (e.g. HIV, Hepatitis B or Hepatitis C)
- Previous or current intravenous drug abuse
- Current anaemia
- Blood clotting disorders
- Current anticoagulant (blood thinning) drug therapy
- History of donations to the blood transfusion service (or any other donation) within the last 12 weeks.

Depending on the participant's weight, the following maximum volumes of blood will be obtained:

- >40kg: 240mls (6.0mls/kg)
- 20-40kg: 80mls (4.0mls/kg)

4.9 Withdrawal of Patients

Patients enrolled to the study whose illness is subsequently confirmed to be the result of infection with a pathogen which is not relevant to the objectives of this study, and who have no indication or likelihood of co-infection with a relevant pathogen, will be withdrawn. No further follow-up will be conducted.

Patient autonomy to withdraw from the study at any time must be respected.

5 Specimens and Laboratory Analysis

5.1 Specimen Sampling, Storage Procedures and Transport

Appropriate selection and timely collection of high-quality specimens, proper storage procedures and comprehensive diagnostic testing will ensure the quality of data.

Public Health England (PHE) is a co-applicant on this study and has made the following recommendation: The study requires collection of research samples in addition to samples used for clinical and public health management. Well-established hospital protocols may be used to collect samples, however guidance on the collection of samples from SARI patients is also found in the WHO draft document "Collecting, preserving and shipping specimens for the diagnosis of influenza virus infection" (2011).

Guidance on the collection of specimens from VHF patients can be found in the WHO document “Interim infection prevention and control guidance for care of patients with suspected or confirmed Filovirus haemorrhagic fever in health-care settings, with focus on Ebola” (2014).

It is expected that BSL3 SARI and CNS pathogens will be sent to PHE Colindale or the local HPRU in Liverpool (University of Liverpool) or London (Imperial College London). It is expected that BSL4 VH pathogen samples will be sent to PHE Porton Down however BSL4 clinical and research capacity has also been commissioned at the Royal Liverpool Hospital and the HPRU in Liverpool University.

In dealing with novel pathogens where little is known about transmissibility and/or virulence, great care must be exercised to ensure the safety of hospital staff and other patients. Strict adherence to collection protocols, biosafety and adequate personal protective equipment (PPE) is essential.

Trusts should follow the usual sources of advice regarding laboratory containment of the pathogen. In an emerging infection this may include information from ACDP and PHE, which would support a local, risk assessment and SOP covering the handling of samples from the affected patient.

Novel respiratory infections or neurological infections may be classified into HG2, HG3 or HG4, as is the case for the currently included pathogens, novel coronavirus MERS-CoV, influenza A/H7N9, A/H5N1, viral hemorrhagic fever ebolavirus and known subtypes of TBEV, including European (TBEV-Eu), Far Eastern (TBEV-FE), Siberian (TBEV-Sib), Baikalian (TBEV-Blk), Himalayan (TBEV-Him) and TBEV-UK.

Other emerging or remerging pathogens may be classified as requiring BSL2, BSL3 or BSL4 safety management and guidelines should be consulted as per hospital protocol. In addition, an emergent agent may also be risk assessed as posing a threat to animal health, and may be regulated under the specified animal pathogens order as well. Laboratories planning to participate in the study should consider how they would fulfil a requirement to handle research samples in addition to clinical samples.

All samples collected must be labelled as per hospital procedure with appropriate identification (full patient identifiers) and hazard labelling according to local policy and ideally marked with a freeze-proof research label or with a solvent resistant marker. These samples that retain full identifiers will be stored within a home office approved high security facility. Samples collected in the household will be labelled with pseudoanonymised patient study codes. Samples will be processed as per the table below. Testing that cannot be done in country will be exported with the permission of the patient/parent/guardian/consultee. Any samples sent to external research laboratories (outside the Liverpool and Imperial HPRUs, PHE Colindale and PHE Porton) will be anonymised with unique coded identifiers to protect the identity of the patient at the site level at the point of enrolment. When required, national guidance will be adhered to for the transport of specimens.

Clinical samples will be labelled with standard hospital information, including the sample date and sent with the standard lab request forms.

Research samples for SARI cases in England and Wales will be transported to the Health Protection Research Unit in Liverpool. In Scotland samples will be transported to the MRC Virology Unit in Glasgow. VHF samples will be sent to the PHE laboratories to be agreed at that time. The study team will organise couriers.

Patient data submitted on the CRF or eCRF must be anonymised using the following procedure. Participant numbers consist of a common 5-character ODS (CMPS) site code and a unique 4-digit patient number. Your R&D Office will know your ODS site code. Your site must maintain a recruitment log linking consent to Participant ID numbers.

Patient numbers should be assigned sequentially by each site beginning with 0001. In the case of a single site recruiting patients on different wards, or where it is otherwise difficult to assign sequential numbers, it is acceptable to assign numbers in blocks. E.g. Outpatient ward will assign numbers from 0001 onwards. In-patient ward will assign numbers from 5001 onwards. Please enter the patient identification code at the top of each and every CRF sheet. Patient numbers and full identifiers must be shared with the ISARIC secretariat. Patient identifiers will not be shared with research institutes.

A unique alphanumeric code for patient samples will be given to each patient at PHE laboratory or the Health Protection Research Units in Liverpool and the only link between the patient's identifying data and this code will be held securely and shared only with the study administrators. The study administrators will link patient data numbers with sample identifiers. The patient identifiers will not be shared with any party.

Residual volumes available after clinical and research testing is complete will be retained for future ethically approved research and this may include commercial purposes.

5.2 Additional Data Collection – Pharmacokinetic/Pharmacodynamics Studies

Where local resources allow, additional information and samples will be sought during treatment with antimicrobial or immunomodulatory therapies in order to investigate the

relationship between dose and plasma drug concentrations, to determine the variability in pharmacokinetics in patients receiving these drugs, and to identify the key pharmacokinetic drivers of pharmacodynamic outcomes (measured using pathogen load, inflammatory markers, illness severity scores or drug toxicity). This information will be collected on the pharmacokinetics record form, and includes both the precise (to the minute) times of drug administration and the precise time of blood sampling.

Samples obtained will be split as required for pharmacokinetic/pharmacodynamic analysis of each antimicrobial or immunomodulatory therapy prescribed; the volume of blood to be drawn will not increase.

5.3 Sample Processing

Table 8. Sample Initial processing

Sample	Initial processing	Aliquots	Initial transfer	Further processing	Ultimate use
Blood samples (serum)	Centrifuge 1500g for 10mins.	Supernatant: freeze at -80°C*	**Health Protection Research Unit for emerging and zoonotic infection (HPRU), Liverpool**	PHE or HPRU	Serology
		Supernatant: freeze at -80°C*		Imperial College London	Circulating mediators by multiplex cytokine/chemokine assays and proteomics
		Supernatant: freeze at -80°C*		PHE or HPRU	Mediators/proteomics other assays
Blood samples (EDTA)	Centrifuge 1500g for 10mins ideally at 4°C.	Supernatant: freeze at -80°C*		PHE or HPRU	Serology
		Supernatant: freeze at -80°C*		Imperial College London	Circulating mediators by multiplex cytokine/chemokine assays
		Supernatant: freeze at -80°C*		HPRU	Other studies (eg pharmacokinetics/ pharmacodynamics)
		Cell pellet: freeze at -80°C*		Roslin Institute (DNA extraction)	High-throughput genotyping and/or high coverage genome sequencing
Blood samples (Tempus RNA)	Freeze at -20°C	Where possible, freeze at -80°C* after 24hrs		Roslin Institute, (RNA extraction)	Microarray analysis and/or RNA seq analysis of host and pathogen RNA
CSF (if acquired)	Freeze at -80°C*	Aliquot if safe to do so into 3 aliquots Freeze at -80°C*		PHE or HPRU	Pathogen detection, quantification, viral genome sequencing and isolation
					Serology
					Circulating mediators by multiplex cytokine/chemokine assays and proteomics
Pathogen samples	**Do not process**	Freeze at -80 °C*		PHE or HPRU	Pathogen detection, quantification and viral genome sequencing and isolation.

*freeze at -80°C where possible, or at least at -20°C. If necessary (eg. weekends/public holidays) store in refrigerator until processing.

Sample processing should follow relevant PHE guidance.

5.4 Use of Stored Samples

Access to samples for additional analyses will be governed by a committee comprising the clinical lead investigators and scientific investigators for this study (the data and materials access committee), in collaboration with the individual recruiting sites. Linked anonymised data generated during the course of these studies may be shared between investigators. Each local site will hold their own data.

Where possible and within the constraints of international law and specific requirements of local ethical and institutional management approvals, anonymised clinical data will be shared centrally within one master database held in Oxford, which will be fully compliant with standard data management processes and local regulations. This database will be held on servers. Access to data for outside investigators will be reviewed by the independent data and materials access committee.

Samples will only be stored in containment facilities that have appropriate biological safety measures in place and have received necessary authorisation to store samples (according to national regulations for the pathogen being studied).

5.4.1 Research Plan for samples

This document is a standardized protocol for the rapid, coordinated clinical investigation of any emerging infections causing severe acute illness, or pathogen of public interest. The protocol is designed to have some level of flexibility in order to ensure the broadest acceptance and has been initiated in response to the recent cases of novel coronavirus (nCoV) in 2012-2013, Influenza H7N9 in 2013, viral haemorrhagic fever in 2014 and tick-borne encephalitis virus (TBEV) in 2019. However it is not limited to these pathogens. This protocol has been designed to maximize the likelihood that data and biological samples are prospectively and systematically collected and shared rapidly in a format that can be easily aggregated, tabulated and analysed across many different settings globally.

A high-level overview of research intentions by research institution is given here. However, no such plan can be predictive of future research questions and priorities. Investigators will meet to plan any such work before it commences. Samples may be used for commercial purposes, with appropriate consent.

Public Health England Laboratories at Colindale (Maria Zambon, Meera Chand, Samreen Ijaz) Porton Down (Tim Brooks) and the Health Protection Research Units in Liverpool (Calum Semple/Lance Turtle/Tom Solomon/Tom Fletcher), Glasgow (Massimo Palmarini, Antonia Ho), London (Peter Openshaw) and Edinburgh (Kenneth Baillie, Clark Russell) will be responsible for the primary processing and storage of samples obtained from patients (with appropriate biological safety measures in place) and pseudoanonymisation of samples prior to forwarding to other research institutions. Studies will include serology, proteomics, pathogen detection, quantification and viral genome sequencing both for diagnostic, public health and research purposes.

The Centre for Respiratory Infection (CRI) at Imperial College London (PI Peter Openshaw) will quantify soluble immune mediators using multiplex technology, in blood and

respiratory tract samples obtained. Cytokine, chemokine, proteomic and biomarker profiles will be correlated with clinical data and outputs from other laboratories within the study. Together, these data can be used to help understand pathogenesis, measure biological responses to novel treatments and help identify new therapeutic strategies. Serial serology tests will be used to characterise pre-existing and reactive adaptive immune responses.

Whole blood RNA tubes and cell pellets for DNA will be sent to the University of Edinburgh (PI JK Baillie) where DNA and RNA will be extracted. Host and pathogen transcriptomic analyses will be undertaken, including pathogen RNA and DNA sequencing and host gene expression profiling of whole blood RNA to identify and explore the interaction of host and viral factors during the course of infection. Where possible, genotype comparisons of affected individuals with population controls will be used to identify, characterise and confirm genetic associations with susceptibility to infection or severity of infection.

Liverpool (CI Calum Semple, Tom Solomon, Tom Fletcher & Lance Turtle) will conduct clinical characterisation studies based on clinical features and outcome in collaboration with ISARIC Clinical Coordination team (Gail Carson, Laura Merson). The University of Liverpool (CI Calum Semple, Tom Solomon & Lance Turtle), will if required provide additional capacity to contribute to the primary processing and storage of samples and then quantify soluble immune mediators using proteomics, multiplex technology, in CSF, blood and respiratory tract samples. Long read length sequencing using MinION will be used to characterize respiratory and pathogen samples from patients providing information on pathogen genotypes and the host transcriptome (Hiscox). This will be done either at the Royal Liverpool Hospital High Level Isolation Unit or within the CL3/CL2 laboratories at Liverpool, as appropriate.

Pharmacology studies (PI Saye Khoo) if included will be conducted at the University of Liverpool. We will measure drug exposure, and to relate this to patient characteristics (e.g. disease severity, liver or renal impairment, dialysis, children, pregnancy) and treatment response. Drug levels will be measured in plasma and other relevant samples.

Public Health England has a diverse portfolio of activity. It is anticipated that blood and oral fluid samples will be used to develop and validate diagnostic assays, and pathogen samples used to describe the molecular epidemiology of an outbreak.

Any use may include or lead to commercial development of diagnostic and therapeutic products and processes.

5.5 Future Use of Samples

All use of data and samples will be controlled by the Independent Data and Material Access Committee (IDAMAC; see below).

Samples collected will be used for the purpose of this study as stated in the protocol and stored for future use. The standard consent form will request consent from subjects for sample storage and/or export of specific samples to collaborating institutions for investigations, including commercial use.

Any proposed plans to use samples other than for those investigations detailed in this protocol will be submitted to the relevant ethics committees prior to any testing. Collaborating centres must have appropriate biological safety measures and regulatory approvals in place in order to receive samples.

Future use may include commercial development of diagnostic and therapeutic products and processes.

Any database detailing clinical data will only identify participants by a participant number. Participant names or any other identifying details will NOT be included. Data may be used alone or in combination with data from related studies in secondary analyses. The database containing personal identifiers and patient number (i.e. the key) will be held securely and encrypted by the study administrators on a University of Oxford server in a digitally distant location unlinked to that containing the clinical data and research data.

6 Medical Management and Safety Reporting

6.1 Medical Management

Medical management will be according to standard of care at the treating site and not a part of this research protocol. Research interventions include only collection of clinical information and specimens and therefore adverse event reporting is not applicable as there is no intervention.

7 Data Management

7.1 Data Collection

Clinical and laboratory data will be collected throughout the acute illness period according to local resources. Priority at all times will be given to the collection of clinical information. Research data will be integrated as much as possible with information available from hospital and regulatory files. Clinical data will be collected locally with the relevant CRF for SARI, VHF, CNS or other emerging infections of public health interest will be completed by a study staff as appropriate. The data will be anonymised at site and a study number issued.

7.2 Data Management

When available, data collected by staff at each site will be submitted electronically to a protected online database. Anonymised data may be entered by study staff in order to minimize the workload on site clinical staff. Quality checks will be built into the data management system and there will be quality control checks of critical data points entered into the CRFs to ensure standardization and validity of the data collected. The European Union General Data Protection Regulation (GDPR) and UK Data Protection Act regulations will be adhered to. Patients' identities will be protected and their information held

securely. The records kept will not include any information that allows patients to be identified.

For the Clinical Characterisation Protocol and the internal pilot study, access to the data entry system will be protected by username and password. Username and password will be assigned during the registration process for individual Site Investigators. All electronic data transfer between study site and database will be username and password protected. Each centre will maintain a trial file including a protocol, ethics approval documentation, and paper CRFs. A participant list will be used in each study site to match identifier codes in the database to individual patients in order to record clinical outcomes and supply any missing data points.

The Participant List (enrolment log) is maintained locally and is not to be transferred to any other location, except the ISARIC coordinating centre to allow linkage with laboratory research findings. The sites will compile an enrolment log including the patient's name, date of birth, hospital identification number and unique study number. Subsequent data will be identified by the unique patient study number only (consist of a 3-digit site code and a 4-digit patient number; see section on Case Report Form and Patient Numbers). The enrolment log and study data will be kept separately.

7.3 Data Access and Data Sharing

All use of data and samples will be controlled by the Independent data and materials access committee (IDAMAC).

7.3.1 Independent data and materials access committee (IDAMAC)

The IDAMAC will comprise seven senior, independent people with broad representation including research funders, academia, medicine, industry and public health. These individuals will not be involved in the acquisition or analysis of samples or data.

All samples, generated data and materials will be governed by the IDAMAC. The IDAMAC will authorise use of samples and dissemination of results. Disputes will be resolved by majority vote of the IDAMAC. The committee will meet virtually using teleconference every 3 months for the duration of the project, and on an ad hoc basis thereafter. New appointments to the IDAMAC to replace retiring members will be proposed by the chair and approved by majority vote of the committee.

7.3.2 Principles of data and materials access

The IDAMAC will facilitate and prioritise urgent investigations (from any sector, including public health, academic and commercial) with a high probability of impact in a given outbreak.

This study will adhere to the research policies of ISARIC (International Severe Acute Respiratory and Emerging Infection Consortium, www.isaric.org). A fundamental principle of this work is that clinical investigators contributing to research efforts, often in extremely

difficult circumstances, must be given full recognition for their efforts and the opportunity to access data and samples.

Data and results from central laboratory analysis for individual patients will be available to the clinicians looking after those patients as soon as possible. Often, this may not be in time to affect treatment decisions. Research data will be shared with public health authorities as needed.

7.4 Data Quality

Several procedures to ensure data quality and protocol standardisation will help to minimise bias. These include:

- An online start-up tutorial for all investigators prior to study commencement will be held to ensure consistency in procedures;
- A detailed data dictionary will define the data to be collected on the case report form;
- Quality checks will be built into the data management system and there will be quality checks of critical data points entered into the CRFs to ensure standardization and validity of the data collected;

Data queries may be generated, depending on resource availability. Any information that is not available for the investigator will not be considered as missing. No assumptions will be made for missing data.

7.4.1 Monitoring

Data monitoring will be conducted on a randomly selected subset (up to 5%) of cases, through discussion with the local site investigator to discuss data collection techniques. Direct site visits will not be feasible, given the scope of the study.

8 Ethical Considerations

This study is to be conducted during a disease outbreak or presentation of cases of disease of public health interest. This is a challenging research situation because this falls in the area between clinical care, public health and clinical research (WHO Ethical Review in Disease Outbreak Expert Meeting 2009). Normally research activities are defined by anything conducted outside standard clinical care. In these situations, there may be no definitive standard guidelines or treatment protocols and therefore there is often little difference between what can benefit the patients and what is very important for building knowledge on the pathogenesis of the disease to guide future treatment and management.

Medical management of participants in this study must never be compromised by study procedures. At all times, priority will be given to samples required for medical management. Research sampling should never compromise the quantity or quality of

samples taken for medical management, nor create a significant diversion for clinical teams from the day-to-day care of the patients.

8.1 Regulations, Guidelines and Ethical Review

This study will be conducted in compliance with the principles set out in the Declaration of Helsinki (Somerset West, 1996). Where applicable, the principles of Good Clinical Practice (ICH 1996) and other applicable regulations and guidelines will be used to guide procedures and considerations.

This protocol will be reviewed and approved by the ethical and regulatory review boards required by the recruiting site and the study sponsor. No patients will be enrolled until all approvals have been obtained for the applicable site.

8.2 Informed Consent

Consent forms will be provided in plain English. Illiterate participants will have the consent form read in the presence of a witness, who will sign to verify the accurate reading of the form and agreement of the participant. For participants who cannot understand the language of the available forms, verified translations will be made when possible. If it is not possible to prepare a translation in a required language, verbal translation of the document and the consent discussion (if required) will be used. In this case, the translator may act as the witness for consent and sign the consent form so that patients who cannot read the language of the forms are not excluded from this research.

In the case of adult participants who are unable to give informed consent due to mental or physical status, the wishes of the participant may be declared by an appropriate consultee according to the site policy on obtaining consent for medical procedures. If, during the course of the study, the participant's status changes such that they are able to consider consent independently, informed consent must be discussed and obtained.

An outbreak involving a pathogen of public health interest or pandemic is an emergency situation. For patients who are incapable of giving consent in emergency situations, the process of consent will comply in England and Wales with the Mental Capacity Act 2005 (MCA 2005); in Scotland, with Section 51 of the Adults with Incapacity (Scotland) Act 2000; and in Northern Ireland, with the 2016 Mental Capacity (Northern Ireland) Act. These are exceptions clearly acknowledged in the Declaration of Helsinki (2008), the following process will be observed:

- All efforts will be made to have consent from appropriate consultee /guardian/carer when available, and from the patient at the earliest opportunity.
- If a patient is incapable of giving consent and there is no relative/representative present, two doctors (one independent of the study team with knowledge of the patient condition) will consider the patient’s eligibility criteria and any known views of the patient about his/her participation. Together they will decide whether or not is appropriate to enrol the patient in the study.

Parents or guardians of children under the age of 16 years old will give consent for their child. Study staff obtaining consent will consider the ability of the child to understand the principles of the study and will discuss the study with the child in age appropriate language. Where appropriate, children will be invited to give assent, which will be recorded on the informed consent form. The right to withdraw at any time without negative impact will be reinforced with the child and their parent/guardian. Should the UK rules on consent by young people for research purposes alter during the period of this study to allow consent by competent minors, then these new rules will be applied to this study without further amendment.

In the United Kingdom (England, Wales, Northern Ireland and Scotland); where competent young people age 16 years and older (but younger than 18 years old) have given consent, their parent(s) or person(s) with parental authority should be informed of the young person's decision and they should be given a copy of the study information. A contemporary record should be made in the clinical notes that this information has been shared with the parent(s) or person(s) with parental authority. In other countries local ethical regulations will apply

A copy of the informed consent form will be given to the person who gives consent.

8.3 Alternatives to Participation and Withdrawal

Prospective participants are freely able to decline participation in this study or to withdraw from participation at any point without suffering any implied or explicit disadvantage. All patients will be treated according to standard practice regardless of if they participate.

8.4 Risks to Participants

Inconvenience. Participation in this research study poses a minimal risk of inconvenience through household visits and attendance of follow-up visits. Appropriate compensation for travel costs to attend follow-up visits and for time of attending visits will be given according to the standard policies of the sponsor.

Phlebotomy. Participants may have blood drawn more often than is required for standard care. Phlebotomy can be associated with pain at the draw site and rarely with infection. Daily blood draw volumes have been restricted according to weight so that combined clinical and research sampling is within recommended limits. Discomfort will be minimized by having expert staff obtain blood samples, and by combining research sampling with routine clinical sampling, where possible, which normally occurs daily in acutely unwell patients in hospital.

Discomfort of throat swabs. Collecting throat swabs may be cause transient discomfort. Discomfort and risk will be minimized by using experienced clinical staff at each site, and samples will be taken at the same time as clinical samples in order to minimize these risks.

Discomfort of SAM strips. Collecting nasal fluid using SAM strips may be cause a transient tickling sensation during application and removal which can cause eye watering through a local reflex.

Oral (Crevicular) Fluid Collection. Oral crevicular fluid collection involves the participant or carer gently brushing a small sponge on a flexible plastic rod at the margin of the gums and teeth in exactly the same manner as is done for routine mouth care or teeth brushing. Apart from inconvenience and sensation, there is no expectation of and discomfort.

Lumbar puncture. Collection of cerebrospinal fluid with lumbar puncture will only be performed if clinically indicated, as decided by the responsible physician. Clinical investigations are the priority, with any remaining sample collected for use in research. Guidance on the safe recommended daily total volume of CSF to take in different age groups is provided (Table 4). Lumbar puncture can be associated with discomfort at the site of needle insertion, headache, and rarely bleeding or infection.

Incidental findings in genetic testing. This study includes genetic testing to identify host genetic variants associated with disease progression or severity. There is a very small chance that these tests may result in the incidental discovery of information that is relevant to the participant's health. Since the samples will be analysed anonymously in batches, and generally in non-clinical laboratories with investigational techniques, we will not attempt to identify and inform participants of any results from genetic tests. If we were to do so, there would be a considerable risk of accidental harm in the form of unnecessary anxiety and distress.

Specific risks for VHF patients. Participants with VHF may be at increased risk of bleeding from venepuncture sites. The decision to perform venepuncture for research purposes will only be performed following discussion with the attending clinician and only if venepuncture is deemed not to pose unacceptable risk to the patient and/or staff. When at risk venepuncture will be minimised by limiting research venepuncture to coincide with clinical venepuncture.

8.5 Benefits to Participants

There will be no direct benefit to research participants. The study may include biological sampling in addition to sampling required for medical management. The results of the tests done on these samples may not contribute to improving the participant's health. The results of this study will not be available in time to contribute to the participant's care. Where possible, test results with potential relevance to patient care will be informed to the participant and/or treating doctor. The feasibility of this will depend on local resources. Some assays cannot immediately benefit the patient because data will need to be pooled with others, or because the assays take time.

8.6 Participation in Other Research Studies / Co-enrolment

Particularly in the case of emerging infections, it is likely that other research projects, including clinical trials, will also recruit participants in this study. In fact, it is important that they do so, and great effort has been expended to ensure that this observational study

is compatible with, and complementary to, other possible research projects. However, in the UK this study has been given NIHR Clinical Research Network expedited urgent public health study status. In the event of an outbreak the study will be given priority by the Clinical Research Network. All Comprehensive Local Research Networks (CLRN) have Urgent Public Health plans, which will be activated in the event of an outbreak. In practical terms this means that where research resources are limited, this study may take precedence over others.

8.7 Confidentiality

This study will be conducted by clinical staff and those involved in the study will ensure that each study participant's privacy and confidentiality is maintained. Participants will not be identified in any published reports of this study. All records will be kept confidential to the extent provided by international and local law. All laboratory specimens, evaluation forms, reports, study protocol, documentation, data and all other information generated will be held in strict confidence. No information concerning the study, or the data will be released to any unauthorized third party.

Minimal personal data will be entered into the database for analysis. The patient's identifying personal information will be logged separately and stored securely. The patient might be asked to take part in future research, and therefore their identifiers need to be retained for contact at a future date, subsequent to the appropriate ethical approvals. The stored research data is also likely to be of significant value in the future for other studies and therefore permission is sought for this storing of the research data that does contain minimal patient identifiers such as age, sex and ethnicity.

Paper and electronic medical records may be accessed during the study to confirm, verify or complete clinical information provided in the case report form.

Site files will at all times be accessible only to clinical and research staff. Consent will be sought for investigators to access patient data. Local research staff will access personal information, but all data will be pseudoanonymised before transfer by eCRF.

At the Public Health England Laboratories and Health Protection Research Units in Liverpool all research samples will be labelled with a unique, non-identifiable subject number. The patient's name and subject number will be recorded on the consent form. This will preserve the link between anonymous and identifiable data. Data from routine clinical care will be anonymised and stored separately to laboratory samples. Samples obtained will be anonymised where possible however source samples containing high consequence pathogens of public health interest cannot be anonymised for important safety reasons so will be held under very strict security measures (a home office approved high security facility) by staff who do not have access to clinical data. The only link to identifiable clinical data will be the consent form. Further research questions, subject to appropriate ethical approval, may be answered in retrospect in the future. Since the samples and data generated by this work may be irreplaceable after an outbreak of infectious disease has passed, it is essential that future work is not impeded by unnecessary data loss.

Anonymised research data will be stored on managed computer systems in Imperial College London, University of Liverpool, PHE, the University of Edinburgh, the Roslin Institute and other investigator sites relevant to the laboratory tests they have done. Only the study administrators will hold the data set key and this will be separated from the personal identifiers. Data will be encrypted before transfer on portable devices. Multiple backups will be maintained on institutional servers. Critical data will be stored in encrypted form in a stable storage format with the passwords recorded on paper in securely held site files in these locations.

It is important that data generated now is not destroyed unnecessarily, since they will be of considerable potential value to future generations faced with similar outbreaks of infectious disease. Electronic data and electronic copies of paper documents will be stored indefinitely.

8.8 Custody of Data and Samples

Custody of site data will remain with the responsible physician at the site. Samples will be shipped (depending upon pathogen of interest) to a central laboratory (the Public Health England Laboratories at Colindale or Porton Down or the Health Protection Research Units at Liverpool and London), for processing and pseudoanonymisation and later forwarded to research institutions for analysis as approved by the appropriate ethics/institutional review committee. Any residual samples will remain in the custody of the site until use can be decided upon according to ISARIC policies/procedures. Centralized data will be in the custody of the University of Oxford (ISARIC Coordination Centre). A data sharing policy will be put in place between Universities of Liverpool, Oxford and the research laboratories.

8.9 Additional Ethical Considerations

No consent for Tier Zero (Collection of anonymised limited routine clinical data).

This does not require consent. This is because the patient is not identifiable and the data is collected by a health care professional who has access to this information by virtue of their clinical role.

Recruitment of critically ill patients who are not able to consent. This is a ubiquitous problem in acute and critical care research and there is a clear legal framework under which these patients may be recruited to research studies. In all cases, efforts will be made to obtain informed consent from patients early in the course of illness, before critical illness interferes with their capacity to make decisions and to confirm consent at the earliest point in recovery. This principle applies equally to adults and children.

Perceived coercion because of individual responsibilities to society, and the implications of this research for public health. We are sensitive to the fact that some patients or their representatives may feel under an unusually strong moral obligation to participate given the nature of this research and the wide, and often inaccurate, publicity surrounding emerging infections. In view of this, we have tried to make both the potential benefits and limitations of this simple observational study clear in the information sheet. In

the informed consent form, we also stress that participation is entirely voluntary and there is no penalty of any kind for declining to join the study.

Balance between public health and research. Patients with emerging infections are commonly the subject of public health investigations. The work proposed here is research and will be clearly presented as such. There is no primary gain to the patient from participating. In designing and describing this research we are clear that, in accordance with the guiding principles of Good Clinical Practice, the needs and autonomy of the individual are paramount and the potential benefits to wider society do not take precedence.

Risks to clinical and research staff treating the participants. Staff who enrol, examine and take samples from study patients are at risk of infection. Care of study participants will require increased sampling and contact frequency added to normally heavy clinical workloads. All staff must be trained in recognised infection control measures and have ready access to appropriate personal protective equipment. In collaboration with the public health authorities, there will be on-going communication with hospital staff to ensure the appropriate training is given, to support the work and to ensure that there is no excess burden on the health system. Where appropriate, dedicated research staff will be available to support the study activities.

8.10 Insurance

The University of Oxford has arrangements in place to provide for non-negligent harm arising from participation in the study for which the University is the Research Sponsor. However, if the study involves minimal deviation from normal clinical care, non-negligent harm cover may not apply.

8.11 Scientific and Peer Review

The proposed research began as the product of a year-long discussion (2011-2012) within a group of international experts who were brought together following the 2009 influenza pandemic to plan the global research response to future severe and emerging infections: the International Severe Acute Respiratory and Emerging Infection Consortium (ISARIC). ISARIC working group 3 (genomics, pathogenesis and pharmacology; Chair, JK Baillie) comprised senior clinical scientists from 5 continents, and aims to promote and harmonise observational research during outbreaks of severe infectious disease.

The current version has been extensively reviewed by scientists across the world since 2012.

Material Transfer Agreement for the Supply of Human Tissue Materials FOR USE where the material is human organs, tissue or cells (other than human gametes or embryos) but NOT where the intended use is transplantation or human application

This Agreement is made by and between:

a) "the Provider Institutions" being the NHS Trusts giving local R&D approval to this protocol

and

b) University of Liverpool

University of Oxford

University of Edinburgh

University Glasgow

Imperial College London

Wellcome Trust Sanger Institute

Global Health Network, Oxford

Reference Microbiology Services, Public Health England

University of Southampton

NHS Blood & Transplant Blood Borne Virus Unit

the above being "the respective Recipient Institutions"

This Agreement records the terms under which the Provider Institution will make available to the Recipient Institutions the Material identified in the protocol (the "Material"). The term "Material" means material, other than human gametes or embryos, which consists of, or includes human cells and which is considered "Relevant Material" for the purposes of the Human Tissue Act 2004[1] together with related data. The Recipient Institution will hold the Material on the terms of this Agreement and solely for the purpose of "the Study" and as described the protocol, within the research groups ("the Recipient Scientists"). The Recipient Institutions hereby agrees to comply and procure that the Recipient Scientists and all personnel who work with the Material comply with the following terms and conditions:

1. The Recipient Institutions will not use the Material for administration to human subjects or human application as that term is defined in the Human Tissue (Quality and Safety for Human Application) Regulations 2007 (or equivalent as each may be replaced or amended from time to time), or for clinical or diagnostic purposes.[2]

---

[1] The Human Tissue Act 2004 applies to the "authorised activities" principally the removal, storage and use of "Relevant Materials" (as defined under the Act, including human cells, tissue and organs, but not cell lines) which come from a living or deceased person for "Scheduled Purposes" (these are set out in Schedule 1 of the Act, including, but not limited to, "research in connection with disorders, or the function of the human body", "education or training relating to human health", and "transplantation").

[2] The Human Tissue (Quality and Safety for Human Application) Regulations 2007 apply to the procurement, testing, processing, storage, distribution, and import or export of tissues and cells (including cell lines). "Cells" mean human cells (whether individually or in an unbound collection) including cell lines, but not including gametes, embryos outside the

2. The Recipient Institution may use the Material for the purposes of the Study and as described in the protocol, from the date of receipt of the Material. The Recipient Institution will comply fully with all applicable environmental, health and safety laws, the Human Tissue Act 2004 and other Applicable Laws[3] with respect to its use (including, but not limited to, disposal or return).
3. The Recipient Institution shall use a courier with suitable skill and experience to safely transport the Material in accordance with all Applicable Laws. The Recipient Institution will bear the cost of carriage and any necessary insurance. The Provider Institution makes no charge for the Material / the Material is provided subject to the reimbursement by the Recipient Institution to the Provider Institution for its costs of extracting from storage and preparing the Material as set out in the protocol. Risk in and responsibility for the Material shall pass to the Recipient Institution once it is loaded onto transport as organised by the Recipient Institution. If so requested by the Provider Institution the Recipient Institution shall provide it with written confirmation of the safe receipt of the Materials promptly after their delivery to the Recipient Institution's laboratory.
4. The Recipient Institution understands that the Material may have hazardous properties, contain infectious agents or pose other health and safety risks. Subject to clause 9, the Provider Institution makes no representations and gives no warranties either express or implied in relation to it: for example (without limitation), no warranties are given about quality or fitness for a particular purpose, or freedom from infection. The Provider Institution will not be liable for any use made of the Material by the Recipient Institution. The Recipient Institution will use the Material in accordance with good laboratory practice standards, all due skill and care and with dignity, sensitivity and respect. The Recipient Institution will comply with all Applicable Laws, approvals, rules, codes of practice and regulations governing the transportation, storage, use and disposal of the Material. The Recipient Institution warrants that it will only use, or permit the use of the Material in work that has ethical approval, as stated in the protocol.
5. Except to the extent prohibited by Law and subject to clause 9, the Recipient Institution assumes all liability for damages which may arise from its receipt, use, storage or disposal of the Material. The Provider Institution will not be liable to the Recipient Institution for any loss, claim or demand made by the Recipient Institution, or made against the Recipient Institution by any other party, due to or arising from its use, storage or disposal

---

body, blood or blood components. "Tissue" for the Regulations, means all constituent parts of the human body formed by cells, but not including gametes and embryos outside the body (which are regulated by the Human Fertilisation and Embryology Authority pursuant to the Human Fertilisation and Embryology Act 1990), or organs.

[3] Applicable Laws means all laws, rules, regulations, codes of practice, research governance or ethical guidelines, or other requirements of any Regulatory Authority, that may apply to the use of the Material by the Recipient Institution from time to time, including (but not limited) the Human Tissue Act 2004 or the Human Tissue (Scotland)_Act 2006, the Human Tissue (Quality and Safety for Human Application) Regulations 2007, the Human Fertilisation and Embryology Act 1990 (as amended), the EU Tissues and Cells Directive (2004/23/EC) and Commission Directives 2006/17/EC and 2006/86/EC. The Human Tissue Authority Directions and Codes of Practice, and the Medicines for Human Use (Clinical Trials) Regulations 2004, as updated and amended from time to time and, where relevant, the national implementations of the same.

of the Material by the Recipient Institution, except to the extent the law otherwise requires.

6. The liability of either party for any breach of this Agreement, or arising in any other way out of the subject matter of this Agreement, will not extend to loss of business or profit, or to any indirect or consequential damages or losses.
7. The Recipient Institution agrees to obtain the written consent of the Provider Institution if there is any material change to the proposed use of the Material in the Study as described in the protocol.
8. The Recipient Scientist will acknowledge the source of the Material in any publication reporting on its use. If the Recipient Scientist wishes to include in a publication any information which has been provided by the Provider Institution with the Material and which was clearly marked as “confidential” and “proprietary” at the point of disclosure (“Confidential Information”), the Recipient Scientist must obtain written permission from the Provider Institution, providing a copy of the text to allow a reasonable period for review before publication takes place, such permission not to be unreasonably withheld or delayed. If so requested by the Provider Institution, the Recipient Institution shall provide the Provider Institution with a confidential copy of the findings of the Study.
9. The Provider Institution warrants that where required by Applicable Laws the Material has been obtained from humans with the appropriate consent as required by the Human Tissue Act 2004 and with ethical approval and the Provider Institution shall be liable for any claims arising due to the breach of this warranty. The Provider Institution hereby grants to the Recipient Institution a non-exclusive research licence to use the Material for the Study only. The Provider Institution further warrants that it has not provided any information (and does not intend to provide any information) which has led or may lead to the Recipient Institution being able to identify the person from whom the relevant material came.
10. The Recipient Institution undertakes to store the Material in accordance with all Applicable Laws and not to attempt to identify or contact the donor of the Material or to compromise or otherwise infringe the confidentiality of information on the donors and their right to privacy.
11. Nothing included in this Agreement shall prevent the Provider Institution from being able to distribute the Material to other entities as described in the protocol. If, as per the details included in the protocol, the Material is to be transferred to another institution for the purposes of the Study, the responsibility for compliance with the terms of this Agreement rests with the Recipient Institution.
12. The Provider Institution has the right to terminate this agreement forthwith at any time by means of written notice to Recipient Institution if the ethical approval is withdrawn or if the Recipient Institution is in breach of this Agreement. In the case of any termination, the Recipient Institution shall immediately discontinue all use of the Material and, at the Provider Institution's discretion, promptly return or destroy (at the Recipient Institution's own cost) all unused Material and provide written confirmation that this has been completed. If requested, the Recipient Institution must certify that it has complied in full with any such requirement of the Provider Institution. Should an individual donor or their next of kin rescind their consent, the Provider Institution will require and the Recipient Institution agrees to discontinue using the appropriately identified sample and return or destroy it in accordance with the Provider Institution’s instructions.
13. This Agreement shall be governed by English Law, and the English Courts shall have exclusive jurisdiction to deal with any dispute which may arise out of or in connection with this Letter Agreement.

END

8.12 Revision History

8.12.1 Changes since v7.3

Date and Version No: v8.2 17FEB2020

In section 1.1 Purpose of the Study, expansion of study to include an annual one week activation – internal pilot study to maintain and test readiness of the study activation that includes data collection only on severe acute respiratory infection (SARI). The anonymised data collected from the annual activation will be also shared with an international project aimed at characterising SARI patients to better inform management strategies and ultimately to improve clinical management of emerging infectious causes of SARI (SPRINT-SARI).

The Background information of A/H5N1, MERS CoV and A/H7N9 in section 1.2 has been revised and updated.

Section 1.6.1 has been added with specific objectives of annual activation (internal pilot)

Amends in section 1.7 Structure of this document to reflect the inclusion of the pilot study for data collection only corresponding to TIER ZERO within the main tier structure of the Clinical Characterisation Protocol.

In section 1.8 updated detail Entry Criteria for SARI patients for the pilot study included in Appendix A. To be consistent with the background of this protocol, it has been added a clarification on the entry criteria for patients with suspected or confirmed infection with a pathogen relevant to public health interest.

Clarification in section 3.1 the sample size is not prospectively determined. The study has no set end date.

Inclusion in Section 3.2 Approach to Potential Participants of explanatory paragraph on the process for consent within emergency situation to enter a patient into the study when the patient is unconscious or incapacitated to give consent and there is no relative/representative present, a nominated consultee will be sorted. The process of consent will comply in England and Wales with the Mental Capacity Act 2005 (MCA 2005); in Scotland, with Section 51 of the Adults with Incapacity (Scotland) Act 2000; and in Northern Ireland, research provisions of the 2016 Mental Capacity Act (Northern Ireland).

This is acknowledge within the Helsinki Declaration (2008). Emphasising that every effort will be made to get consent from representatives where they become available and/or from the patient when he/she is able to do so.

It is explained that for studies that submit collect or collate only pre-identified anonymised data that is normally collected as part of routine care information and where involvement in the research carries no more than low risk, such as the pilot study, consent may not be required. In the annual activation pilot study in order to test the processes within the

overarching Clinical Characterisation Protocol, consent will be obtained from patient/consultee/parent/guardian/career.

Clarification in Section 3.6 Enrolment Procedures for Patients that meet the inclusion criteria will be have their clinical information collected and entered in the study when consent has been obtained, be it deferred, proxy or assent. Clarifying that to annually activate the study for one week - the selection criteria and data collection will follow the procedures indicated in the in Appendix A

In section 4.1 Specimen Sampling, Storage Procedures and Transport, there is a clarification on the management of other merging pathogens, indicating that these pathogens may be classified as requiring BSL2, BSL3 or BSL4 for safety management and guidelines should be consulted as per hospital protocol. Samples that are not BSL3/4 may be stored locally at participating sites.

In section 4.2. Removal of need for consent for Tier Zero activity (collection of de-identified data only').

Inclusion in section 6.2 Data Management of explanatory paragraph to clarify that the entry of data for the Clinical Characterisation Protocol and pilot study user name and password are assigned during registration. Transfer of data is anonymous and password protected. Each site will maintain a trial file including a protocol, ethics approval documentation, and paper CRFs. A participant list will be used in each study site to match identifier codes in the database to individual patients in order to record clinical outcomes and supply any missing data points. The Participant List is maintained locally and is not to be transferred to any other location. The enrolment log and study data will be kept separately.

Explanation in section 7.2 Informed Consent, in the case of an outbreak of public health interest, for patients who are incapable of giving consent in emergency situations, the process of consent will comply in England and Wales with the Mental Capacity Act 2005 (MCA 2005); in Scotland, with Section 51 of the Adults with Incapacity (Scotland) Act 2000; and in Northern Ireland, research provisions of the 2016 Mental Capacity Act (Northern Ireland).

These are an exceptions acknowledged in the Declaration of Helsinki (2008), the following process will be observed:

• If a patient is incapable of giving consent and there is not relative/representative present, two doctors (one independent of the study team with knowledge of the patient condition) will consider the patient's eligibility criteria and any known views of the patient about his/her participation. Together they will decide whether or not is appropriate to enrol the patient in the study.

•All efforts will be made to have consent from appropriate consultee /guardian/carer when they became available, and from the patient at the earliest opportunity.

Regarding consent by competent minors, should the UK rules on consent by young people for research purposes alter during the period of this study to allow consent by competent minors, then these new rules will be applied to this study without further amendment.

To reflect the above changes in protocol single patient information and consent / assent forms have been created instead of being virus specific.

At the end of protocol inclusion of APPENDIX A CCP Annual Activation Guidance (a separate document).

Reformatting of protocol and consent forms for easier maintenance and sharing.

At the request of the Chief Medical Officer, we have transferred governance of the samples to the IDAMAC. The protocol is updated to reflect this.

Additional optional sub studies are included describing the following samples and procedures:

- additional sample for isolation of PBMCs

- environmental transmission assessment.

- plasmapheresis for research, standards and product development

8.12.2 Changes from (Previous version v7.0 25AUG2014)

Date and Version No: 21 March 2016 v7.3

UK CRN ID14152 (now included in first page of Protocol)

The most significant change in the protocol "Clinical Characterisation Protocol for Severe Emerging Infections", is an amend to activate the sleeping protocol for one week in each winter season for data collection of SARI in nominated centres. This short term activation of the protocol will maintain and test the protocol in order that it remain prepared to be activated in the event of an outbreak / pandemic of an pathogen of public health interest. This annual activation will serve as an internal pilot to test and maintain the main study processes.

In addition there is a clarification on the inclusion criteria for patients presenting with an infection by a pathogen of public health interest.

Inclusion of new co-investigators: Prof Richard Tedder, PHE and NHS Blood & Transplant Blood Borne Virus Unit, and Prof Peter Horby, Professor of Emerging Infectious Diseases and Global Health, University of Oxford.

The institutional affiliation of two co-investigators has been updated to reflect their present role: Dr Jake Dunning is now at Public Health England, and Laura Merson, is now at University of Oxford in England.

8.13 APPENDIX A

8.14 Test Activation Guidance – Internal Pilot Study for maintenance of the UK Clinical Characterisation Protocol

To be used in combination with this protocol
