# Supplementary material for: Features of 20 133 UK patients in hospital with covid-19 using the ISARIC WHO Clinical Characterisation Protocol: prospective observational cohort study
Source: BMJ. 2020 May 22;369:m1985. doi: 10.1136/bmj.m1985 (PMC7243036; doi:10.1136/bmj.m1985)
Supplement: Supplementary file 3 — Web appendix: Supplementary material 3—case report form [file doca057402.ww3.pdf]

ISARIC/WHO Clinical Characterisation Protocol for Severe Emerging Infections UK (CCP-UK) Case Report Form

V9.2 26FEB2020

DESIGN OF THE CCP-UK CASE REPORT FORM (CRF)

This CRF is divided into a "**CORE**" form (3 pages) with presentation data, a "**DAILY**" form (2 pages) for daily clinical and laboratory and data, and an "**OUTCOME**" form (3 pages). There is also a **TRAVEL AND ANIMAL EXPOSURE** form (1 page) which should be used when appropriate.

HOW TO USE THIS CRF

The CRF is designed to compliment the **Tier** of activity that a site has capacity and capability to work to. This is likely to vary over the course of an outbreak. The decision on which tier to use is up to the Local Principal Investigator. All high-quality data is valuable for analysis.

Ideally, data and samples will be collected with consent using Tier 2 of the protocol schedule, as outlined below. This will be of greatest public health research value in the early stages of an outbreak.

Data can be collected for Tier Zero activity without consent.

Consent must be obtained for biological sampling and data collection for Tier 1 and Tier 2 actiivty.

Tier Zero - For sites where caseload or facilities limit research capacity to deliver Tier 1 or Tier 2 activity.

OR

- For collection of data without consent.

Please complete the **CORE CRF** and **DAILY CRF** for the first day of hospital admission (day 1), the **DAILY CRF** for the third (d3), sixth (d6) and ninth (d9) days, then the **OUTCOME CRF** at discharge or death.

Tier 1 - For sites where facilities limit research capacity to deliver Tier 2 activity. With consent for single timepoint biological sampling. Please complete the **CORE CRF** and **DAILY CRF** for the first day of hospital admission (day 1), the **DAILY CRF** for the third (d3), sixth (d6) and ninth (d9) days, the **DAILY CRF** again for the first day of any ICU admission, and then the **OUTCOME CRF** at discharge or death.

Tier 2 - For sites with available resources to deliver Tier 2 activity per the protocol schedule. With consent for multiple timepoint biological sampling. Please complete the **CORE CRF** and **DAILY CRF** on the first day of hospital admission. Please complete the **DAILY CRF** on each subsequent day up to discharge or death. Please complete the **OUTCOME CRF** at discharge or death.

ISARIC/WHO Clinical Characterisation Protocol for Severe Emerging Infections (UK)

SARI Case Report Form FRONT PAGE 2 of 2

GENERAL GUIDANCE

- The CRF is designed to collect data obtained through examination, interview and review of hospital notes. Data may be collected retrospectively if the patient is enrolled after the admission date.
- Participant Identification Numbers consist of a 5-digit CPMS site code and a 4 digit participant number. You can obtain a site code by contacting your local R&D office or CCP@liverpool.ac.uk . Participant numbers should be assigned sequentially for each site beginning with 0001. In the case of a single site recruiting participants on different wards, or where it is otherwise difficult to assign sequential numbers, it is acceptable to assign numbers in blocks or incorporating alpha characters. E.g. Ward X will assign numbers from 0001 or A001 onwards and Ward Y will assign numbers from 5001 or B001 onwards. Enter the Participant Identification Number at the top of every page.
- Case Report Form Data should be entered to the central database at https://ncov.medsci.ox.ac.uk
- REDCap registration access is obtained by contacting ncov@isaric.org please state "[CCP-UK REDCap ACCESS]" in the title
- Please contact us at ncov@isaric.org we can help with database problems
- In the case of a participant transferring between study sites, it is preferred to maintain the same Participant Identification Number across the sites. When this is not possible, space for recording the new number is provided.
- Complete every line of every section, except for where the instructions say to skip a section based on certain responses.
- Selections with square boxes (☐) are single selection answers (choose one answer only). Selections with circles (**O**) are multiple selection answers (choose as many answers as are applicable).
- Mark 'N/A' for any results of laboratory values that are not available, not applicable or unknown.
- Avoid recording data outside of the dedicated areas. Sections are available for recording additional information.
- We recommend writing clearly in black ink, using BLOCK-CAPITAL LETTERS.
- Place an (X) when you choose the corresponding answer. To make corrections, strike through (-------) the data you wish to delete and write the correct data above it. Please initial and date all corrections.
- Please keep all of the sheets for a single participant together e.g. with a staple or participant-unique folder.
- DO NOT SEND CRFs to anyone by email or post.
- These two FRONT PAGES do not need to be retained.

ISARIC/WHO Clinical Characterisation Protocol for Severe Emerging Infections
SARI Case Report Form
CORE CASE RECORD FORM

Date of enrolment [_D_][_D_]/[_M_][_M_]/[_2_][_0_][_Y_][_Y_] **Site Location**___________________________

CLINICAL INCLUSION CRITERIA

Proven or high likelihood of infection with pathogen of Public Health Interest ☐ YES ☐ NO

Experience of the following symptoms during this illness episode: (one or more required for inclusion)

A history of self-reported feverishness or measured fever of ≥ 38°C: ☐ YES ☐ NO
Cough: ☐ YES ☐ NO
Dyspnoea (shortness of breath) OR Tachypnoea*: ☐ YES ☐ NO
Clinical suspicion of ARI despite not meeting criteria above: ☐ YES ☐ NO

** respiratory rate ≥50 breaths/min for <1 year; ≥40 breaths/min for 1-4 years; ≥30 breaths/min for 5-12 years; ≥20 breaths/min for ≥13 years*

EPIDEMIOLOGICAL FACTORS

In the 14 days before onset of illness had any of the following:

A history of travel to an area with documented cases of infection of a respiratory pathogen of public health Interest in the context of an outbreak, suspected outbreak or incident of a respiratory pathogen of public health interest
☐ YES ☐ NO ☐ Not known

Close contact* with a confirmed or probable case of infection with the respiratory pathogen of public health interest, while that patient was symptomatic

☐ YES ☐ NO ☐ Not known

Presence in a healthcare facility where infections caused by the respiratory pathogen of public health interest have been managed
☐ YES ☐ NO ☐ Not known

Presence in a laboratory handling samples suspected or confirmed of having the respiratory pathogen of public health interest present
☐ YES ☐ NO ☐ Not known

An otherwise unexplained respiratory illness in the context of an outbreak, suspected outbreak or incident of a respiratory pathogen of public health interest
☐ YES ☐ NO ☐ Not known

Direct contact with animals in countries where the pathogen of public health interest is known to be circulating in animal populations or where human infections have occurred as a result of presumed zoonotic transmission
☐ YES ☐ NO ☐ Not known

* Close contact' is defined as:
- Health care associated exposure, including providing direct care for patients, e.g. health care worker, direct exposure to body fluids or specimens including aerosols, working with health care workers infected with the pathogen of public health interest, visiting patients or staying in the same close environment of relevant case.
- Working together in close proximity or sharing the same classroom environment with a relevant case
- Traveling together with in any kind of conveyance with a relevant case
- Living in the same household as a relevant case

DEMOGRAPHICS

Sex at Birth: ☐ Male ☐ Female ☐ Not specified ***Date of birth** [_D_][_D_]/[_M_][_M_]/[_Y_][_Y_][_Y_][_Y_]

*** WHERE DATA IS BEING COLLECTED WITHOUT CONSENT FOR TIER ZERO, ONLY RECORD AGE AND NOT DATE OF BIRTH**
If date of birth is unknown or for Tier Zero, record Age [___][___][___]years OR [___][___]months

Ethnic group *(check all that apply)*:

O Arab O Black O East Asian O South Asian O West Asian O Latin American O White O Aboriginal/First Nations

O Other: ________________________ ☐N/A

Employed as a Healthcare Worker? ☐YES ☐NO ☐N/A

Employed in a Microbiology laboratory? ☐YES ☐NO ☐N/A

Pregnant? ☐ YES ☐ NO ☐ Unknown ☐ N/A **If YES: Gestational weeks assessment:** [___][___] weeks

POST PARTUM (within six weeks of delivery)? ☐YES ☐NO or N/A *(skip this section - go to INFANT)*

Pregnancy Outcome: ☐Live birth ☐Still birth **Delivery date:** [_D_][_D_]/[_M_][_M_]/[_2_][_0_][_Y_][_Y_]

Baby tested for Mother's ARI infection? ☐YES ☐NO ☐N/A **If YES:** ☐Positive ☐Negative **Method:** ☐PCR ☐Other:_________

INFANT – Less than 1 year old? ☐YES ☐NO *(skip this section)* **Birth weight:** [___][___].[___]☐kg or ☐lbs ☐N/A

Gestational: ☐ Term birth (≥37wk GA) ☐Preterm birth (<37wk GA) **if <37wk** Estimated gestation _________weeks ☐N/A

Breastfed? ☐YES ☐NO ☐N/A **If YES:** ☐Currently breastfed

☐Breastfeeding discontinued at [___][___]weeks☐N/A

Appropriate development for age? ☐YES ☐NO ☐N/A

Vaccinations appropriate for age/country? ☐YES ☐NO ☐Unknown ☐N/A

ONSET AND ADMISSION

Symptom onset date of first/earliest symptom: [_D_][_D_]/[_M_][_M_]/[_2_][_0_][_Y_][_Y_]

Admission date at this facility: [_D_][_D_]/[_M_][_M_]/[_2_][_0_][_Y_][_Y_]

Time of admission (24-hour format):[_H_][_H_]/[_M_][_M_]

Transfer from other facility? ☐YES-facility is a study site ☐YES-facility is not a study site ☐NO ☐N/A

If YES: **Name of transfer facility**:____ ☐N/A

If YES: **Admission date at transfer facility** *(DD/MM/YYYY)*: [_D_][_D_]/[_M_][_M_]/[_2_][_0_][_Y_][_Y_] ☐N/A

If YES-Study Site: **Participant ID # at transfer facility:** ☐Same as above

☐Different: [___][___][___]–[___][___][___][___] ☐N/A

Travel in the 14 days prior to first symptom onset? ☐YES ☐NO ☐N/A *If YES, complete the*

If YES, *state location(s) & date(s):* Country:______________________ City/Geographic area:______________________

Return Date: [_D_][_D_]/[_M_][_M_]/[_2_][_0_][_Y_][_Y_] ☐ N/A *(if more use TRAVEL EXPOSURE CRF)*

Contact with animals, raw meat or insect bites in the 14 days prior to symptom onset?

☐YES ☐NO ☐Unknown ☐ N/A *If YES, complete the ANIMAL EXPOSURE CRF*

SIGNS AND SYMPTOMS AT HOSPITAL ADMISSION *(first available data at presentation/admission – within 24 hours)*

Temperature: [_][_][_].[_]☐☐°C *or* ☐☐°F **HR:** [_][_][_]beats per minute **RR:** [_][_]breaths per minute

Systolic BP: [_] [_] [_]mmHg **Diastolic BP:** [_][_][_]mmHg **Severe dehydration:** ☐YES ☐NO ☐Unknown

Sternal capillary refill time >2seconds ☐YES ☐NO ☐Unknown

Oxygen saturation: [_][_][_]% **On:** ☐Room air ☐Oxygen therapy ☐N/A

Admission signs and symptoms *(observed/reported at admission and associated with this episode of acute illness)*

History of fever	☐YES ☐NO ☐Unknown	Lower chest wall indrawing	☐YES ☐NO ☐ Unknown
Cough	☐YES ☐NO ☐ Unknown	Headache	☐YES ☐NO ☐ Unknown
with sputum production	☐YES ☐NO ☐ Unknown	Altered consciousness/confusion	☐YES ☐NO ☐ Unknown
bloody sputum/haemoptysis	☐YES ☐NO ☐ Unknown	Seizures	☐YES ☐NO ☐ Unknown
Sore throat	☐YES ☐NO ☐ Unknown	Abdominal pain	☐YES ☐NO ☐ Unknown
Runny nose (Rhinorrhoea)	☐YES ☐NO ☐ Unknown	Vomiting / Nausea	☐YES ☐NO ☐ Unknown
Ear pain	☐YES ☐NO ☐ Unknown	Diarrhoea	☐YES ☐NO ☐ Unknown
Wheezing	☐YES ☐NO ☐ Unknown	Conjunctivitis	☐YES ☐NO ☐ Unknown
Chest pain	☐YES ☐NO ☐ Unknown	Skin rash	☐YES ☐NO ☐ Unknown
Muscle aches (Myalgia)	☐YES ☐NO ☐ Unknown	Skin ulcers	☐YES ☐NO ☐ Unknown
Joint pain (Arthralgia)	☐YES ☐NO ☐ Unknown	Lymphadenopathy	☐YES ☐NO ☐ Unknown
Fatigue / Malaise	☐YES ☐NO ☐ Unknown	Bleeding (Haemorrhage)	☐YES ☐NO ☐ Unknown
Shortness of breath (Dyspnoea)	☐YES ☐NO ☐ Unknown	If Bleeding: specify site(s):	________________________<br>________________________

CO-MORBIDITIES *(existing prior to admission)*

Chronic cardiac disease, including congenital heart disease *(not hypertension)*	☐YES ☐NO ☐N/A	Obesity *(as defined by clinical staff)*	☐YES ☐NO ☐N/A
Chronic pulmonary disease *(not asthma)*	☐YES ☐NO ☐N/A	Diabetes with complications	☐YES ☐NO ☐N/A
Asthma *(physician diagnosed)*	☐YES ☐NO ☐N/A	Diabetes without complications	☐YES ☐NO ☐N/A
Chronic kidney disease	☐YES ☐NO ☐N/A	Rheumatologic disorder	☐YES ☐NO ☐N/A
Moderate or severe liver disease	☐YES ☐NO ☐N/A	Dementia	☐YES ☐NO ☐N/A
Mild liver disease	☐YES ☐NO ☐N/A	Malnutrition	☐YES ☐NO ☐N/A
Chronic neurological disorder	☐YES ☐NO ☐N/A	Smoking	☐YES ☐Never smoked ☐Former smoker
Malignant neoplasm	☐YES ☐NO ☐N/A	Other relevant risk factor	☐YES ☐NO ☐N/A
Chronic hematologic disease	☐YES ☐NO ☐N/A	If yes, specify:____	
AIDS / HIV	☐YES ☐NO ☐N/A	____	

Pre-admission treatment

Treated with immunosuppressants, including oral (not inhaled) corticosteroids prior to admission?	☐YES ☐NO ☐ N/A
Treated with anti-infectives for this illness episode prior to admission?	☐YES ☐NO ☐ N/A If yes, specify:____________________<br>____

ISARIC/WHO Clinical Characterisation Protocol for Severe Emerging Infections

SARI Case Report Form

CORE CASE RECORD FORM

CURRENT MEDICATION
Record medication the patient is currently taking or has taken within the past 14 days

Medication name *(generic name preferred)*	Dose	*Dose Frequency*	Route of administration
		☐q.d - once a day ☐b.i.d - twice a day<br>☐t.i.d - three times a day ☐q.i.d - four times a day<br>☐q.h.s - before bed ☐5X a day - five times a day<br>☐ q.4h - every four hours ☐ q.6h - every six hours<br>☐q.o.d - every other day ☐prn - as needed<br>☐Other frequency Specify Other:_______________________	☐IV ☐oral ☐inhaled<br>☐other ☐unknown<br>Specify Other:___________
		☐q.d - once a day ☐b.i.d - twice a day<br>☐t.i.d - three times a day ☐q.i.d - four times a day<br>☐q.h.s - before bed ☐5X a day - five times a day<br>☐ q.4h - every four hours ☐ q.6h - every six hours<br>☐q.o.d - every other day ☐prn - as needed<br>☐Other frequency Specify Other:_______________________	☐IV ☐oral ☐inhaled<br>☐other ☐unknown<br>Specify Other:___________
		☐q.d - once a day ☐b.i.d - twice a day<br>☐t.i.d - three times a day ☐q.i.d - four times a day<br>☐q.h.s - before bed ☐5X a day - five times a day<br>☐ q.4h - every four hours ☐ q.6h - every six hours<br>☐q.o.d - every other day ☐prn - as needed<br>☐Other frequency Specify Other:_______________________	☐IV ☐oral ☐inhaled<br>☐other ☐unknown<br>Specify Other:___________
		☐q.d - once a day ☐b.i.d - twice a day<br>☐t.i.d - three times a day ☐q.i.d - four times a day<br>☐q.h.s - before bed ☐5X a day - five times a day<br>☐ q.4h - every four hours ☐ q.6h - every six hours<br>☐q.o.d - every other day ☐prn - as needed<br>☐Other frequency Specify Other:_______________________	☐IV ☐oral ☐inhaled<br>☐other ☐unknown<br>Specify Other:___________
		☐q.d - once a day ☐b.i.d - twice a day<br>☐t.i.d - three times a day ☐q.i.d - four times a day<br>☐q.h.s - before bed ☐5X a day - five times a day<br>☐ q.4h - every four hours ☐ q.6h - every six hours<br>☐q.o.d - every other day ☐prn - as needed<br>☐Other frequency Specify Other:_______________________	☐IV ☐oral ☐inhaled<br>☐other ☐unknown<br>Specify Other:___________

CORE CASE RECORD FORM

ADMISSION AND DAILY TREATMENT *(complete every line)*:

DATE OF ASSESSMENT *(DD/MM/YYYY)*: [_D_][_D_]/[_M_][_M_]/[_2_][_0_][_Y_][_Y_]

Record the worst value between 00:00 to 24:00 on day of assessment *(if Not Available write 'N/A')*:

Current admission to ICU/ITU/IMC/HDU? ☐YES ☐NO ☐N/A

Done ☐YES ☐NO FiO_2 *(0.21-1.0)* [___].[___][___] or [___][___]L/min

Done ☐YES ☐NO SaO_2 [___][___][___]%

Done ☐YES ☐NO PaO_2 *at time of FiO_2 above* [___][___][___] ☐kPa *or* ☐mmHg

Done ☐YES ☐NO PaO_2 sample type: ☐ Arterial ☐ Venous ☐ Capillary ☐N/A

Done ☐YES ☐NO From same blood gas record as PaO_2 PCO_2 ____________☐kPa *or* ☐mmHg

Done ☐YES ☐NO pH _____________

Done ☐YES ☐NO HCO_3- ___________mEq/L

Done ☐YES ☐NO Base excess __________ mmol/L

Done ☐YES ☐NO AVPU Alert[___] Verbal[___] Pain [___] Unresponsive[___] Glasgow Coma Score (GCS / 15) [___][___]

Done ☐YES ☐NO Systolic Blood Pressure [___][___][___]mmHg

Done ☐YES ☐NO Diastolic Blood Pressure [___][___][___]mmHg

Done ☐YES ☐NO Mean Arterial Blood Pressure [___][___][___]mmHg

Done ☐YES ☐NO Urine flow rate [___][___][___][___][___]mL/24 hours ☐ Check if estimated

Is the patient currently receiving, or has received (from 00:00 to 24:00) on day of assessment) *(apply to all questions in this section)*

Non-invasive ventilation *(e.g. BIPAP, CPAP)*? ☐ YES ☐ NO ☐ N/A **Invasive ventilation?** ☐ YES ☐ NO ☐N/A

High-flow nasal canula oxygen therapy? ☐YES ☐NO ☐N/A **ECLS/ECMO?** ☐ YES ☐ NO ☐ N/A

Dialysis/Hemofiltration? ☐YES ☐NO ☐N/A

Any vasopressor/inotropic support? ☐ YES ☐ NO *(if NO, answer the next 3 questions NO)* ☐N/A

Dopamine <5µg/kg/min OR Dobutamine OR milrinone OR levosimendan: ☐ YES ☐ NO

Dopamine 5-15µg/kg/min OR Epinephrine/Norepinephrine < 0.1µg/kg/min OR vasopressin OR phenylephrine: ☐ YES ☐ NO

Dopamine >15µg/k/min OR Epinephrine/Norepinephrine > 0.1µg/kg/min: ☐ YES ☐ NO

Neuromuscular blocking agents? ☐ YES ☐ NO ☐ N/A **Inhaled Nitric Oxide?** ☐YES ☐NO ☐N/A

Prone positioning? ☐ YES ☐ NO ☐ N/A **Tracheostomy inserted?** ☐YES ☐NO ☐N/A

Other intervention or procedure: ☐ YES ☐ NO ☐ N/A **If YES, Specify**: ____

DAILY CASE RECORD FORM

DAILY LABORATORY RESULTS

(DD/MM/YYYY): [_D_][_D_]/[_M_][_M_]/[_2_][_0_][_Y_][_Y_]

Record the worst value between 00:00 to 24:00 on day of assessment *(if Not Available write 'N/A')*:

Done ☐YES ☐NO **Haemoglobin** _______ ☐g/L *or* ☐g/dL

Done ☐YES ☐NO **WBC count** ___________ ☐x10^9/L *or* ☐x10^3/µL

Done ☐YES ☐NO **Lymphocyte count** ______________ ________ ☐cells/ µL

Done ☐YES ☐NO **Neutrophil count** _________________ _____ ☐ cells/ µL

Done ☐YES ☐NO **Haematocrit** [___][___]%

Done ☐YES ☐NO **Platelets** ___________ ☐x10^9/L *or* ☐x10^3/µL **Done** ☐YES ☐NO **APTT/APTR** __________

Done ☐YES ☐NO **PT** ___________ seconds *or* **Done** ☐YES ☐NO **INR**____________

Done ☐YES ☐NO **ALT/SGPT** _________ U/L **Done** ☐YES ☐NO **Total Bilirubin** _______☐µmol/L *or* ☐mg/dL

Done ☐YES ☐NO **AST/SGOT** _________ U/L **Done** ☐YES ☐NO **Glucose** _________☐mmol/L *or* ☐mg/dL

Done ☐YES ☐NO **Blood Urea Nitrogen (urea)** ____________ ☐mmol/L *or* ☐mg/dL

Done ☐YES ☐NO **Lactate** ___________☐mmol/L *or* ☐mg/dL

Done ☐YES ☐NO **LDH** [___][___][___].[___]_U/L

Done ☐YES ☐NO **Creatinine Kinase (CPK)** [___][___][___].[___]_U/L

Done ☐YES ☐NO **Creatinine** _____________ ☐µmol/L *or*☐mg/dL

Done ☐YES ☐NO **Sodium** [___][___][___][___] mEq/L

Done ☐YES ☐NO **Potassium** [___][___].[___] mEq/L

Done ☐YES ☐NO **Procalcitonin** [___][___].[___][___]ng/mL

Done ☐YES ☐NO **CRP** [___][___][___].[___] mg/L

Chest X-Ray /CT performed? ☐YES ☐NO ☐N/A **IF Yes: Were infiltrates present?** ☐YES ☐NO ☐N/A

ISARIC

ISARIC/WHO Clinical Characterisation Protocol for Severe Emerging Infections
SARI Case Report Form
OUTCOME CASE RECORD FORM

PATHOGEN TESTING

Was pathogen testing done during this illness episode? ☐YES ☐NO ☐N/A

Influenza : ☐ YES- Confirmed ☐ YES- Probable ☐ NO **If YES:** ☐ A/H3N2 ☐ A/H1N1pdm09 ☐ A/H7N9

☐ A/H5N1 ☐ A not typed, other A ☐_____________ ☐ B not typed ☐ Other type (specify):______________

Coronavirus: ☐ YES- Confirmed ☐ YES- Probable ☐ NO **If YES:** ☐ MERS CoV ☐ 2019nCoV

☐ Other CoV (specify): _____________________

RSV: ☐ YES- Confirmed ☐ YES- Probable ☐ NO

Adenovirus: ☐ YES- Confirmed ☐ YES- Probable ☐ NO

Bacteria: : ☐ YES – confirmed: specify : ___________________________ ☐ No

Other : ☐ YES- Confirmed ☐ YES- Probable ☐ NO

If yes Other, specify____

Clinical pneumonia: ☐ YES ☐ NO ☐ Unknown **If NONE OF THE ABOVE: Suspected Non-infective:** ☐ YES

LIMS Number	Collection Date *(DD/MM/YYYY)*	Bio specimen Type	Laboratory Test Method	Result	Pathogen Tested/Detected
	__ __/__ __/20__ __	☐Nasal/NP swab<br>☐Throat swab<br>☐Combined nasal/NP+throat swab<br>☐Sputum ☐BAL<br>☐ETA ☐Urine<br>☐Feces/rectal swab<br>☐Blood<br>☐Other, *Specify:* ___________________	☐PCR<br>☐Culture<br>☐Other, *Specify:* _____________	☐Positive<br>☐Negative<br>☐N/A	_______________
	__ __/__ __/20__ __	☐Nasal/NP swab<br>☐Throat swab<br>☐Combined nasal/NP+throat swab<br>☐Sputum ☐BAL<br>☐ETA ☐Urine<br>☐Feces/rectal swab<br>☐Blood<br>☐Other, *Specify:* ___________________	☐PCR<br>☐Culture<br>☐Other, *Specify:* _____________	☐Positive<br>☐Negative<br>☐N/A	_______________
	__ __/__ __/20__ __	☐Nasal/NP swab<br>☐Throat swab<br>☐Combined nasal/NP+throat swab<br>☐Sputum ☐BAL<br>☐ETA ☐Urine<br>☐Feces/rectal swab<br>☐Blood<br>☐Other, *Specify:* ___________________	☐PCR<br>☐Culture<br>☐Other, *Specify:* _____________	☐Positive<br>☐Negative<br>☐N/A	_______________

	__ __/__ __/20__ __	☐Nasal/NP swab<br>☐Throat swab<br>☐Combined nasal/NP+throat swab<br>☐Sputum ☐BAL<br>☐ETA ☐Urine<br>☐Feces/rectal swab<br>☐Blood<br>☐Other, *Specify:* ____________________	☐PCR<br>☐Culture<br>☐Other, *Specify:* _____________	☐Positive<br>☐Negative<br>☐N/A	_______________ __
	__ __/__ __/20__ __	☐Nasal/NP swab<br>☐Throat swab<br>☐Combined nasal/NP+throat swab<br>☐Sputum ☐BAL<br>☐ETA ☐Urine<br>☐Feces/rectal swab<br>☐Blood<br>☐Other, *Specify:* ____________________	☐PCR<br>☐Culture<br>☐Other, *Specify:* _____________	☐Positive<br>☐Negative<br>☐N/A	_______________ __

MEDICATION: While hospitalised or at discharge, were any of the following administered?

Antiviral agent? ☐YES ☐NO ☐ N/A **If YES, specify:** ORibavirin OLopinavir/Ritonavir OInterferon alpha OInterferon beta

ONeuraminidase inhibitor **if YES:** Which ________________ OOther ________________

Antibiotic? ☐YES ☐NO ☐N/A **If YES:** specify type(s): ________________ _____________ _____________

Corticosteroid? ☐YES ☐NO ☐N/A **If YES, Route:** ☐ Oral ☐ Intravenous ☐ Inhaled

If YES, please provide type and dose: ___________

Antifungal agent? ☐YES ☐NO ☐N/A **If YES:** which ________________

TREATMENT: At ANY time during hospitalisation, did the patient receive/undergo:

ICU or High Dependency Unit admission? ☐YES ☐NO ☐N/A………….. If YES, total duration: _________days

Date of ICU admission:[_D_][_D_]/[_M_][_M_]/[_2_][_0_][_Y_][_Y_] ☐N/A

ICU discharge date: [_D_][_D_]/[_M_][_M_]/[_2_][_0_][_Y_][_Y_] ☐N/A

Oxygen therapy? ☐YES ☐NO ☐N/A

Non-invasive ventilation? *(e.g. BIPAP, CPAP)* ☐YES ☐NO ☐N/A

Invasive ventilation *(Any)***?** ☐YES ☐NO ☐N/A If YES, total duration: _________days

Prone Ventilation? ☐YES ☐NO ☐N/A

Inhaled Nitric Oxide? ☐YES ☐NO ☐N/A

Tracheostomy inserted? ☐YES ☐NO ☐N/A

Extracorporeal (ECMO) support? ☐YES ☐NO ☐N/A If YES, total duration: _________days

Renal replacement therapy (RRT) or dialysis? ☐YES ☐NO ☐N/A

Inotropes/vasopressors? ☐YES ☐NO ☐N/A If YES, total duration: _________days

OTHER intervention or procedure *(please specify)***:** ____

COMPLICATIONS: At any time during hospitalisation did the patient experience:			
Viral pneumonia	☐YES ☐NO ☐N/A	Cardiac arrest	☐YES ☐NO ☐N/A
Bacterial pneumonia	☐YES ☐NO ☐N/A	Bacteraemia	☐YES ☐NO ☐N/A
Acute Respiratory Distress Syndrome	☐YES ☐NO ☐N/A	Coagulation disorder / Disseminated Intravascular Coagulation	☐YES ☐NO ☐N/A
Cryptogenic organizing pneumonia (COP)	☐YES ☐NO ☐N/A	Anaemia	☐YES ☐NO ☐N/A
Pneumothorax	☐YES ☐NO ☐N/A	Rhabdomyolysis / Myositis	☐YES ☐NO ☐N/A
Pleural effusion	☐YES ☐NO ☐N/A	Acute renal injury/acute renal failure	☐YES ☐NO ☐N/A
Bronchiolitis	☐YES ☐NO ☐N/A	Gastrointestinal haemorrhage	☐YES ☐NO ☐N/A
Meningitis / Encephalitis	☐YES ☐NO ☐N/A	Pancreatitis	☐YES ☐NO ☐N/A
Seizure	☐YES ☐NO ☐N/A	Liver dysfunction	☐YES ☐NO ☐N/A
Stroke / Cerebrovascular accident	☐YES ☐NO ☐N/A	Hyperglycaemia	☐YES ☐NO ☐N/A
Congestive heart failure	☐YES ☐NO ☐N/A	Hypoglycaemia	☐YES ☐NO ☐N/A
Endocarditis/Myocarditis/ Pericarditis	☐YES ☐NO ☐N/A	Other	☐YES ☐NO ☐N/A
Cardiac arrhythmia	☐YES ☐NO ☐N/A	If yes, specify:______________________________________	
Cardiac ischemia	☐YES ☐NO ☐N/A	____	

STUDY PARTICIPATION

Is / Has the participant being recruited to a trial or multi-centre study during the period of their current illness (including initiation in the community and hospital)? ☐ YES ☐ NO

IF YES , specify
Name of study_________________________________
Study Participant ID _________________________

Add another study? ☐ YES ☐ NO
IF YES , specify
Name of study_________________________________
Study Participant ID _________________________

Add another study? ☐ YES ☐ NO
IF YES , specify
Name of study_________________________________
Study Participant ID _________________________

OUTCOME CASE RECORD FORM

OUTCOME

Outcome: ☐ Discharged alive expected to survive ☐ Hospitalization ☐ Transfer to other facility ☐ Death

☐ Palliative discharge ☐ Unknown

Outcome date: [_D_][_D_]/[_M_][_M_]/[_2_][_0_][_Y_][_Y_] ☐ N/A

If Discharged alive:

Ability to self-care at discharge versus before illness: ☐ Same as before illness ☐ Worse ☐ Better ☐ N/A

If Discharged alive: Post-discharge treatment:

Oxygen therapy? ☐ YES ☐ NO ☐ N/A **Dialysis/renal treatment?** ☐ YES ☐ NO ☐ N/A

Other intervention or procedure? ☐ YES ☐ NO ☐ N/A

If YES: Specify *(multiple permitted):* ________________________________

If Transferred: Facility name: ____ ☐ N/A

If Transferred: Is the transfer facility a study site? ☐ YES ☐ NO ☐ N/A

If a Study Site: Participant ID # at new facility: ☐ Same as above

☐ Different: [___][___][___] – [___][___][___][___] ☐N/A

ISARIC

TRAVEL AND ANIMAL EXPOSURE CASE RECORD FORM

TRAVEL (addition countries to those listed on COR CRF page 2 of 3)

Did the patient travel in the 14 days prior to first symptom onset*:*

Country:_________________ City/Geographic area: ____________________ Return Date *(DD/MM/20YY):* ____ /____ /20_____

Country:_________________ City/Geographic area: ____________________ Return Date *(DD/MM/20YY):* ____ /____ /20_____

Country:_________________ City/Geographic area: ____________________ Return Date *(DD/MM/20YY):* ____ /____ /20_____

Country:_________________ City/Geographic area: ____________________ Return Date *(DD/MM/20YY):* ____ /____ /20_____

Country:_________________ City/Geographic area: ____________________ Return Date *(DD/MM/20YY):* ____ /____ /20_____

ANIMAL EXPOSURES:

Did the patient have contact with live/dead animals, raw meat or insect bites in the 14 days prior to first symptom onset?

If YES, specify the animal/insect, type of contact and date of exposure (DD/MM/YYYY). *(Complete each line)*

Bird/Aves (e.g. chickens, turkeys, ducks)	☐YES ☐NO ☐N/A	
Bat	☐YES ☐NO ☐N/A	
Livestock (e.g. goats, cattle, camels)	☐YES ☐NO ☐N/A	
Horse	☐YES ☐NO ☐N/A	
Hare/ Rabbit	☐YES ☐NO ☐N/A	
Pigs	☐YES ☐NO ☐N/A	
Non-human primates	☐YES ☐NO ☐N/A	
Rodent (e.g. rats, mice, squirrels)	☐YES ☐NO ☐N/A	
Insect or tick bite (e.g. tick, flea, mosquito)	☐YES ☐NO ☐N/A	
Reptile / Amphibian	☐YES ☐NO ☐N/A	
Domestic animals living in his/her home (e.g. cats, dogs, other)	☐YES ☐NO ☐N/A	
Animal faeces or nests	☐YES ☐NO ☐N/A	
Sick animal or dead animal	☐YES ☐NO ☐N/A	
Raw animal meat / animal blood	☐YES ☐NO ☐N/A	
Skinned, dressed or eaten wild game	☐YES ☐NO ☐N/A	
Visit to live animal market, farm or zoo	☐YES ☐NO ☐N/A	
Participated in animal surgery or necropsy	☐YES ☐NO ☐N/A	
Other animal contacts:	☐YES ☐NO ☐N/A	
